# Multivariate Imaging Genetics Study of MRI Gray Matter Volume and SNPs Reveals Biological Pathways Correlated with Brain Structural Differences in Attention Deficit Hyperactivity Disorder

**DOI:** 10.3389/fpsyt.2016.00128

**Published:** 2016-07-25

**Authors:** Sabin Khadka, Godfrey D. Pearlson, Vince D. Calhoun, Jingyu Liu, Joel Gelernter, Katie L. Bessette, Michael C. Stevens

**Affiliations:** ^1^Olin Neuropsychiatry Research Center, Institute of Living, Hartford HealthCare, Hartford, CT, USA; ^2^Department of Psychiatry, Yale University School of Medicine, New Haven, CT, USA; ^3^Department of Neurobiology, Yale University School of Medicine, New Haven, CT, USA; ^4^The Mind Research Network, Albuquerque, NM, USA; ^5^Department of Electrical and Computer Engineering, University of New Mexico, Albuquerque, NM, USA

**Keywords:** genetics, ADHD, sMRI, parallel ICA, neurodevelopmental disorder

## Abstract

**Background:**

Attention deficit hyperactivity disorder (ADHD) is a prevalent neurodevelopmental disorder affecting children, adolescents, and adults. Its etiology is not well understood, but it is increasingly believed to result from diverse pathophysiologies that affect the structure and function of specific brain circuits. Although one of the best-studied neurobiological abnormalities in ADHD is reduced fronto-striatal-cerebellar gray matter (GM) volume, its specific genetic correlates are largely unknown.

**Methods:**

In this study, T1-weighted MR images of brain structure were collected from 198 adolescents (63 ADHD-diagnosed). A multivariate parallel independent component analysis (Para-ICA) technique-identified imaging genetic relationships between regional GM volume and single nucleotide polymorphism data.

**Results:**

Para-ICA analyses extracted 14 components from genetic data and 9 from MR data. An iterative cross-validation using randomly chosen subsamples indicated acceptable stability of these ICA solutions. A series of partial correlation analyses controlling for age, sex, and ethnicity revealed two genotype–phenotype component pairs significantly differed between ADHD and non-ADHD groups, after a Bonferroni correction for multiple comparisons. The brain phenotype component not only included structures frequently found to have abnormally low volume in previous ADHD studies but was also significantly associated with ADHD differences in symptom severity and performance on cognitive tests frequently found to be impaired in patients diagnosed with the disorder. Pathway analysis of the genotype component identified several different biological pathways linked to these structural abnormalities in ADHD.

**Conclusion:**

Some of these pathways implicate well-known dopaminergic neurotransmission and neurodevelopment hypothesized to be abnormal in ADHD. Other more recently implicated pathways included glutamatergic and GABA-eric physiological systems; others might reflect sources of shared liability to disturbances commonly found in ADHD, such as sleep abnormalities.

## Introduction

Attention deficit hyperactivity disorder (ADHD) is a complex neurodevelopmental disorder (1) whose etiology is not fully understood. In attempts to understand its strong (70–80%) heritability (2, 3), linkage studies have identified potential susceptibility loci on reported chromosomal regions, including 16p13 and 17p11 (3). Candidate-gene studies have implicated some single nucleotide polymorphisms (SNPs) associated with dopaminergic (DA), serotonergic, and noradrenergic systems implicated by pharmacologic response and neuroimaging research (3, 4). A few genome-wide association studies (GWAS) have found evidence that *CDH13, GFOD1, FBXO33*, and *SLC9A9* genes might be associated with ADHD (3, 5). Quantitative trait analysis of ADHD has shown associations between inattentive and hyperactive/impulsive symptoms and variations in glutamate receptor *GRIN2B* subunit genes (6). Also, *GRIN2A* and *GRIN2B* are reported to play role in neurodevelopment (7). While these findings represent a starting place, ADHD is believed to be a polygenic disorder that arises from the contributions of numerous known and yet-to-be-identified gene variants (8), along with noteworthy evidence for social, environmental, and/or gene × environment interactions (9, 10).

For such a complex disorder, simply identifying associations between genes and the broad diagnostic phenotype might not increase understanding as precisely or as rapidly as identifying links between the genes and specific features of the disorder, such as neuroimaging-measured brain structure (11). Meta-analyses of ADHD brain structure studies have revealed that ADHD samples often show reduced total and right cerebral gray matter (GM), cerebellum, right caudate, right putamen, and globus pallidus volumes (12, 13). Also, the parietal cortex and hippocampus are often, though less consistently, found to be abnormal in ADHD (10). One of the most reliable findings in ADHD is reduced frontal lobe volume or cortical thickness (10, 12–14) particularly in the right frontal lobe, which includes brain regions linked to the types of cognitive and executive impairments frequently found in ADHD (15).

Neuroimaging genetics approaches offer potential understanding of biological pathways related to numerous, likely interacting genes and specific mechanisms of brain growth and function that contribute to inherited behavioral and neuropsychiatric diseases. However, it remains statistically challenging to identify such genes. Univariate GWAS methods are constrained by large sample size requirements to detect the weak effects characteristic of common disease/common variant models, given the need to Bonferroni correction for number of SNPs evaluated. In recent years, multivariate analysis techniques, such as parallel independent component analysis (Para-ICA), have been developed. These techniques identify relationships between clusters of interrelated SNPs and complex phenotypic characteristics (e.g., brain structure) in a data-driven manner (16, 17). Para-ICA has been used successfully in imaging genetics studies (18) to yield robust, theoretically informative results with practical sample sizes (19, 20). Such multivariate techniques have a useful role in discovering likely relationships between genes and neurobiology within a psychiatric disorder, which then can be explored using conventional genetic approaches. Moreover, Para-ICA is particularly well suited in identifying and then annotating aggregates (or “networks”) of genes that contribute to particular physiological pathways. Pathway analysis using currently available maps [e.g., Kyoto Encyclopedia of Genes and Genomes (KEGG)] (21) of molecular interactions that could underlie biological processes or disease might rapidly advance our understanding of disorder pathophysiology. For instance, ADHD researchers have found that specific physiological pathways are linked to the broad ADHD phenotype (22), specific ADHD symptoms (23), or cognitive performance patterns within ADHD samples (24).

Because GWAS analysis are beyond the capability of the typical sample sizes collected in neuroimaging studies, we are not interested in attempting to link genes to broad ADHD behavioral phenotype. However, Para-ICA is ideally suited for identifying novel brain structure intermediate phenotypes in sample of modest size by linking aggregates of SNPs to specific GM volume characteristics already known to be relevant to ADHD. We used Para-ICA to elucidate the relationships between regional GM measurements previously found to be abnormal in ADHD and clusters of SNPs from an arrayof >240,000 putatively functional exomic markers. Specifically, we assessed genetic relationships of structural GM in DSM-IV-combined subtype ADHD with relatively low rates of psychiatric comorbidity and healthy comparison adolescents, simultaneously, by identifying what aspects of brain structure or genotype covaried systematically across the sample to form independent component (IC) aggregates. The study used Illumina HumanExome-12v1-2 chip (Illumina, San Diego, CA, USA) for genotyping and a voxel-based morphometry (VBM) approach (25) to characterize participants’ voxel-wise GM volume obtained from T1-weighted MRI scans of brain structure. The content of these components is determined from natural aggregation found within each datatype. Thus, it is not possible to specify *a priori* the content of different components within the genetic or the neuroimaging modalities. Instead, components are assigned simple names for convenient labeling (e.g., G1, G2, etc. for genetic data or S1, S2, etc. for brain structure data) so they can be later described in detail after group-based hypothesis-testing, which determines their relevance to the disorder. To identify genetic neuroimaging relationships that differed between ADHD and non-ADHD, we extracted subject-dependent loading coefficients (LCs) of each IC for random effects statistical testing. Finally, for those component pairs (e.g., G1–S2) that differed between study groups, we characterized various molecular biological pathways associated with detected gene aggregates to provide information about possible physiological processes that might have given rise to the ADHD brain structure abnormalities detected. We hypothesized that brain phenotype component depicting ADHD-relevant regions (fronto-striatal, fronto-parietal, or cerebellar) would be associated with specific gene networks related to brain development and catecholaminergic neurotransmission. Furthermore, we predicted that ADHD subjects would show differences in both brain phenotypes and gene networks when compared to healthy controls. To enrich results interpretation, *post hoc* tests explored relationships between Para-ICA-derived LCs and ADHD clinical features.

## Materials and Methods

All study procedures were approved by Hartford Hospital’s Institutional Review Board. Written permission was obtained from parents/legal guardians of all participants, and assent was obtained from all participants under the age of 18. The sample comprised 63 community-recruited patients diagnosed with the combined subtype of ADHD (DSM-IV 314.01) and 135 healthy comparison participants. Psychiatric diagnoses for research purposes of all DSM-IV Axis I disorders were made using the Kiddie-Schedule for Affective Disorders and Schizophrenia-Present and Lifetime version (K-SADS-PL) (26) conducted by trained clinical research staff, under the supervision of a licensed clinical psychologist (Michael C. Stevens). Separate collateral interviews with at least one parent/guardian were incorporated into diagnostic decisions made after synthesizing information in weekly research group meetings. Participants were excluded if they (a) had lifetime history of bipolar disorder, psychotic disorder, obsessive–compulsive disorder, PTSD, Tourette’s disorder, pervasive developmental disorder; (b) had current DSM-IV substance dependence, major depressive disorder, or anxiety disorder; (c) had IQ estimate <80 determined by Wechsler abbreviated Scale of Intelligence; or (d) were taking Wellbutrin (Buproprion), Strattera (Atomoxetine HCl), Cylert (Pemoline), or Provigil (Modafinil). Sample characteristics are shown in Table S1 in Supplementary Material. Clinical assessment was supplemented with parent-reported Brown attention deficit disorder (ADD) scale (27) scores and performance on Conner’s continuous performance test (CPT-II) (28).

### Structural MRI and Data Preparation

Structural MR images were obtained using a Siemens 3T Allegra MRI scanner at Olin Neuropsychiatry Research Center at Hartford Hospital (3D MPRAGE; TR = 2300 ms, TE = 2.74 ms, TI = 900 ms, flip angle = 8°, FOV = 17 mm × 256 mm, matrix = 176 × 256 × 176, voxel size = 1 mm × 1 mm × 1 mm, pixel bandwidth = 190 Hz; 7.09 min). MR images were examined and processed using VBM8 toolbox (29) with default settings, as follows: (i) bias-correction, (ii) tissue classification, (iii) spatial normalization to Montreal Neurological Institute (MNI) space, (iv) high dimensional non-linear diffeomorphic anatomical registration through exponentiated lie algebra (DARTEL) normalization, and (v) finally, the normalized GM segments were modulated to correct and preserve original local volume change. The processing pipeline is explained in detail in the VBM8 tutorial.^1^ Modulated GM volumes were smoothed with 4 mm full width half maximum Gaussian kernel.

### SNPs Data Collection and Preprocessing

**Figure 1:**
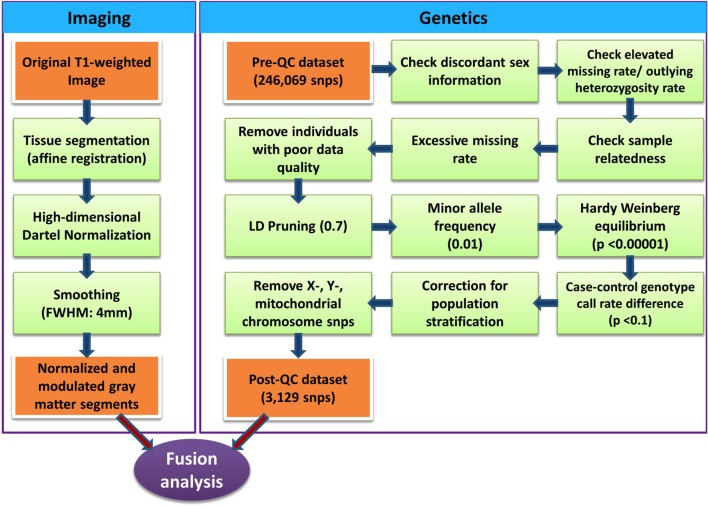
**Illustration of quality control processing for SNP data and structural MR imaging data**.

Genomic DNA was extracted from saliva collected from each participant using Oragene collection kits (30). The Illumina HumanExome-12v1-2 chip was used for genotyping. Genotype data were preprocessed using PLINK software (31) following quality control steps (32) detailed in Figure 1. SNPs in high linkage disequilibrium (LD) were removed (window size of SNPs = 50, number of SNPs to shift the window at each step = 5 and *r^2^* = 0.7) to increase independence between markers. Custom Matlab scripts using an algorithm similar to EIGENSTRAT (33) performed principal component analysis of the genetic dataset. To correct for population stratification bias, genetic data were corrected using top two eigenvectors that showed significance association with self-reported race/ethnicity. Logistic regression (adjusting for age, sex, and top two eigenvectors representing self-reported ethnicity) was performed to clean the data and reduce the number of SNPs. SNPs showing association with ADHD (uncorrected *p*-value < 0.1) were considered for further analysis. The total number of SNPs further considered with Para-ICA was 3139 (see Table S2 in Supplementary Material for complete list of SNPs and their associated genes used for Para-ICA). The entire set of markers was annotated to genes based on their coordinates using ANNOVAR software (34).

### Parallel Independent Component Analysis

Parallel independent component analysis between SNPs data and modulated GM volume used the Fusion ICA Toolbox^2^ within Matlab 7.7. Practical implementation of Para-ICA for gene and MRI data is explained in detail in a recent review paper (18), and an overview is shown in Figure S1 in Supplementary Material. Because our study goals using Para-ICA technique have numerous, important differences from a GWAS analysis approach that has been highly influential in psychiatric genetics, it is useful to briefly review the methods involved to highlight the different expectations involved. Para-ICA is a data-driven approach that estimates maximally ICs within gene and brain phenotype data separately and also maximizes the association between modalities using an entropy term based on information theory (16–18). The goal of Para-ICA is not to identify single gene association. Instead, ICA is run separately, in parallel, on both modalities to identify collections of interrelated SNPs and to isolate specific features within the brain structure maps that systematically covary across participants. Not only do the components identified by Para-ICA represent meaningful aggregates but also the number of subsequent statistical tests gets reduced substantially. Thus, Para-ICA allows us to confidently assess the relationship between modalities (e.g., genetic and MRI data) as well as group difference (e.g., patient versus controls) for each component of each modality in moderate-sized samples. As such, it is ideal to identify relationships between modalities within a specific disorder that otherwise would require tens of thousands of participants using GWAS techniques. Although techniques like Para-ICA can help to rapidly advance our understanding of complex gene–brain disorder relationships, in many applications, it should be considered exploratory, with its results needing replication.

For the current analysis, the ratio of sample size to number of SNPs (198/3139) in our study is consistent with validation work showing that Para-ICA will provide accurate results (35). The number of ICs estimated using minimum description length criteria (18, 36) was 14 for genetic data and 9 for MRI data. Importantly, because full replication was not possible given the data available, the consistency and stability of the components was examined using leave-*N*-subjects-out (5% of total subjects) cross-validation technique (18, 37), run iteratively across randomly chosen sub-samples. This reliability validation method revealed that the stability of genetic and brain phenotype components were acceptable – 70 and 90%, respectively. The LCs for each component × modality × subject were extracted, and partial correlation [controlling for age, sex, top two eigenvectors representing self-reported ethnicity, and group association vector (ADHD versus HC)] between LCs of both modalities was computed in SPSS v19.0 (IBM, Inc.). Component pairs that survived Bonferroni correction for multiple comparison [*p* < 0.05/(9 × 14)] were examined for *post hoc* pairwise group differences. To correct for gene size bias and select dominant genes in a component, gene-based association values were calculated using VEGAS software (38). To define dominant regions of component maps, an arbitrary threshold of |*z*| > 1.5 and cluster size *k* > 50 voxels was selected. To enrich possible interpretation of the ICs identified by Para-ICA, we also assessed linear associations between clinical measures (e.g., symptom sums or cognitive test scores) and Para-ICA-derived genetic and phenotype components, controlling for age and sex. Because these were exploratory *post hoc* analyses, significant correlations (*p* < 0.05, uncorrected) are reported.

### Genetic Pathway Analysis

To identify underlying biological pathways of the gene sets, we used the ConsensusPath database.^3^ Only genes that showed gene-based trait association of *p*-value < 0.05 were selected for pathway enrichment analysis. The lists of significant genes of component G2 (after gene size correction) are listed in Table S3 in Supplementary Material. The *p*-value for each pathway is calculated using a hypergeometric approach based on number of genes in both user-specified gene set and genes associated with each pathway. The significance values were FDR-adjusted to correct for multiple comparison (39).

## Results

### Genotype–Phenotype Associations

**Figure 2:**
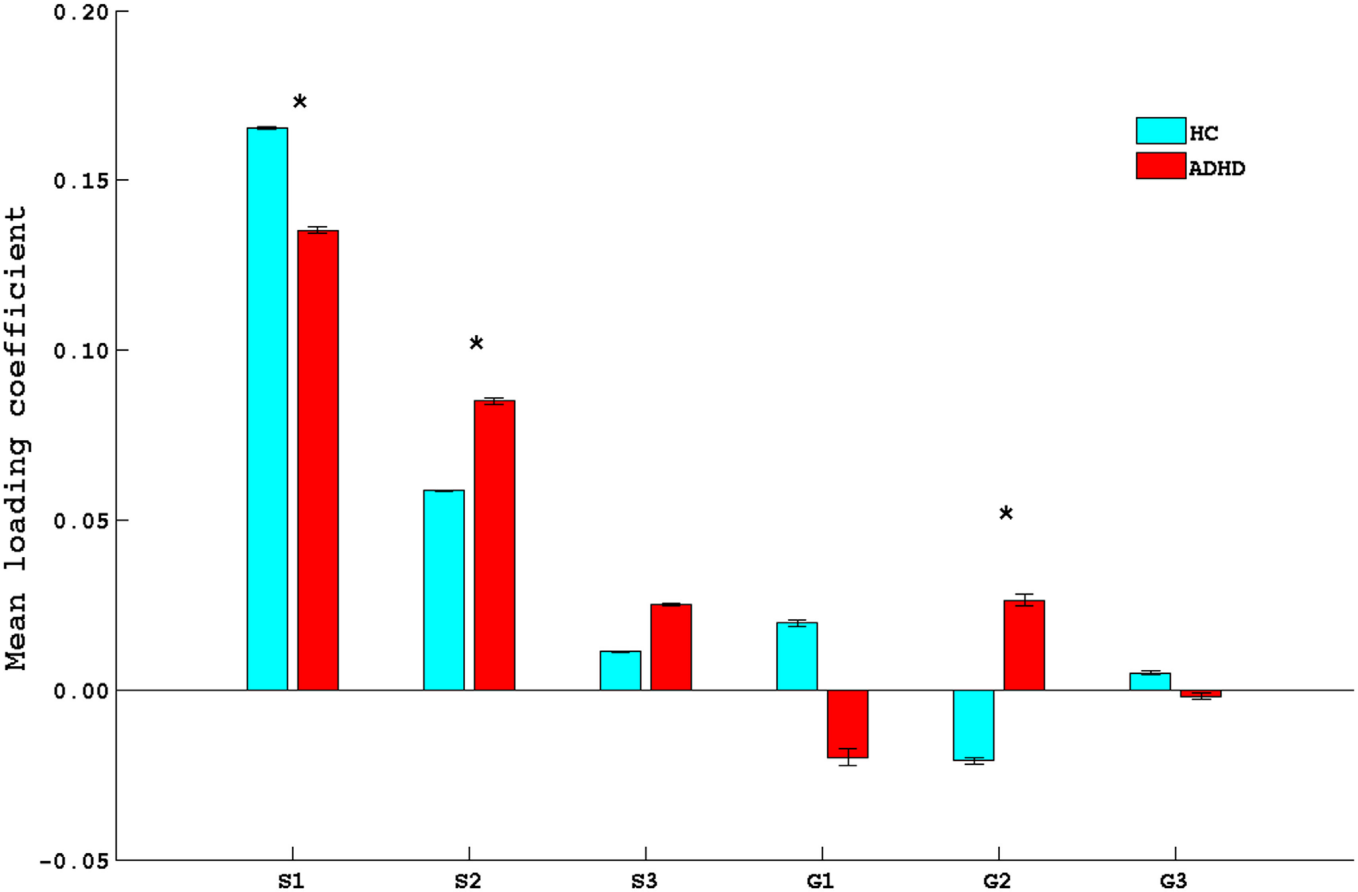
**Bar plot of mean loading coefficients of brain phenotype component and genetic component**. * indicates group differences among ADHD and HC with *p* < 0.05. HC, healthy controls; ADHD, attention deficit hyperactive disorder.

Partial correlation analysis of component coefficients revealed significant associations among five phenotype–genotype component pairs. Three brain phenotype ICs (designated as S1, S2, and S3) showed significant correlation with three genetic ICs (designated as G1, G2, and G3). Pearson’s correlation coefficients (along with their associated *p-value*) of genetic ICs and brain phenotype ICs are listed in Table 1. Of these, the S1–G2 and S2–G2 pairs showed a study group difference in LCs for both the genetic and brain phenotype components (Figure 2), making these components of primary interest to our study aims. Levene’s test for equality of variances confirmed the variance between groups were similar.

**Table 1:** **Significant brain phenotype–genetic component association**.

Brain phenotype component	Genetic component	*r*	*p*-value
S1	G1	0.34	1 × 10^−6^
S1	G2	−0.34	9 × 10^−7^
S2	G1	−0.41	2 × 10^−9^
S2	G2	0.31	1 × 10^−5^
S3	G3	−0.32	4 × 10^−6^

*S1–S3, brain phenotype independent components; G1–G3, genetic independent components; r, Pearson’s correlation*.

**Figure 3:**
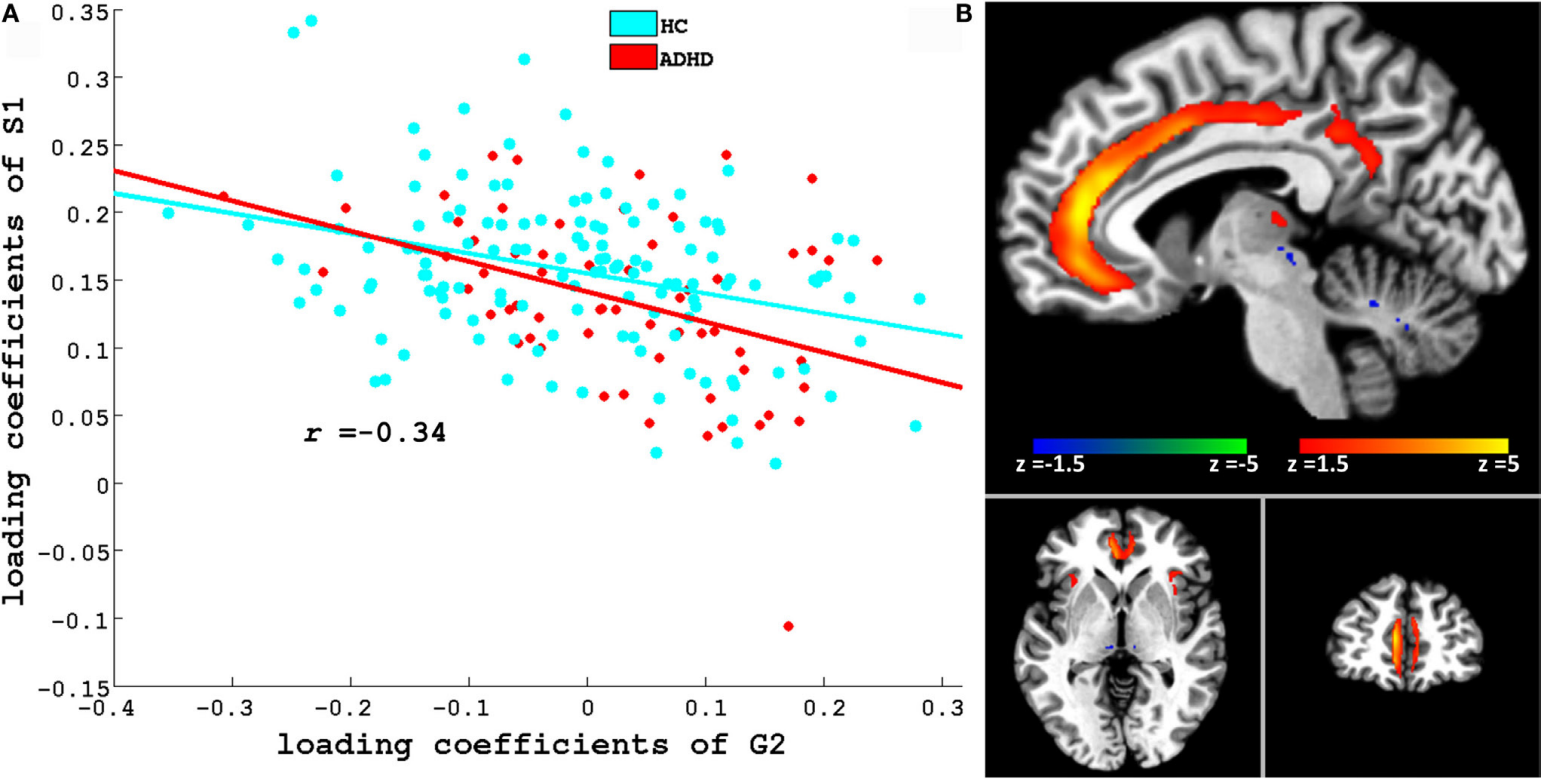
**(A)** Scatter plots of loading coefficient of brain phenotype component S1 and genetic component G2. Scatter plot and line in red and cyan color indicates ADHD and HC group, respectively. **(B)** Significant regions in S1. Brain slices shown in the above figure are *x* = −6, *y* = 46, and *z* = −3 in Montreal Neurological Institute (MNI) space.

**Figure 4:**
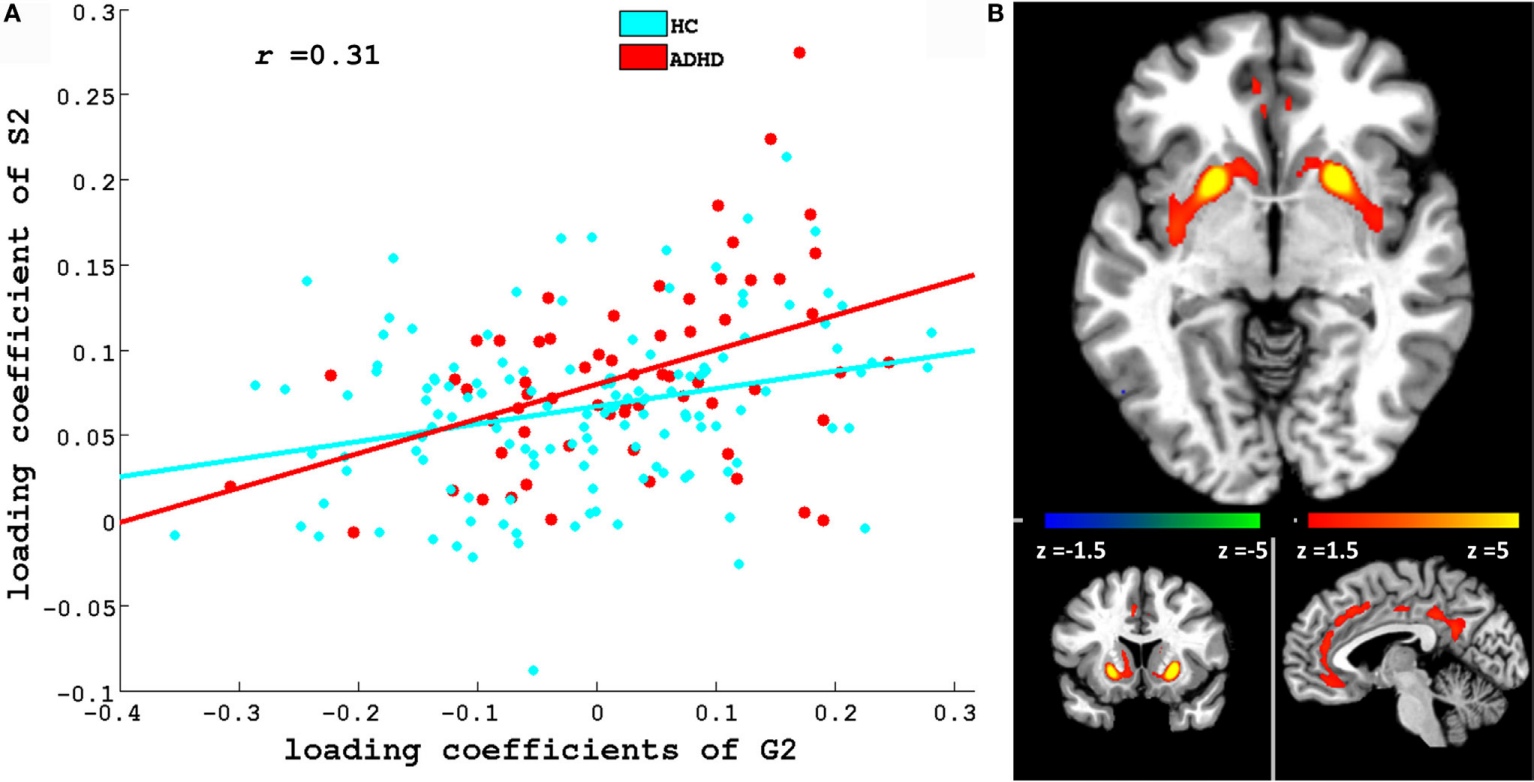
**(A)** Scatter plots of loading coefficient of brain phenotype component S2 and genetic component G2. Scatter plot and line in red and cyan color indicates ADHD and HC group, respectively. **(B)** Significant regions in S2. Brain slices shown in the above figure are *x* = −4, *y* = 13, and *z* = −6 in Montreal Neurological Institute (MNI) space.

Figures 3 and 4 depict these S1–G2 and S2–G2 component pairs. For both, ADHD participants showed a stronger genotype–phenotype relationship than non-ADHD. The significant brain regions in brain phenotype components S1 and S2 are listed in Tables S4 and S5 in Supplementary Material respectively, and are shown in Figures 3B and 4B respectively. The most prominent regions within brain phenotype component S1 were anterior/mid-cingulate gyrus and bilateral anterior insula. Bilateral basal ganglia (caudate and both dorsal and ventral putamen), mid-cingulate, and thalamus were the most strongly implicated regions in S2. Both components included other brain regions, although with lower evidence for their association with the component. S1 also contained bilateral cerebellum, posterior cingulate, thalamus, fusiform, and parahippocampus gyrus. S2 also included cerebellum, mid- and posterior cingulate, fusiform, rostral cingulate/orbitofrontal cortex, and thalamus. Many of these other regions also have been found to be structurally abnormal in ADHD. Overlap between the brain phenotype components was seen only in regions of anterior and posterior cingulate, differing in how strongly they loaded on each component (e.g., anterior cingulate loading strongest for S1; posterior cingulate for S2).

*Post hoc* testing revealed correlations between S2’s LCs and scores on the Brown ADD scale’s attention [*r* = 0.32, *p*(uncorrected) = 0.03], effort [*r* = 0.37, *p*(uncorrected) = 0.01], affect [*r* = 0.34, *p*(uncorrected) = 0.02], and working memory [*r* = 0.38, *p*(uncorrected) = 0.01] scores. The Brown ADD scale effort score also showed correlation between S1’s LCs [*r* = −0.30, *p*(uncorrected) = 0.04]. Also, the S2 LCs were correlated with the CPT-II omission [*r* = 0.45, *p*(uncorrected) = 0.002] and variability [*r* = 0.32, *p*(uncorrected) = 0.03] standardized scores. The LCs of the G1 correlated with CPT-II Omission [*r* = 0.33, *p*(uncorrected) = 0.02]. Although none of the correlations survived multiple comparison correction when tested as a complete set, these exploratory results reinforce the relevance of these brain phenotype components to clinically relevant ADHD dysfunction. ADHD symptom severity (i.e., K-SADS-PL symptom count) was uncorrelated with either genotype or phenotype LCs.

The S3–G3 pair was less informative for our primary objective. Although knowing what SNPs are associated with this collection of brain regions is valuable, neither its phenotype nor genotype component coefficients differed between groups. Brain regions comprising the S3 component are listed in Table S6 in Supplementary Material and depicted in Figure S2 in Supplementary Material. Figure S2 in Supplementary Material also depicts scatterplots of the other significantly linked brain phenotype and genotype component pairs that are not further considered.

### Pathway Enrichment Analysis

Significant KEGG pathways associated with G2-identified genoptypes along with uncorrected *p* and corrected *q* values are listed in Table 2. These included pathways involved in neurotransmission (glutamatergic synapse, DA synapse, retrogade endocannabinoid signaling, GABAergic synapse, and cholinergic synapse), neurodevelopment (Rap1 signaling, neuroactive ligand–receptor interaction), and other functions, such as circadian entrainment, insulin secretion, hypertropic cardiomyopathy, dilated cardiomyopathy, the estrogen signaling pathway, and endocytosis. As discussed below, these pathways represent a diverse mix of biological systems – some are already known to be implicated in ADHD, while others represent relatively novel findings for the disorder.

**Table 2:** **Significant KEGG pathways for G2**.

Pathway	Overlapping genes	*p*-value	*q*-value
Cholinergic synapse	*CREB3L2*; *GNG4*; *CACNA1C*; *ADCY9*; *CHRNA7*	0.002	0.015
Hypertrophic cardiomyopathy (HCM)	*CACNA1C*; *ITGB6*; *ACE*; *CACNB4*	0.004	0.015
GABAergic synapse	*GABRB1*; *GNG4*; *CACNA1C*; *ADCY9*	0.005	0.015
Insulin secretion	*CREB3L2*; *CACNA1C*; *ADCY9*; *ADCYAP1*	0.005	0.015
Dilated cardiomyopathy	*CACNA1C*; *ITGB6*; *ADCY9*; *CACNB4*	0.006	0.015
Circadian entrainment	*GRIN2B*; *GNG4*; *CACNA1C*; *ADCY9*	0.007	0.015
Rap1 signaling pathway	*TEK*; *PDGFD*; *ADCY9*; *DOCK4*; *GRIN2B*; *SIPA1L1*	0.007	0.015
Adrenergic signaling in cardiomyocytes	*CREB3L2*; *ADRB2*; *CACNA1C*; *ADCY9*; *CACNB4*	0.008	0.015
Retrograde endocannabinoid signaling	*GABRB1*; *GNG4*; *CACNA1C*; *ADCY9*	0.008	0.015
Endocytosis	*VPS37C*; *NTRK1*; *AGAP1*; *ADRB2*; *TFRC*; *HSPA1L*	0.008	0.015
Glutamatergic synapse	*GRIN2B*; *GNG4*; *CACNA1C*; *ADCY9*	0.011	0.020
Neuroactive ligand–receptor interaction	*NTSR2*; *GRIN2B*; *ADRB2*; *CHRNA7*; *GABRB1*; *HCRTR2*	0.014	0.023
Dopaminergic synapse	*CREB3L2*; *GRIN2B*; *GNG4*; *CACNA1C*	0.019	0.027
cAMP signaling pathway	*CREB3L2*; *GRIN2B*; *ADRB2*; *CACNA1C*; *ADCY9*	0.020	0.027
Ras signaling pathway	*GRIN2B*; *GNG4*; *TEK*; *PDGFD*; *RASGRF2*	0.034	0.043

*KEGG, Kyoto Encyclopedia of Genes and Genomes; GABA, gamma-aminobutyric acid; Rap1, Ras-related protein 1; cAMP, Cyclic adenosine monophosphate*.

## Discussion

By using multivariate Para-ICA to link specific GM volume measurements often found to be abnormal in ADHD with several genetic pathways, we showed that specific ADHD-relevant GM volume deficits can be linked to constellations of genes implicated in different physiological pathways. The importance of this should not be overlooked, as most prior studies have been able to link single or very small numbers of specific genotypes to the broad ADHD diagnostic phenotype, not comparatively large genotype, aggregates to specific neurobiological features known to be abnormal in the disorder.

We discuss two of the three phenotype components (S1 and S2; see Figures 3B and 4B) that showed ADHD versus non-ADHD differences (Figure 2) and whose correlation with cognitive performance or parent-reported clinical impairments showed that genetic factors explain a noteworthy portion of specific GM volume phenotypic variability relevant to ADHD. The most prominent brain regions in S1 and S2 phenotype components were cingulate and basal ganglia, respectively. These were the only regions to emerge as consistently abnormal in the most recent ADHD VBM meta-analysis (40). Along with insular cortex (the other most prominent regions within S1), the anterior cingulate forms a functionally integrated neural circuit (41, 42) reliably linked to attention, conflict resolution, performance monitoring, and switching among cognitive states (14, 43, 44) and whose reduced GM volume has been linked to abnormal attention modulation and inhibitory capacity in ADHD (15). Putamen relevance to ADHD is shown by frequent reports of volume abnormality (13, 45), ADHD-like behavior following lesions (46), and correlation between ADHD symptoms and functional abnormalities (47). S1 and S2 also contained parietal and cerebellar regions, which also are implicated in ADHD (13, 14, 40, 43, 45, 48). Taken collectively, Para-ICA identified the specific ADHD GM volume abnormalities in ADHD that would be predicted by proposals for multi-systemic neural impairment in ADHD in fronto-striatal (49, 50) and fronto-cerebellar (11, 49) neural circuits (51).

We show for the first time that these specific brain phenotypes had genetic correlates – the aggregate of genes in the G2 IC. Although the single genes we found should be noted for future ADHD genetic experiments, interpretation of single genes would be premature pending GWAS replication. However, pathway analysis suggests several possible biological/molecular influences on brain volume that could potentially mediate disease risk in ADHD – some familiar, others more novel. We hypothesized that neurodevelopmental biological pathways logically would predict some aspects of ADHD brain structure abnormality. We found the brain phenotype components were related to differences in expression of a neuroactive ligand–receptor interaction pathway, which has been linked to neurodevelopment and is associated with various psychiatric disorders (52, 53), and also to a specific signal transduction pathway, i.e., *Rap1* (ras-related protein 1), whose pathway involves cell adhesion and regulation of mitogen-activated protein kinase that are important for neural development (54, 55) in the form of neocortical neuronal migration and lamination (56). *Rap1* is a key mediator of calcium regulation of CREB-dependent transcription and dendritic development that are responsible for neural development (57, 58). *Rap1* signaling pathways are plausibly implicated in cortical and subcortical neurodevelopment that may be relevant to ADHD. Future studies might test if these pathways are related to brain development delays, where frontal, temporal, and basal ganglia brain regions lag behind non-ADHD peers by an average of 3 years (59).

Another study prediction was that we would identify specific DA- and norepinephrine (NE)-related genotypes that would be associated with the ADHD brain structure abnormalities. The genes in the G1 component implicate a pathway involved with DA-related synaptic structure and function (see Table 2), some of which directly involve DA neurotransmission *DRD2* (D2 receptor) (60) and *DDC* – a general non-specific precursor of DA and 5HT synthesis (61). There are long-standing theories of dysfunctional catecholaminergic function in ADHD mesocortical and mesolimbic DA systems (62, 63). ADHD findings include abnormally increased DA receptor density in those specific fronto-striatal regions that can be adequately assessed using available positron emission tomography ligands (64), and evidence that psychostimulant medications work in large part because of their ability to increase synaptic DA levels by blocking the DA transporter (65). Although the precise mechanisms underlying DA dysfunction in ADHD are incompletely understood, much recent inquiry has involved the ways in which DA and its NE exert neuromodulatory effects on widespread brain regions important in ADHD and other developmental psychiatric disorders (66, 67).

Many of the same genes identified by Para-ICA related to DA synapses also have been linked to KEGG pathways for glutamate, GABA, and cholinergic synapses, as well as retrograde endocannabinoid signaling. Previous research has implicated all these neurotransmitter systems in ADHD (68), and the current results should serve to bolster interest in exploring the role of these other systems in the pathophysiology of ADHD. In particular, the glutamatergic system is implicated by several lines of evidence (69). Not only has previous genome-wide analysis identified possible association with genes involved in synaptic adhesion, glutamate receptors, and intracellular signaling pathways in ADHD but also there are magnetic resonance spectroscopy (MRS)-detected fronto-striatal glutamate metabolite abnormalities in ADHD (70, 71). Several mouse models (72, 73) highlight the interactive impact of glutamatergic genotypes on dopamine-related brain function and ADHD behaviors like hyperactivity (74). Glutamate/GABA interactions have long been the focus of inquiry with respect to regional cellular excitation/inhibition balance, and recent fMRI study also has linked them to task-induced activity, intrinsic activity within the brain’s “default mode,” and functional integration of widely distributed neural networks (75). Such interactions at the synaptic level could be related to ADHD pathophysiology. Less prevalent in ADHD prior research is the endocannabinoid system. CB_1_ and CB_2_ receptors are expressed most strongly in the basal ganglia that formed parts of the S2 brain phenotype component (76). This system is involved in medial prefrontal cortical (mPFC) dopamine release through changes in GABA inhibitory synapse (77), and there are numerous instances of interaction between these two neurotransmitter systems (78, 79), including those in rodents ADHD models (80). Finally, the retrograde endocannabinoid signaling pathway has been implicated in impulsivity (20). The GABAergic system contributes to impulsivity-related brain function and behavior (81), e.g., rodent studies suggest that impaired synaptic integration of DA and glutamatergic afferents targeting GABAergic medium-spiny neurons are associated with impulsivity (82). A recent MRS study revealed reduced GABA concentration in ADHD patients (83). A previous rodent study also has implicated GABA in ADHD by showing that the loss of GABAergic interneurons in cortex was associated with motor hyperactivity (84). In addition, the perturbation of synaptic connectivity of GABAergic interneurons was found to produce abnormal behaviors relevant to various neuropsychiatric disorders (85). Support for a role of acetylcholine systems is found in studies that found choline-containing compounds are altered across various brain regions in ADHD patients (68). Moreover, acetylcholine and dopamine interactions within the striatum modulate dopamine-related neuronal activity that signals motivational salience (86). Finally, Tourette syndrome – which is a common comorbid condition with ADHD (87) – has been linked to reduced basal ganglia volume (88) and deficits of cholinergic interneuron in dorsolateral striatum (89).

The implications of the pathways related to circadian entrainment and cAMP signaling are less obviously interpreted. A substantial literature has examined the relationship between sleep disturbance and ADHD, e.g., delayed circadian rhythmicity in ADHD (90, 91), links between impulsivity and circadian entrainment (20), or the role of DA signaling (92). However, specific mechanisms have not been identified that have etiological relevance to ADHD. This pathway might be added to proposed research agenda aimed at understanding the overlap between sleep-related brain physiology and known ADHD deficits [e.g., neurotransmitters involved in both sleep and attention regulation, or phenotypic similarities between the deleterious effects of sleep deprivation and ADHD (93)]. cAMP impairment has been suggested by a rat model of ADHD (94) and findings of cAMP accumulation and reduced cAMP sensitivity during adolescence that might be a mechanism underlying ADHD symptom expression changes throughout adolescence (95). Additionally, a study has reported cAMP-related protein kinase to be responsible for dopamine transporter cell-surface redistribution that is involved in ADHD (96). A cogent neurobiological model of cAMP involvement in ADHD is needed to guide future research.

The multivariate nature of the Para-ICA results is not easily reducible to univariate interpretations about specific gene function, or about single gene contributions directly to ADHD. In other words, individual gene mapped to specific pathways does not prove that each gene has a direct casual role in ADHD risk. Therefore, we focused our discussion on pathways instead of individual genes. In a similar vein, the results should not be viewed as definitive until replication, despite the rigor of prior Para-ICA method validation and the statistical methods we used to assess reliability/consistency. Instead, they are intended to accelerate thinking about both familiar and novel pathways and their etiological role in ADHD. Overall, our pathway results have some similarities with those from previous pathway enrichment analyses in ADHD that were conducted to identify possible biological mechanisms when GWAS-level statistical evidence was not obtained. Both our studies and others have found evidence for both synaptic function and plasticity (23, 24, 97), particularly in the glutamate system, or processes involved in neural development (5, 22, 98, 99). Both we and Alemany et al. (24) found evidence specifically for the KEGG neuroactive ligand–receptor interaction pathway, raising the possibility that this pathway might be related to both cognitive performance based associations found in that study might be related to the brain structure correlates found in this one (i.e., our brain phenotype S2 component was linked to CPT-II performance indices in *post hoc* analyses). However, our results join other pathways implicated in ADHD – included directed neurite growth (23, 98), rRNA processing, CNS development (99), nucleocytoplasmic transport, mitochrondrial function (100), protein ubiquitination (5), apoptosis, oxidation reduction, and immune response (101). A reason we did not find these others might simply be because our results are specific to the structural GM volume abnormalities found in ADHD – an association that might be closer to etiological factors than behavior or clinical impairment. The unexpected findings of pathways related to transmission and signaling in heart muscle [dilated and hypertrophic cardiomyopathy (HCM) and adrenergic signaling in cardiomyocytes], insulin secretion, estrogen signaling, and endocytosis likely occurred because many of the genes in those systems overlapped with those discussed above, including *CACNA1C, CACNB4, ITGB6, ACE, ADCY9, ADRB2, AGAP1, ARRB1, CREB3L2, FKBP5, HSPA1L, TFRC*, and *VPS37C*. Numerous studies have shown these genes are expressed in brain, responsible for neurogenesis (102–105), or associated with neuropsychiatric disorders (106–109).

### Limitations

Study limitations include factors like early infant history, nutrition, medication exposure (40, 59), or similar ADHD-relevant experiences that might affect neurodevelopment, synaptic pruning, etc., were not measured or controlled as is often done in genetic inquiry. Such factors will become more important in any replication study, or in studies that examine specific pathophysiological mechanisms suggested by this study. Also our pathway analysis was limited by the extent of current knowledge on gene products and function (110), which will no doubt improve rapidly. Further, using an exome array, we were able to include genome-wide exomic information; however, this was not a genome-tagging array and could not examine non-exomic variation directly at all. Finally, as mentioned consistently above, our study goal was not to identify single gene associations with the ADHD behavioral phenotype, as in GWAS. Instead, our goal was to look within a collection of ADHD-relevant genes to determine if any relationships could be found with specific aspects of GM volume already known to be abnormal in ADHD. While we hope the gene–brain structure relationships we found will prompt future study of both the gene pathways and the individual genes in ADHD, this study is not intended to be a comprehensive inquiry of all possible ADHD-related genes. Rather, the point is to accelerate identification of novel intermediate endophenotypes to be explored in future research. The SNPs identified in current study should be further studied to determine whether any of the specific genes show a conventional genetic association with either the ADHD diagnostic phenotype, specific to the potential brain structure intermediate phenotype we described, or if the gene–brain relationships hold only the implicated biological pathways.

## Author Contributions

Mr. SK was involved in data preprocessing, data analysis, and preparation of manuscript. Drs. GP, VC, and JG was involved in design of study and preparation of manuscript. Dr. JL was involved in data analysis and preparation of manuscript. Miss. KB was involved in data collection. Dr. MS was involved in design of study, data analysis, and preparation of manuscript.

## Conflict of Interest Statement

The authors declare that the research was conducted in the absence of any commercial or financial relationships that could be construed as a potential conflict of interest.

## Footnotes

^1^http://dbm.neuro.uni-jena.de/vbm8/VBM8-Manual.pdf

^2^http://mialab.mrn.org/software/fit/

^3^http://cpdb.molgen.mpg.de/
