# Supplementary material for: Multivariate Imaging Genetics Study of MRI Gray Matter Volume and SNPs Reveals Biological Pathways Correlated with Brain Structural Differences in Attention Deficit Hyperactivity Disorder
Source: Front Psychiatry. 2016 Jul 25;7:128. doi: 10.3389/fpsyt.2016.00128 (PMC4959119; doi:10.3389/fpsyt.2016.00128)
Supplement: Supplementary file 1 [file Table_1.PDF]

Supplementary Table 1. Demographic and Clinical Information

| | | ADHD | | HC | |
|---|---|---|---|---|---|
| | | Male | Female | Male | Female |
| Number of participants (%) | | 51 (25.7) | 12 (6.06) | 68 (34.31) | 67 (33.8) |
| Average age (standard deviation) | | 14.8(1.7) | 15.4(1.7) | 16.9(2.5) | 17.2(3.6) |
| Handedness | Left | 7 | 0 | 0 | 3 |
| | Right | 44 | 12 | 67 | 66 |
| | Ambidextrous | 0 | 0 | 1 | 1 |
| Race | Caucasian-American | 36 | 9 | 60 | 52 |
| | African-American | 7 | 0 | 7 | 12 |
| | Mixed/Other | 8 | 3 | 1 | 3 |
| †Brown | Attention | 20.8 (15.5) | 15.9 (5.3) | | |
| | Effort | 20.8 (14.9) | 13.8 (3.8) | | |
| | Affect | 13.6 (16.7) | 7.4 (3.8) | | |
| | Working memory | 14.3 (15.0) | 9.0 (2.7) | | |
| ‡CPT II | Omissions | 57.5 (20.5) | 54.5 (14.4) | | |
| | Commissions | 51.6 (8.7) | 58.8 (13.5) | | |
| | Variability | 53.4 (11.06) | 52.6 (14.6) | | |
| | Detectability | 53.2 (9.2) | 55.3 (14.7) | | |
| | Hit RT | 48.07 (11.9) | 45.3 (9.7) | | |
| | Hit RT SE | 53.3 (10.2) | 53.5 (13.7) | | |
| | Hit RT by Block | 50.8 (11.2) | 51.03 (7.3) | | |
| | Hit SE Block | 51.9 (11.3) | 51.9 (11.3) | | |
| | Hit RT by ISI | 53.6 (9.4) | 53.7 (13.9) | | |
| | HIT SE by ISI | 52.8 (8.7) | 53.1 (13.5) | | |
| *Intelligence Quotient | | 102 (13) | 104 (12.8) | 111.5 (10.1) | 109.9(12.3) |
| **WRAT Reading Score | | 101.5(12.1) | 101.2(14.2) | 109.5(8.8) | 105.5(8.6) |

ADHD: Attention-Deficit Hyperactivity Disorder; HC: Healthy controls; CPT: Continuous Performance Test; RT: Reaction Time; SE: Standard error; WRAT: Wide Range Achievement Test. †Brown attention deficit disorder scales were missing from 12 male participants. ‡CPT scores were missing from 12 male and 1 female participants. * Intelligence Quotient score were missing from 11 Male ADHD patients; 4 Male and 9 Female HCs. **WRAT reading scores were missing from 2 female HCs. Average scores from Brown attention deficit disorder scales and CPT II scores are listed along with their standard deviation in parenthesis. The DSM-IV diagnoses for ADHD group revealed 13 patients had oppositional defiant disorder, 7 patients had conduct disorder, 2 patients with past major depressive episodes, 1 patient with current dysthymia, 2 patients with adjustment disorder and 1 patient each with separation anxiety, post-traumatic stress disorder, cannabis abuse and past substance abuse.
