# Supplementary material for: Multivariate Imaging Genetics Study of MRI Gray Matter Volume and SNPs Reveals Biological Pathways Correlated with Brain Structural Differences in Attention Deficit Hyperactivity Disorder
Source: Front Psychiatry. 2016 Jul 25;7:128. doi: 10.3389/fpsyt.2016.00128 (PMC4959119; doi:10.3389/fpsyt.2016.00128)
Supplement: Supplementary file 2 [file Table_2.PDF]

Supplementary Table 2. List of SNPs and associated genes after SNPs preprocessing

SNPs	Chromosome	Location	allele1	allele2	Gene
exm4951	1	1900186	C	T	KIAA1751
rs2376805	1	1956362	C	T	GABRD
rs16824627	1	1962633	C	T	GABRD
exm2268641	1	2327815	C	T	RER1
exm6264	1	2526746	G	A	MMEL1
exm2268642	1	2539006	C	T	MMEL1
exm6351_ver3	1	2541269	G	A	MMEL1
exm-rs3890745	1	2553624	G	A	MMEL1
exm6548	1	3328358	T	C	PRDM16
exm7575	1	3679783	C	T	CCDC27
exm7844	1	3753136	T	A	CEP104
exm9590	1	6531589	T	C	PLEKHG5
exm10282_ver2	1	6698390	T	C	DNAJC11
exm-rs970973	1	7202190	C	T	CAMTA1
exm2260823	1	9068386	A	G	SLC2A7
exm11865	1	9165685	A	G	GPR157
exm12190	1	9640253	A	C	SLC25A33
rs12128766	1	9937041	T	C	CTNNBIP1
exm13835	1	11014118	T	C	C1orf127
exm13854	1	11015165	G	A	C1orf127
exm-rs17375901	1	11852516	T	C	MTHFR
exm15710	1	12010469	T	G	PLOD1
exm18549	1	13802373	A	G	LRRC38
exm18606	1	13940864	C	G	PDPN
exm19023	1	15438990	A	G	KAZN
exm-rs2901964	1	15792426	G	C	CELA2A
rs5257	1	16373124	A	G	CLCNKB
exm21233	1	16380196	T	C	CLCNKB
exm-rs9442235_ver3	1	16393357	T	G	FAM131C
exm21359	1	16456763	T	C	EPHA2
exm21565	1	16532498	A	G	ARHGEF19
exm21810	1	16730308	T	G	SPATA21
exm21826	1	16731510	G	C	SPATA21
exm-rs2284746_ver2	1	17306675	C	G	MFAP2
exm23022	1	17326767	T	C	ATP13A2
exm23450	1	17593316	A	G	PADI3
exm2264991_ver2	1	18563889	A	G	IGSF21

exm2268659	1	20056631	C	T	TMCO4
exm26437	1	20140973	T	G	RNF186
exm26817	1	20645086	A	T	VWA5B1
exm27075	1	20960385	T	A	PINK1
exm2264808	1	20978058	G	A	PINK1-AS
exm27277	1	20992819	G	C	KIF17
exm28840	1	22169325	T	C	HSPG2
exm-rs1077514	1	23766233	G	A	ASAP3
exm31057	1	23847464	A	C	E2F2
exm31060	1	23847529	T	C	E2F2
exm31621	1	24201162	A	G	CNR2
exm32214_ver2	1	24658063	G	C	GRHL3
exm32374	1	24710405	G	A	STPG1
exm32810	1	25570081	C	T	C1orf63
exm1778926	1	25629943	C	A	RHD
exm33280	1	26131654	G	A	SEPN1
exm33331	1	26140573	C	A	SEPN1
exm2273320	1	26450009	T	C	PDIK1L
exm34778	1	26691174	A	C	ZNF683
exm34794_ver2	1	26691310	C	G	ZNF683
exm35769	1	27278553	A	G	C1orf172
exm37273	1	28282292	C	T	SMPDL3B
exm2264815	1	31661921	A	G	NKAIN1
exm2263858	1	31681614	A	C	NKAIN1
exm39723	1	32164206	T	G	COL16A1
exm40118	1	32280369	A	G	SPOCD1
exm45352	1	36888462	G	A	OSCP1
exm45360	1	36893968	G	A	OSCP1
exm46315	1	38186189	T	C	EPHA10
exm48342	1	40098328	T	C	HEYL
exm49418	1	40928731	A	G	ZFP69B
exm49449	1	40960619	A	G	ZFP69
exm49502	1	40980559	A	G	EXO5
exm49512	1	40980731	T	G	EXO5
exm51160	1	43296130	T	C	ERMAP
exm53353	1	44447413	C	G	B4GALT2
exm56819	1	47024308	T	C	MKNK1
exm57130	1	47280884	T	C	CYP4B1
exm57602	1	47726087	C	T	STIL

exm57998	1	48825355	A	G	SPATA6
exm-rs3127553	1	49438005	G	A	AGBL4
exm59762	1	53320274	G	T	ZYG11A
exm59946	1	53532535	C	G	PODN
exm59950	1	53535478	A	G	PODN
exm60394	1	53712727	T	C	LRP8
exm60404	1	53716416	A	G	LRP8
exm61537	1	55014013	T	C	ACOT11
exm63130	1	57216747	A	G	C1orf168
exm63139	1	57219539	A	C	C1orf168
exm2252414	1	60152685	A	G	FGGY
exm2268895	1	60211466	T	C	FGGY
exm66924	1	67288045	T	C	WDR78
exm68136	1	70881670	T	C	CTH
rs977214	1	71426260	G	A	PTGER3
exm69632	1	77685042	C	T	PIGK
exm70323	1	79093818	G	A	IFI44L
exm2268719	1	86457334	C	T	COL24A1
exm72104	1	86591837	G	A	COL24A1
exm72333	1	86909582	A	G	CLCA2
exm72516	1	87012929	T	C	CLCA4
exm73938	1	90179532	G	A	LRRC8C
exm-rs12745968	1	93401837	G	A	FAM69A
exm2273328	1	95363001	C	T	CNN3
exm76967	1	97770920	T	C	DPYD
exm77579	1	100358103	T	C	AGL
exm2249471	1	102389733	T	C	OLFM3
exm2263862	1	107725743	C	A	NTNG1
exm2268737	1	108737586	T	C	SLC25A24
exm2268924	1	109723090	T	C	KIAA1324
exm82825_ver3	1	110921715	G	T	SLC16A4
exm84327	1	112999000	A	G	CTTNBP2NL
exm86002	1	114948281	A	G	TRIM33
rs6537825	1	114948281	A	G	TRIM33
exm86197	1	115168097	C	T	DENND2C
exm86221	1	115168600	T	A	DENND2C
exm86506	1	115417175	G	A	SYCP1
exm86587	1	115537367	C	A	SYCP1
exm2249734	1	118504265	T	G	SPAG17

exm2259741	1	118522545	A	G	SPAG17
exm88431	1	118535211	C	T	SPAG17
exm-rs11249433	1	121280613	C	T	EMBP1
exm90704	1	145015877	T	G	PDE4DIP
exm90719	1	145021126	A	G	PDE4DIP
exm94752_ver3	1	150526270	T	C	ADAMTSL4
exm95933	1	151108137	T	G	SEMA6C
exm96391	1	151259543	A	G	ZNF687
exm96713	1	151337703	T	C	SELENBP1
exm96822	1	151374025	C	T	PSMB4
exm102076	1	153277423	T	C	PGLYRP3
rs3811450	1	154551032	T	C	CHRNB2
exm105716_ver3	1	155057650	C	T	EFNA3
exm2273337	1	155106697	A	G	EFNA1
exm106411	1	155231927	T	C	SCAMP3
exm110358	1	156784982	T	C	NTRK1
exm111298_ver2	1	157062739	G	C	ETV3L
exm111320	1	157068533	A	G	ETV3L
exm112226	1	158224904	T	C	CD1A
exm112233	1	158225019	G	C	CD1A
exm112747	1	158450314	A	G	OR10R2
exm112752	1	158450382	T	C	OR10R2
exm112919	1	158549492	C	T	OR10X1
exm112921	1	158549511	A	G	OR10X1
exm112975	1	158577109	C	A	OR10Z1
exm113004	1	158584091	A	G	SPTA1
exm113113	1	158607935	G	T	SPTA1
exm113133	1	158612236	A	G	SPTA1
exm-rs857721	1	158612548	A	T	SPTA1
exm113507	1	158687451	T	C	OR6K3
rs1130864	1	159683091	T	C	CRP
exm116933_ver2	1	161019040	G	C	ARHGAP30
exm-rs11265582	1	161255333	T	C	PCP4L1
exm118403	1	161693199	T	C	FCRLB
exm118446	1	161697072	C	G	FCRLB
exm118696	1	161969986	A	G	OLFML2B
exm2264911	1	162168116	G	A	NOS1AP
exm-rs4657178	1	162210610	T	C	NOS1AP
exm2259837	1	164733504	G	A	PBX1

exm119597	1	165532948	T	C	LRRC52
exm120343	1	167097567	A	G	DUSP27
exm120788	1	167825449	C	T	ADCY10
exm120972	1	168013850	C	T	DCAF6
rs9332624	1	169497926	C	A	F5
exm121844	1	169510890	C	G	F5
exm121943	1	169513583	T	G	F5
exm2250216	1	169513583	T	G	F5
rs6037	1	169513583	A	C	F5
exm121977	1	169521853	G	A	F5
exm2259849	1	169527856	C	T	F5
rs2213869	1	169536167	C	T	F5
rs6691048	1	169542135	T	C	F5
exm122222	1	169676486	G	A	SELL
exm123042	1	171076966	A	G	FMO3
exm-rs17346452	1	172053287	C	T	DNM3
exm-rs10913469	1	177913519	C	T	SEC16B
exm126979	1	179112145	G	C	ABL2
exm127154	1	179414105	C	A	AXDND1
exm-rs12047808	1	179469314	G	A	AXDND1
exm127303	1	179562740	C	G	TDRD5
exm127384	1	179631242	A	G	TDRD5
exm2264926	1	180226930	G	A	LHX4
exm128992	1	182026813	C	G	ZNF648
exm129002	1	182027022	T	C	ZNF648
exm129312	1	182569626	T	C	RGS16
exm2265063_ver4	1	182854200	A	G	DHX9
exm129667	1	183085755	A	G	LAMC1
exm129731	1	183094547	T	C	LAMC1
exm129778	1	183099560	A	G	LAMC1
exm2264930	1	183106739	G	A	LAMC1
exm2268957	1	184410702	T	C	C1orf21
exm131295	1	185958737	G	A	HMCN1
exm132839	1	192628623	C	G	RGS13
exm135332	1	200534255	A	T	KIF14
exm2250472	1	200959302	A	G	KIF21B
exm136224	1	201016296	A	G	CACNA1S
exm136890	1	201184878	A	C	IGFN1
exm137330	1	201355761	G	A	LAD1

exm137390	1	201358304	A	C	LAD1
exm138344	1	202128601	T	C	PTPN7
exm2232286	1	203309752	C	T	FMOD
exm2250505	1	205318321	T	C	KLHDC8A
rs3024496	1	206941864	C	T	IL10
rs1878672	1	206943713	G	C	IL10
rs3024491	1	206945046	T	G	IL10
rs2222202	1	206945381	T	C	IL10
exm143879	1	207071206	C	T	IL24
exm143990	1	207106478	A	G	PIGR
exm2250513	1	207110936	T	C	PIGR
exm144091	1	207131878	C	T	FCAMR
exm144740	1	207653364	G	A	CR2
exm144917	1	207782856	G	A	CR1
exm145016	1	207851611	G	A	CR1L
exm145219	1	208073205	A	C	CD34
exm145290	1	208212224	T	C	PLXNA2
exm145503	1	208390903	T	G	PLXNA2
exm2268829	1	215862696	T	C	USH2A
exm149015	1	215990479	T	C	USH2A
exm149022	1	216011361	C	T	USH2A
exm2260011	1	221898783	C	T	DUSP10
exm151013	1	222802376	G	A	MIA3
exm151047	1	222803204	G	A	MIA3
exm151275	1	222923351	A	G	FAM177B
exm151525	1	223285200	A	G	TLR5
exm151555_ver2	1	223285833	T	G	TLR5
exm151810	1	223813586	G	T	CAPN8
exm151926	1	223947081	G	A	CAPN2
exm152266	1	224482084	T	C	NVL
exm154612	1	227935762	A	G	SNAP47
exm155754	1	228469801	C	G	OBSCN
exm156064_ver3	1	228496066	T	G	OBSCN
exm156457	1	228548197	A	G	OBSCN
exm157383	1	229654053	C	T	ABCB10
exm2250857	1	230469505	A	G	PGBD5
exm157996	1	230810756	A	T	COG2
exm158224	1	230914729	C	A	CAPN9
exm159152	1	232561520	A	G	SIPA1L2

exm159365	1	232940953	C	T	MAP10
exm159388	1	232941215	G	A	MAP10
exm159776	1	233394248	C	T	PCNXL2
rs7512377	1	234276639	T	C	SLC35F3
exm160224	1	234565176	T	C	TARBP1
exm160265	1	234582720	T	C	TARBP1
exm-rs291353	1	235720898	G	A	GNG4
exm-rs7520258	1	236313127	C	T	GPR137B
exm162802	1	237841390	G	A	RYR2
exm164070	1	242035438	A	G	EXO1
exm2263856	1	246182293	C	A	SMYD3
exm166600	1	247752367	G	A	OR2G2
exm166656	1	247769752	G	A	OR2G3
exm166989	1	248039294	A	G	TRIM58
exm167043	1	248059423	T	C	OR2W3
exm167892	1	248458760	A	C	OR2T12
exm168894	1	248844959	C	T	OR14I1
exm168903	1	248845097	C	T	OR14I1
exm-rs11542478	2	38938	C	A	FAM110C
rs10204613	2	2226513	A	C	MYT1L
exm2265110	2	3384825	G	A	TRAPPC12
exm171024	2	7137067	A	G	RNF144A
rs12612207	2	11807216	T	C	NTSR2
rs4669765	2	11808654	G	T	NTSR2
rs4669767	2	11808864	T	C	NTSR2
rs7567183	2	11811286	C	T	NTSR2
exm-rs2710684	2	17814030	T	C	VSNL1
exm175272_ver2	2	20824498	C	T	HS1BP3
exm-rs4971516	2	20903015	C	T	C2orf43
exm175490	2	21225753	T	C	APOB
rs737665	2	23891433	G	A	KLHL29
exm176529	2	24262060	A	G	C2orf44
exm176581	2	24300563	C	T	TP53I3
exm176660	2	24358001	C	G	FAM228B
exm2269259	2	25116977	T	C	ADCY3
exm177349	2	25141538	A	G	ADCY3
exm-rs4665736	2	25187599	T	C	DNAJC27
exm-rs6733301	2	25276284	A	G	EFR3B
exm-rs12233132	2	25328703	T	C	EFR3B

rs12233134	2	25329016	T	C	EFR3B
rs12613265	2	25329898	C	T	EFR3B
rs563590	2	25331172	G	A	EFR3B
exm-rs11684202	2	25887558	G	A	DTNB
exm182853	2	28854958	T	C	PLB1
exm2255318	2	31178818	T	C	GALNT14
exm-rs6714546	2	33361425	A	G	LTBP1
exm187606	2	38224611	G	A	RMDN2
exm188345	2	39241107	A	G	SOS1
exm2022294_ver2	2	39431687	A	G	CDKL4
exm-rs13429458	2	43638838	C	A	THADA
exm190428	2	44104925	T	C	ABCG8
exm191160	2	45640334	C	T	SRBD1
rs12466402	2	46174037	G	T	PRKCE
exm-rs815815	2	47399064	G	A	CALM2
exm192858	2	48807796	C	A	STON1,STON1-GTF2A1L
exm192886	2	48808152	C	G	STON1,STON1-GTF2A1L
exm192958	2	48809593	T	G	STON1,STON1-GTF2A1L
exm193220	2	49191041	C	T	FSHR
exm-rs2268363	2	49201328	C	T	FSHR
exm2263892	2	50511020	A	C	NRXN1
exm2261278_ver2	2	50565462	C	T	NRXN1
exm193535	2	53955850	T	C	ASB3,GPR75-ASB3
exm193545	2	53955982	A	T	ASB3,GPR75-ASB3
exm193569	2	53992622	C	G	ASB3,GPR75-ASB3
exm193663	2	54035508	C	G	ERLEC1
exm194032_ver2	2	54482553	A	G	TSPYL6
exm194749	2	55254165	A	T	RTN4
exm-rs3791679	2	56096892	C	T	EFEMP1
exm-rs3791675	2	56111309	A	G	EFEMP1
exm2255420	2	56144930	C	T	EFEMP1
exm2261292	2	61184651	A	G	PUS10
exm196707	2	61575308	A	G	USP34
exm-rs7604693	2	64349202	C	A	PELI1
exm2255454	2	66665146	T	C	MEIS1
exm199007	2	69177269	A	C	GKN2
exm201643	2	72361960	G	A	CYP26B1
exm202046	2	73429808	C	G	NOTO

exm202286	2	73491644	T	C	FBXO41
exm203446	2	74177777	G	A	DGUOK
exm204519	2	74692191	T	C	MOGS
exm2269087	2	77589901	T	C	LRRTM4
exm206493	2	84846930	G	A	DNAH6
exm206672	2	85051122	A	G	TRABD2A
rs6732834	2	85505602	T	C	TCF7L1
exm206835	2	85549868	A	G	TGOLN2
exm207656	2	85893741	G	A	SFTPB
exm209227	2	88874891	C	A	EIF2AK3
exm209282	2	88895123	T	C	EIF2AK3
exm-rs7571971	2	88895351	T	C	EIF2AK3
exm209289	2	88913273	C	G	EIF2AK3
rs3813662	2	96779695	G	T	ADRA2B
exm213001	2	96993757	T	C	ITPRIPL1
exm213087	2	97030247	C	T	NCAPH
exm214567	2	98275354	A	G	ACTR1B
exm214966	2	98779439	T	G	VWA3B
exm215008_ver3	2	98844674	C	G	VWA3B
exm215101	2	98928429	G	C	VWA3B
rs10175560	2	99465418	T	C	KIAA1211L
exm216147	2	100055158	C	T	REV1
exm2254420	2	100686486	T	C	AFF3
exm-rs9653442	2	100825367	C	T	LINC01104
exm-rs10865035	2	100835734	G	A	LINC01104
exm2269300	2	101449302	T	G	NPAS2
rs356652	2	101540415	C	A	NPAS2
rs17717414	2	101546550	G	A	NPAS2
rs3820787	2	101566234	A	G	NPAS2
exm216700	2	101591304	A	G	NPAS2
exm216720	2	101594191	T	C	NPAS2
rs11123857	2	101603812	G	A	NPAS2
exm216807	2	101624471	T	C	TBC1D8
exm216825	2	101627925	T	C	TBC1D8
exm-rs3739014	2	101638888	A	G	TBC1D8
rs10185855	2	101642260	G	A	TBC1D8
exm216925	2	101656726	T	C	TBC1D8
rs2282743	2	102632201	T	C	IL1R2
rs3218911	2	102633347	G	A	IL1R2

rs2072481	2	102635931	A	G	IL1R2
exm218507	2	105961788	T	C	C2orf49
exm2269113	2	106765133	T	G	UXS1
exm2265341	2	111752416	A	G	ACOXL
exm-rs1158867	2	128177377	C	T	PROC
exm225669	2	128388862	C	G	MYO7B
exm225759	2	128394955	G	A	MYO7B
exm2261037	2	131512850	T	C	AMER3
exm228878_ver3	2	133541107	A	T	NCKAP5
exm228945	2	133542574	T	C	NCKAP5
exm230080_ver2	2	136590746	T	C	LCT
exm2254628	2	141707868	T	G	LRP1B
rs300373	2	141976620	C	T	LRP1B
exm2269145	2	143743012	G	T	KYNU
exm231469	2	144899559	C	A	GTDC1
exm2269154	2	150000597	T	C	LYPD6B
exm233200	2	152506811	T	C	NEB
exm2265219	2	152859992	G	A	CACNB4
exm-rs707040	2	155226677	C	T	GALNT13
exm2269338	2	158667217	T	C	ACVR1
exm234671	2	159201754	C	T	CCDC148
exm234867_ver4	2	159536990	T	A	PKP4
exm234920	2	159954175	T	C	TANC1
exm-rs1035055	2	160057141	A	C	TANC1
exm235268	2	160132089	G	T	WDSUB1
exm235476_ver2	2	160294842	G	A	BAZ2B
exm2265363	2	160957846	A	G	ITGB6
exm236936	2	163137983	C	T	IFIH1
exm-rs6759814	2	163392761	C	T	KCNH7
exm238204	2	166781188	A	G	TTC21B
exm238633	2	167142979	T	C	SCN9A
exm239077	2	168102774	T	G	XIRP2
exm-rs2287623	2	169830155	C	T	ABCB11
exm239806	2	169830328	A	G	ABCB11
exm240935	2	170502401	A	G	CCDC173
exm241444	2	171371469	A	G	MYO3B
exm242672	2	174131392	C	T	ZAK
exm242808	2	174820750	C	T	SP3
exm243003	2	175263063	A	G	SCRN3

exm243060	2	175289259	T	G	SCRN3
exm-rs1446575	2	177047801	C	A	HOXD-AS1
exm243991	2	177054769	A	G	HOXD1
exm249346	2	179642665	A	G	TTN
exm249675	2	179732845	A	G	CCDC141
exm249736	2	179914640	A	G	CCDC141
exm250588	2	183584755	A	G	DNAJC10
exm250622	2	183605077	A	C	DNAJC10
exm250706	2	183703336	A	G	FRZB
exm-rs1344706	2	185778428	G	T	ZNF804A
exm253053	2	191161622	T	C	HIBCH
rs2280233	2	191850566	G	A	STAT1
exm253998	2	196673533	A	G	DNAH7
exm254315	2	196825238	A	C	DNAH7
exm254406	2	196877557	C	T	DNAH7
exm-rs1992950	2	200290359	G	A	SATB2
exm255885	2	200828526	A	T	C2orf47
exm256370	2	201534389	G	A	AOX1
exm259304	2	206911228	A	G	INO80D
exm259476	2	207041053	C	T	GPR1
exm260011	2	207615724	T	C	MDH1B
rs7569963	2	208473184	A	G	METTL21A
exm261350	2	210560189	G	A	MAP2
exm264542	2	219028932	A	G	CXCR1
exm264607	2	219029843	C	A	CXCR1
exm-rs7349332	2	219756383	T	C	WNT10A
exm268403	2	220197321	T	C	RESP18
exm271258	2	225670901	C	G	DOCK10
exm2263966	2	225867091	C	A	DOCK10
exm271454	2	226491770	A	C	NYAP2
exm272072	2	228128568	A	G	COL4A3
exm272080	2	228131169	G	A	COL4A3
exm2255066	2	228398422	G	A	AGFG1
exm272449	2	228476253	C	G	C2orf83
exm-rs10191097	2	228776671	T	G	DAW1
exm272649_ver2	2	228855866	C	G	SPHKAP
exm273368	2	231036860	T	C	SP110
exm273628	2	231248261	T	C	SP140L
exm273657	2	231258150	T	C	SP140L

exm273841	2	231406070	A	G	SP100
exm273917	2	231738168	A	G	ITM2C
exm2265285	2	232944860	G	A	DIS3L2
exm-rs11677466	2	232982257	T	A	DIS3L2
exm-rs7571816	2	233077064	G	A	DIS3L2
exm-rs10933436_ver3	2	233998481	A	C	INPP5D
exm276476	2	234229320	G	A	SAG
exm277187	2	234602191	G	A	UGT1A6
exm278253	2	236708166	T	C	AGAP1
exm280025	2	238990388	A	G	SCLY
exm280107	2	239009074	A	G	ESPNL
exm280275	2	239039637	T	C	ESPNL
exm280539	2	239155053	T	C	PER2
rs934945	2	239155053	A	G	PER2
rs880140	2	239155732	T	C	PER2
rs2304669	2	239165663	G	A	PER2
rs2304674	2	239181904	C	T	PER2
rs2304673	2	239185922	C	A	PER2
exm280817	2	239237953	T	G	TRAF3IP1
exm283015	2	241827798	C	T	C2orf54
exm283076	2	241834907	T	C	C2orf54
exm283711	2	242077496	T	C	PASK
exm2269243	2	242110786	T	C	PPP1R7
exm-rs4684585	3	883851	G	A	AC090044.1
exm-rs2619566	3	2624938	C	T	CNTN4
exm2263979	3	2954866	C	A	CNTN4
exm286072	3	3139957	C	T	IL5RA
exm-rs6768750	3	7240675	A	G	GRM7
rs162209	3	7741897	T	C	GRM7
rs2268493	3	8800840	C	T	OXTR
rs4564970	3	8810408	C	G	OXTR
rs2301261	3	8810896	A	G	OXTR
exm288917	3	9959410	A	G	IL17RC
exm289250	3	9991429	A	G	PRRT3
exm289683	3	10219567	A	C	IRAK2
rs3774119	3	10893705	C	T	SLC6A11
rs1881354	3	10958228	A	G	SLC6A11
exm-rs4684746	3	10966588	G	A	SLC6A11
rs1881364	3	10971358	T	C	SLC6A11

rs2601124	3	11035963	T	C	SLC6A1
exm293253	3	14939088	A	G	FGD5
exm294714	3	17349498	C	T	TBC1D5
exm295029	3	19961330	C	T	EFHB
exm295063	3	19975215	A	C	EFHB
exm2265426	3	19975582	A	G	EFHB
exm2255941	3	20109901	G	T	KAT2B
exm295375	3	23848835	C	A	UBE2E1
exm296090	3	27326131	C	T	NEK10
exm-rs4380451	3	32329497	T	C	CMTM8
rs4276227	3	32330686	T	C	CMTM8
exm300488	3	38271881	T	C	OXSR1
exm301689	3	38936134	T	C	SCN11A
exm-rs2286720	3	42448471	A	G	LYZL4
exm304842	3	43074434	T	C	FAM198A
exm305188	3	43647271	G	T	ANO10
exm306946	3	45779136	A	T	SACM1L
exm307040	3	45814094	A	G	SLC6A20
exm307727	3	46449863	A	G	CCRL2
exm307906	3	46501213	C	T	LTF
exm308071	3	46658775	T	C	LOC100132146
exm310131	3	47618825	C	A	CSPG5
exm310138	3	47618953	C	A	CSPG5
exm311976	3	48623124	A	G	COL7A1
exm317688	3	50332697	A	G	HYAL3
exm-rs13088462	3	51071713	C	T	DOCK3
exm320851_ver4	3	52409421	G	A	DNAH1
exm2261511	3	52477866	C	T	SEMA3G
exm2256097_ver2	3	53776678	A	G	CACNA1D
exm324319	3	53892830	C	T	IL17RB
exm325567	3	57335876	C	T	DNAH12
exm325625	3	57401178	C	G	DNAH12
exm325636	3	57414071	G	A	DNAH12
exm325637	3	57414097	A	T	DNAH12
exm325712	3	57448553	T	C	DNAH12
exm2269627	3	58022761	C	T	FLNB
exm2256112	3	58036651	C	T	FLNB
exm326927	3	58552997	A	C	FAM107A
exm326958	3	58625875	A	C	FAM3D

rs7624360	3	60125182	A	T	FHIT
exm2261524	3	62552794	T	C	CADPS
exm328666	3	66433735	A	G	LRIG1
exm330361	3	74334560	T	C	CNTN3
exm2269473	3	77526031	T	G	ROBO2
exm2265596	3	79354187	A	G	ROBO1
exm331585	3	88189341	T	C	ZNF654
exm2273374	3	89530956	A	G	EPHA3
exm331940	3	93768268	G	C	ARL13B
exm332425	3	97806305	C	G	OR5AC2
exm332448	3	97806616	A	G	OR5AC2
exm334875	3	101540387	G	T	NXPE3
exm335284	3	107096547	A	G	CCDC54
exm2269504	3	107805334	T	C	CD47
exm335721	3	108147728	C	T	MYH15
exm336254	3	108639384	C	T	GUCA1C
exm2238321	3	112546399	T	C	CD200R1L
exm337765	3	112642568	C	G	CD200R1
rs9834217	3	113789029	T	C	QTRTD1
exm339341_ver3	3	113804708	G	A	QTRTD1
rs7632503	3	113878583	G	A	DRD3
rs9860828	3	115385682	G	A	GAP43
rs3772933	3	115417773	A	G	GAP43
rs283367	3	115431207	T	C	GAP43
rs13077590	3	115438658	A	G	GAP43
rs10513826	3	116164892	C	T	LSAMP
rs4688043	3	119591248	A	T	GSK3B
exm341384	3	121206428	A	C	POLQ
exm341481	3	121208611	A	G	POLQ
exm341655	3	121304933	A	G	ARGFX
exm342722	3	122003769	G	A	CASR
exm343082	3	122296647	A	G	PARP15
exm343236	3	122419292	G	A	PARP14
exm2255654	3	123584488	A	G	MYLK
exm344954	3	124418879	C	T	KALRN
exm2269520	3	125182246	T	C	SNX4
exm346298	3	125872384	G	A	ALDH1L1
exm346807	3	126222856	T	C	UROC1
exm2269666_ver2	3	128127805	C	T	EEFSEC

exm348118	3	128181632	G	T	DNAJB8
exm348923	3	129130197	C	T	EFCAB12
exm348955_ver2	3	129140499	C	T	EFCAB12
exm349731	3	129305460	T	C	PLXND1
exm350082	3	130150310	C	A	COL6A5
exm-rs17404153_ver2	3	132163200	T	G	DNAJC13
exm351511	3	132337477	A	C	ACAD11
exm351823	3	133098806	T	C	TMEM108
exm351973	3	133191385	A	C	BFSP2
exm354186	3	138191448	T	A	ESYT3
exm354895	3	140178381	C	T	CLSTN2
exm2261397	3	142653680	A	G	RP11-372E1.4
rs7431684	3	143029090	C	A	SLC9A9
exm-rs7632299	3	143056467	A	G	SLC9A9
rs13099123	3	143100667	G	A	SLC9A9
rs6440168	3	143106228	G	A	SLC9A9
rs10513200	3	143128798	T	C	SLC9A9
rs11707045	3	143133000	A	T	SLC9A9
rs9873087	3	143394491	C	T	SLC9A9
rs935519	3	143404967	A	G	SLC9A9
exm-rs2800	3	143424822	C	T	SLC9A9
rs6787935	3	143446183	A	G	SLC9A9
rs9881418	3	143471832	A	G	SLC9A9
exm2255752	3	146186189	T	G	PLSCR2
exm357482	3	148757864	T	C	HLTF
exm358126	3	149498097	C	T	ANKUB1
exm358495	3	150391810	C	A	FAM194A
exm360527	3	155520356	T	C	C3orf33
exm361041_ver2	3	157098978	T	C	VEPH1
exm361308	3	158320597	T	C	MLF1
exm361315	3	158320703	A	C	MLF1
exm361349	3	158366900	G	A	GFM1
exm361431	3	158388780	T	C	LXN
exm2269557	3	158429009	T	C	RARRES1
exm361567_ver2	3	158537443	A	G	MFSD1
exm363791	3	169569432	G	C	LRRC31
exm2265526	3	169801658	A	G	GPR160
exm-rs2901705	3	171098888	A	G	TNIK
exm2255837	3	172046861	C	T	FNDC3B

exm364833	3	172046861	C	T	FNDC3B
exm-rs2861899	3	174659289	A	G	NAALADL2
exm2269691	3	174708659	C	T	NAALADL2
exm365405	3	174951756	T	C	NAALADL2
exm2269579	3	181020115	C	T	SOX2-OT
exm2269582	3	183074900	C	T	MCF2L2
exm367817	3	183777950	T	C	HTR3C
exm368455	3	183963381	C	T	ALG3
exm368853	3	184039304	G	A	EIF4G1
exm2255878	3	185229464	C	T	LIPH
exm2269586	3	185307363	G	T	SENP2
exm371125	3	186445052	G	T	KNG1
exm371724	3	187088923	A	G	RTP4
exm-rs4488809	3	189356261	C	T	TP63
exm372718	3	191179193	A	G	PYDC2
exm-rs2366659	3	192087751	C	T	FGF12
exm2265653	3	195305994	G	A	APOD
exm2255913	3	195306219	A	G	APOD
exm374438	3	195306219	A	G	APOD
exm374761	3	195497121	C	T	MUC4
exm374765	3	195497143	T	C	MUC4
exm375884	3	195594950	A	G	TNK2
exm-rs9859260	3	195800547	C	T	TFRC
exm-rs11924930	3	195928854	A	G	ZDHHC19
exm376611	3	196236401	A	G	C3orf43
exm376615	3	196236533	A	G	C3orf43
exm377081	3	196674749	C	T	PIGZ
exm377453	3	196921360	C	T	DLG1
exm377746	3	197495334	G	A	FYTTD1
exm378673	4	494194	A	C	PIGG
exm2062688	4	1019011	T	C	FGFRL1
exm381379	4	1729556	A	G	TACC3
exm-rs798766	4	1734239	T	C	TACC3
exm382777_ver4	4	2661342	G	A	FAM193A
exm384798	4	3526642	C	T	LRPAP1
exm-rs2008242	4	5221538	G	C	STK32B
exm385455	4	5448429	G	A	STK32B
exm386031	4	5830296	T	C	CRMP1
exm386791	4	6594947	C	A	MAN2B2

exm2265664	4	6598184	G	A	MAN2B2
exm386938	4	6616056	T	C	MAN2B2
exm2265818	4	6623332	G	A	MAN2B2
rs7654086	4	7465277	C	T	SORCS2
exm387603	4	7666180	A	C	SORCS2
exm389455	4	9922167	T	C	SLC2A9
exm390732	4	15688604	C	G	FAM200B
exm390801	4	15709192	A	G	BST1
exm2265821	4	17590671	A	G	LAP3
exm391493	4	17707449	T	C	FAM184B
exm-rs1379659	4	20620683	G	A	SLIT2
exm2269726	4	20807674	C	T	KCNIP4
exm2269907	4	21254331	C	T	KCNIP4
exm2265679	4	21627809	G	A	KCNIP4
exm2265680	4	22491705	A	G	GPR125
exm392810	4	25315754	C	G	ZCCHC4
rs4349588	4	26492362	G	A	CCKAR
exm394310	4	37851856	C	A	PGM2
exm394357	4	37962186	G	C	PTTG2
exm396361	4	40818179	C	T	APBB2
exm2269750	4	46039478	C	T	GABRG1
rs279871	4	46305733	G	A	GABRA2
rs279869	4	46307995	A	C	GABRA2
rs279861	4	46313325	C	G	GABRA2
rs279858	4	46314593	G	A	GABRA2
exm2269751	4	47052626	T	C	GABRB1
rs6824550	4	47073095	A	G	GABRB1
rs9999619	4	47282643	T	C	GABRB1
rs959160	4	47288955	G	C	GABRB1
rs6284	4	47322219	A	C	GABRB1
rs13107066	4	47378695	G	T	GABRB1
exm2256629	4	47504161	T	G	ATP10D
exm-rs10938494	4	47563448	A	G	ATP10D
exm397864	4	47667064	T	C	CORIN
exm398022	4	47901476	A	G	NFXL1
exm398163_ver3	4	48037926	G	A	NIPAL1
exm2265836	4	48088446	A	G	TXK
exm400150	4	55972974	A	T	KDR
rs7692461	4	56326166	C	T	CLOCK

exm400418	4	56475330	C	T	NMU
exm2256656	4	57953278	C	A	IGFBP7
rs17239094	4	62472569	G	A	LPHN3
rs13124636	4	62528171	G	A	LPHN3
rs13110933	4	62733437	C	T	LPHN3
exm401955	4	66509085	G	T	EPHA5
exm402538	4	68995529	T	C	TMPRSS11F
exm403666	4	70512773	G	A	UGT2A1
exm403668	4	70512801	G	A	UGT2A1
exm404372	4	71468348	C	G	AMBN
exm404395	4	71469604	T	C	AMBN
exm404534	4	71509314	T	C	ENAM
exm405308	4	73414286	C	T	ADAMTS3
exm406317	4	75695301	A	G	BTC
exm406427	4	76442052	A	G	THAP6
exm407311	4	77192868	A	G	FAM47E,FAM47E-STBD1
exm-rs6812193	4	77198986	T	C	FAM47E,FAM47E-STBD1
exm-rs9992101	4	77360431	A	G	SHROOM3
exm-rs17319721	4	77368847	A	G	SHROOM3
exm-rs13146355	4	77412140	A	G	SHROOM3
exm407614	4	77660731	C	G	SHROOM3
exm409505	4	82355804	T	C	RASGEF1B
exm409725	4	83582064	T	G	SCD5
exm411425	4	87769929	C	T	SLC10A6
exm411985	4	88533540	T	A	DSPP
exm412474	4	88732763	G	A	IBSP
exm412481	4	88732911	T	C	IBSP
exm412948	4	89306659	C	T	HERC6
exm413100	4	89397091	G	A	HERC5
exm-rs7671167	4	89883979	T	C	FAM13A
exm413767	4	90833184	T	C	MMRN1
exm2269797	4	91227681	T	C	CCSER1
exm2265854	4	93479275	A	G	GRID2
exm414017	4	93511396	T	C	GRID2
exm-rs1859156	4	95834034	T	G	BMPR1B
exm414511	4	96106322	A	G	UNC5C
exm2265736	4	98596032	A	G	STPG2
exm414738	4	99027159	C	T	STPG2

exm415447	4	100504664	C	T	MTTP
exm415878	4	102839287	A	G	BANK1
rs13117745	4	103478703	T	C	NFKB1
rs4648022	4	103496437	T	C	NFKB1
exm416893	4	106155199	T	C	TET2
exm417345	4	106861730	A	G	NPNT
exm417487	4	107157623	A	G	TBCK
exm418815	4	110901198	G	A	EGF
exm418845	4	110914427	A	T	EGF
exm2269833	4	121648617	T	C	PRDM5
exm-rs4505848	4	123132492	G	A	KIAA1109
rs2069762	4	123377980	G	T	IL2
exm422755	4	123664204	A	G	BBS12
exm424060	4	128723042	T	C	HSPA4L
exm424219	4	128814964	G	T	PLK4
exm424394	4	129043204	C	G	LARP1B
exm2269840	4	129052429	C	T	LARP1B
exm424430	4	129100643	G	A	LARP1B
exm424839	4	135121066	G	T	PABPC4L
exm424850	4	135121601	T	C	PABPC4L
exm-rs1492820	4	145650021	C	T	HHIP
exm2269861	4	147825919	C	T	TTC29
exm427437	4	147860980	A	G	TTC29
exm427742	4	148984321	G	A	ARHGAP10
rs13137823	4	149022906	G	A	NR3C2
rs17024387	4	149024260	A	G	NR3C2
rs1879828	4	149025117	A	T	NR3C2
rs13142954	4	149030027	A	G	NR3C2
rs6857487	4	149134380	A	T	NR3C2
rs10050229	4	149135547	G	A	NR3C2
rs2883930	4	149137625	C	G	NR3C2
rs5525	4	149356516	A	G	NR3C2
exm427849	4	149357475	C	T	NR3C2
rs5522	4	149357475	G	A	NR3C2
rs4696659	4	151191620	C	A	LRBA
exm427976	4	151199080	A	G	LRBA
exm427988	4	151207127	T	C	LRBA
rs4696084	4	151217933	C	A	LRBA
exm428315	4	151793903	C	T	LRBA

rs1875308	4	152201204	A	G	PRSS48
exm428580	4	152212603	T	G	PRSS48
exm428887	4	153690842	T	C	TIGD4
exm429400	4	154513627	G	A	KIAA0922
exm429406	4	154514965	C	T	KIAA0922
exm429663	4	154631563	C	T	RNF175
exm430223	4	155411883	A	G	DCHS2
exm-rs4691380	4	157720124	T	C	PDGFC
exm-rs6822892	4	157734675	G	A	PDGFC
rs11945868	4	158074816	C	T	GLRB
exm-rs8396	4	159630817	G	A	PPID
exm431666	4	159631991	T	G	PPID
exm432033	4	162307000	C	T	FSTL5
exm432042	4	162307312	A	C	FSTL5
rs4632602	4	164267129	C	T	NPY5R
exm2265787	4	164801854	G	A	MARCH1
exm2269985	4	165221971	T	G	MARCH1
exm2240639	4	165898682	T	C	TRIM61
exm433050	4	169083694	A	C	ANXA10
exm433190	4	169188780	C	T	DDX60
exm433265	4	169208334	T	C	DDX60
exm435039	4	177113779	C	T	SPATA4
exm435969	4	184367561	G	T	CDKN2AIP
exm2269890	4	185767941	C	T	RP11-701P16.5
exm436943	4	186111639	C	T	KIAA1430
exm2269891	4	186117162	T	C	KIAA1430
exm437309	4	186380243	A	C	CCDC110
exm437321_ver3	4	186380515	G	A	CCDC110
exm437335	4	186380846	A	T	CCDC110
exm437347	4	186381115	G	T	CCDC110
exm437581	4	186573817	T	C	SORBS2
exm437766	4	187077206	A	G	FAM149A
exm437786	4	187078785	A	G	FAM149A
exm439957_ver3	5	174106	A	G	PLEKHG4B
exm440434	5	434146	T	C	AHRR
exm440808	5	633915	A	G	CEP72
rs7732456	5	1392965	C	A	SLC6A3
rs2550936	5	1411256	C	A	SLC6A3
exm442885	5	5209210	A	G	ADAMTS16

exm2257310	5	6604787	T	C	NSUN2
exm-rs116364	5	6699224	C	T	LOC100505625
exm443751	5	7835555	G	C	C5orf49
exm2266056	5	11742668	A	G	CTNND2
exm445046	5	13788886	A	C	DNAH5
exm445106	5	13811775	C	T	DNAH5
exm445519	5	13931340	T	C	DNAH5
exm2270014	5	14239606	T	G	TRIO
exm2270019	5	19513668	G	T	CDH18
exm2270191	5	21879902	C	T	CDH12
exm449239	5	34998877	A	C	AGXT2
exm450113	5	36219710	T	C	NADK2
exm450340	5	36985303	G	A	NIPBL
exm451222	5	38412674	T	C	EGFLAM
exm2270038	5	38842959	T	C	CTD-2127H9.1
exm451483_ver3	5	38884071	G	T	OSMR
exm2265935	5	40998851	A	G	MROH2B
exm452586	5	41008780	A	G	MROH2B
exm452617	5	41018798	G	A	MROH2B
exm452681	5	41049397	C	T	MROH2B
exm452718	5	41061716	A	G	MROH2B
exm-rs13188386	5	42473555	A	G	GHR
exm2266081	5	50057729	G	A	PARP8
exm454288	5	52229745	G	T	ITGA1
exm2270052	5	53256679	G	T	ARL15
exm2257266	5	53815240	C	G	SNX18
exm-rs6859219	5	55438580	A	C	ANKRD55
exm2266083	5	58032485	G	A	RAB3C
exm2270058	5	58095222	C	T	RAB3C
exm458001	5	65350481	T	C	ERBB2IP
exm458308	5	66460390	A	G	MAST4
exm2266087	5	67596088	G	A	PIK3R1
exm458822	5	68568858	T	C	CDK7
exm2265959	5	73986648	A	G	HEXB
exm-rs457717	5	75920972	A	G	IQGAP2
exm462061	5	75932965	G	C	IQGAP2
exm462082	5	75948650	A	G	IQGAP2
rs7728378	5	76259350	C	T	CRHBP
rs10055255	5	76263993	T	A	CRHBP

exm2261974	5	78190471	A	G	ARSB
exm463278	5	78573778	A	G	JMY
exm463611	5	79028513	G	A	CMYA5
exm463612	5	79028586	T	C	CMYA5
exm463651	5	79029726	A	T	CMYA5
exm463675	5	79030212	T	C	CMYA5
exm463679	5	79030338	G	A	CMYA5
exm463771	5	79032666	T	C	CMYA5
exm463774	5	79032711	A	G	CMYA5
exm463840	5	79034662	G	C	CMYA5
exm464423	5	79815658	T	C	FAM151B
exm464480	5	79855372	A	G	ANKRD34B
exm2257363	5	80376142	T	C	RASGRF2
exm465244	5	82491674	C	T	XRCC4
exm465267	5	82648943	A	G	XRCC4
exm465372	5	82816190	G	A	VCAN
exm466650	5	90016871	A	G	GPR98
exm466893	5	90107108	A	G	GPR98
exm466906	5	90119324	G	A	GPR98
exm467278	5	93987532	A	C	ANKRD32
exm467536	5	94784082	C	T	FAM81B
exm467908	5	95011189	G	A	SPATA9
exm468058	5	95236459	T	C	ELL2
exm468164	5	95751785	C	T	PCSK1
exm468233	5	96076487	A	G	CAST
exm468404	5	96118866	T	C	ERAP1
exm468439	5	96122210	T	C	ERAP1
exm468484	5	96129535	C	T	ERAP1
exm468496	5	96130836	C	T	ERAP1
exm468540	5	96139464	T	C	ERAP1
exm469544	5	102338811	G	A	PAM
exm469766	5	102537285	G	A	PPIP5K2
exm469767	5	102537298	T	C	PPIP5K2
exm470507	5	110439509	G	A	WDR36
exm2270108	5	111103258	C	T	NREP
exm2256863	5	113801423	A	G	KCNN2
exm473088	5	118513876	A	G	DMXL1
rs2036565	5	118532871	C	T	DMXL1
exm473387	5	120021817	A	C	PRR16

exm2270122	5	122471989	C	T	PRDM6
exm2256893	5	123984763	C	T	ZNF608
exm-rs13182402	5	125918148	G	A	ALDH7A1
exm475532	5	128301885	G	T	SLC27A6
exm478123	5	133481467	A	G	TCF7
exm478196	5	133642326	C	T	CDKL3
exm479813	5	137244517	G	A	PKD2L2
exm481278	5	138707916	T	G	SLC23A1
exm481804	5	139231255	T	C	NRG2
exm487907	5	140724591	T	G	PCDHGA3
exm488185	5	140739737	C	G	PCDHGB2
exm488340	5	140745224	A	G	PCDHGA5
exm488398	5	140750020	A	T	PCDHGB3
exm488747	5	140768709	A	G	PCDHGB4
exm489181	5	140789933	A	G	PCDHGB6
exm489400	5	140799812	A	G	PCDHGB7
exm490184_ver2	5	141021294	G	T	FCHSD1
rs6188	5	142680344	T	G	NR3C1
rs33388	5	142697295	A	T	NR3C1
rs852980	5	142700856	G	C	NR3C1
rs852982	5	142705421	A	G	NR3C1
rs2918417	5	142726170	A	G	NR3C1
rs6877893	5	142727193	G	A	NR3C1
rs10482633	5	142750533	C	A	NR3C1
rs1866388	5	142759785	G	A	NR3C1
rs10041520	5	142776684	T	C	NR3C1
rs4634384	5	142780697	C	T	NR3C1
rs10052957	5	142786701	A	G	NR3C1
rs7701443	5	142792650	G	A	NR3C1
rs4607376	5	142796532	G	A	NR3C1
exm2270143	5	143570841	T	C	KCTD16
exm2266127	5	145215872	G	A	PRELID2
exm491638	5	145393364	C	T	SH3RF2
exm491747	5	145477855	C	T	PLAC8L1
exm491774	5	145499996	T	C	LARS
exm491808	5	145508636	C	T	LARS
exm2257013	5	145519821	A	G	LARS
exm491847	5	145519821	A	G	LARS
exm492252	5	145894896	C	G	GPR151

exm493168	5	148206440	A	G	ADRB2
rs1042713	5	148206440	A	G	ADRB2
rs1042717	5	148206646	A	G	ADRB2
rs1042719	5	148207447	C	G	ADRB2
exm-rs32579	5	149210848	A	G	PPARGC1B
exm494090	5	149212243	C	G	PPARGC1B
rs7732671	5	149212243	C	G	PPARGC1B
exm494954	5	149509508	A	G	PDGFRB
rs1549914	5	149608664	G	T	CAMK2A
exm495379	5	149681846	A	C	ARSI
exm495687	5	149826526	T	C	RPS14
exm496604	5	150632832	A	G	GM2A
exm496698	5	150667016	C	T	SLC36A3
exm496984	5	150891772	G	C	FAT2
exm497101	5	150908813	A	G	FAT2
rs17519383	5	152958550	A	G	GRIA1
rs6555849	5	156529647	A	G	HAVCR2
exm2266032	5	159369429	A	G	ADRA1B
rs7737796	5	159369429	A	G	ADRA1B
rs11959228	5	161127111	A	G	GABRA6
exm501055	5	161128627	T	C	GABRA6
rs34907804	5	161128627	A	G	GABRA6
exm501827	5	167858371	T	G	WWC1
exm501828	5	167858372	G	T	WWC1
exm502158_ver2	5	168112927	C	G	SLIT3
exm-rs11743823	5	168164744	T	C	SLIT3
exm-rs169082	5	169074056	A	G	DOCK2
exm2266042	5	169078934	G	A	DOCK2
exm2270266	5	169178278	C	T	DOCK2
exm502669	5	169310213	C	A	FAM196B
exm-rs315717	5	169685163	C	T	LCP2
exm2266044	5	169865015	G	A	KCNIP1
exm504154	5	172550204	T	C	CREBRF
rs5326	5	174870196	A	G	DRD1
exm504946	5	175811233	T	C	NOP16
exm507779	5	176951914	A	G	FAM193B
exm-rs6879260	5	179731014	T	C	GFPT2
exm511291	5	180218668	A	G	MGAT1
exm511440	5	180338368	G	A	BTNL8

exm511459	5	180374523	A	G	BTNL8
exm2270175	5	180469713	C	T	BTNL9
exm511728	5	180582604	A	G	OR2V2
exm-rs9378305	6	1703290	T	C	GMDS
exm2270278	6	2355675	T	C	GMDS-AS1
exm512621	6	2623930	A	G	C6orf195
exm512629	6	2624053	A	T	C6orf195
exm512688	6	2685775	A	G	MYLK4
exm514015	6	5260936	C	A	LYRM4
rs2326766	6	6393064	A	G	LY86-AS1
exm-rs2326810	6	6612467	C	G	LY86-AS1
rs1675414	6	10412188	G	A	TFAP2A
exm515961	6	10702799	A	G	PAK1IP1
exm516076	6	10775600	A	G	MAK
exm516257	6	10898308	T	C	SYCP2L
exm516956	6	12125772	G	A	HIVEP1
exm-rs219950	6	12165363	T	C	HIVEP1
rs3213207	6	15628102	G	A	DTNBP1
exm518640	6	17665479	C	G	NUP153
exm518663	6	17675246	C	T	NUP153
exm-rs12199222	6	17699322	T	G	NUP153
exm-rs6908425	6	20728731	T	C	CDKAL1
exm519715	6	24403455	T	C	MRS2
exm519915	6	24463020	C	G	GPLD1
exm2266297	6	25127176	A	G	CMAHP
exm-rs1183201	6	25823444	T	A	SLC17A1
exm521676	6	26056604	A	G	HIST1H1C
exm522492	6	26247198	G	A	HIST1H4G
exm522755	6	26392629	A	G	BTN2A2
exm522794	6	26405816	C	T	BTN3A1
exm523249	6	26505362	G	A	BTN1A1
exm523285_ver4	6	26509330	C	G	BTN1A1
exm523720	6	27293107	A	G	VN1R10P
exm523721	6	27293114	A	T	VN1R10P
exm523723	6	27293147	A	T	VN1R10P
exm-rs7770592	6	29395747	A	C	OR11A1
exm-rs29234	6	29624112	G	T	MOG
exm-rs3117286	6	29629774	C	T	MOG
exm-rs926828	6	29638425	A	G	MOG

exm526368	6	29640785	A	T	ZFP57
exm-rs2747419	6	29642194	T	G	ZFP57
exm526425	6	29691303	A	G	HLA-F
exm-rs1736926	6	29692562	T	C	HLA-F
exm526477	6	29694680	C	A	HLA-F
exm-rs2272874	6	29696245	G	A	HLA-F-AS1
exm-rs1610613	6	29710963	A	G	HLA-F-AS1
exm-rs2072894	6	29713036	A	G	HLA-F-AS1
exm-rs6901686	6	29715721	A	G	HLA-F-AS1
exm-rs733841	6	29717980	T	C	IFITM4P
exm-rs909728	6	29719561	A	G	IFITM4P
exm2274136_ver4	6	29760029	C	G	LOC554223
exm527045	6	30071330	T	C	TRIM31
exm-rs2074483	6	30076755	T	C	TRIM31
exm-rs3815081	6	30114074	C	T	TRIM40
exm527306	6	30131546	G	A	TRIM15
exm527308	6	30131585	A	G	TRIM15
exm-rs2074477	6	30132035	T	C	TRIM15
exm-rs9368624	6	30135977	A	G	TRIM15
exm527331	6	30136302	G	C	TRIM15
exm-rs1029239	6	30138162	C	G	TRIM15
exm-rs9380156	6	30141042	G	A	TRIM15
exm-rs2021107_ver4	6	30228138	T	C	HCG17,HLA-L
exm-rs6931763	6	30311932	C	A	TRIM39-RPP21
exm527557	6	30313340	C	G	RPP21,TRIM39-RPP21
exm-rs2524222	6	30511170	A	G	GNL1
exm-rs1058318	6	30512163	A	G	GNL1
exm-rs2844715	6	30516353	G	C	GNL1
exm-rs2516647	6	30526845	A	C	PRR3
exm-rs2074503	6	30530496	A	G	PRR3
exm-rs2269710	6	30539952	G	A	ABCF1
exm-rs2269709	6	30540890	G	A	ABCF1
exm527762	6	30548210	G	A	ABCF1
exm-rs1264440	6	30551286	T	C	ABCF1
exm-rs3130244	6	30552937	A	C	ABCF1
exm-rs1264437	6	30553871	G	A	ABCF1
exm-rs6918685	6	30556016	T	C	ABCF1
exm-rs2252745	6	30579315	C	T	PPP1R10
exm-rs1076829	6	30627216	C	T	DHX16

exm-rs11965214	6	30647005	G	C	PPP1R18
exm-rs1075496	6	30658239	T	G	NRM
exm-rs8512	6	30711357	T	C	IER3
exm-rs1264323	6	30855907	T	C	DDR1
exm-rs2239518	6	30865725	T	C	DDR1
exm529001	6	30882689	T	C	VARS2
exm529006	6	30882803	A	G	VARS2
exm529161	6	30899571	T	C	SFTA2
exm-rs2286655_ver4	6	30899746	T	C	SFTA2
exm-rs2253705	6	30900094	A	G	SFTA2
exm-rs2844700	6	30912900	T	C	DPCR1
exm-rs3130782	6	30914843	T	C	DPCR1
exm529258	6	30919878	C	G	DPCR1
exm529266	6	30920086	T	C	DPCR1
exm-rs2844677	6	30955359	T	C	MUC21
exm-rs2523915	6	30973358	A	T	MUC22
exm-rs2523921	6	30975090	C	T	MUC22
exm-rs13191258	6	30978717	T	C	MUC22
exm-rs7755364	6	30981715	T	G	MUC22
exm-rs2523897	6	30993958	T	C	MUC22
exm-rs3130991	6	31087354	T	C	CDSN,PSORS1C1
exm-rs3094208	6	31090672	C	T	PSORS1C1
exm-rs3095312	6	31090837	G	C	PSORS1C1
exm-rs3095302	6	31093066	T	C	PSORS1C1
exm-rs3131003	6	31093482	A	G	PSORS1C1
exm-rs3815087	6	31093587	T	C	PSORS1C1
exm-rs3823418	6	31100942	A	G	PSORS1C1
exm-rs4084091	6	31101401	A	G	PSORS1C1
exm-rs3130573	6	31106268	G	A	PSORS1C1,PSORS1C2
exm-rs1265078	6	31112602	G	C	CCHCR1
exm529934	6	31112737	A	C	CCHCR1
exm-rs746647	6	31114182	C	T	CCHCR1
exm529970	6	31116246	A	G	CCHCR1
exm-rs1265112	6	31118019	G	A	CCHCR1
exm530016	6	31122482	A	G	CCHCR1
exm530018	6	31122500	A	G	CCHCR1
exm-rs720465	6	31125777	T	G	CCHCR1
exm-rs3130503_ver2	6	31137165	A	G	POU5F1
exm-rs879882	6	31139452	T	C	POU5F1

exm-rs887468	6	31141523	A	G	PSORS1C3
exm-rs3131018	6	31143582	T	G	PSORS1C3
exm-rs3094609	6	31165566	A	G	HCG27
exm530363	6	31239417	T	C	HLA-C
exm-rs2596503	6	31320810	T	C	HLA-B
exm530461	6	31322911	T	C	HLA-B
exm-rs1063632	6	31378510	A	G	MICA
exm530708	6	31379109	A	G	MICA
exm530732	6	31380198	T	C	MICA
exm530736	6	31382882	A	C	MICA
exm530981	6	31525912	C	T	NFKBIL1
exm530990	6	31526080	A	G	NFKBIL1
exm531009	6	31540556	C	T	LTA
exm-rs3093662	6	31544189	G	A	TNF
exm-rs4947328	6	31561747	G	A	NCR3
exm2270462	6	31583827	G	T	AIF1
exm-rs2857597	6	31585000	A	T	AIF1
exm-rs2857697	6	31585219	A	G	AIF1
exm-rs2736176	6	31587561	G	C	PRRC2A
exm-rs2857694	6	31587870	T	A	PRRC2A
exm-rs2844472	6	31589676	C	T	PRRC2A
exm-rs2736171	6	31595487	C	T	PRRC2A
exm531302	6	31595882	C	A	PRRC2A
exm531420	6	31601344	T	C	PRRC2A
exm-rs2261033_ver4	6	31603591	C	T	PRRC2A
exm531492	6	31604044	T	G	PRRC2A
exm-rs1077393_ver2	6	31610529	C	T	BAG6
exm531593	6	31610686	G	A	BAG6
exm-rs3130050	6	31618761	G	A	BAG6
exm531720	6	31627523	C	T	C6orf47
exm-rs805262	6	31628733	A	G	C6orf47;GPANK1
exm-rs707919_ver3	6	31641139	C	T	LY6G5B
exm-rs3131382	6	31707730	A	G	MSH5-SAPCD1
exm-rs707939	6	31726688	T	G	MSH5-SAPCD1
exm532575	6	31777946	T	C	HSPA1L
exm-rs660594	6	31837250	G	A	SLC44A4
exm532838	6	31838441	T	C	SLC44A4
exm532862	6	31839309	C	T	SLC44A4
exm-rs614549	6	31840625	C	T	SLC44A4

exm532878	6	31842598	A	T	SLC44A4
exm-rs2763982	6	31872551	C	G	C2
exm-rs644045	6	31883957	T	C	C2
exm-rs3130683	6	31888367	C	T	C2
exm-rs3020644	6	31894626	G	A	C2
exm-rs537160	6	31916400	T	C	CFB
exm-rs4151657	6	31917540	C	T	CFB
exm-rs2072633	6	31919578	T	C	CFB
exm-rs630379	6	31922254	T	G	NELFE
exm-rs440454_ver4	6	31927342	T	C	SKIV2L
exm-rs419788	6	31928799	A	G	SKIV2L
exm533406	6	31929014	A	C	SKIV2L
exm-rs592229	6	31930441	G	T	SKIV2L
exm-rs6941112	6	31946614	A	G	STK19
exm-rs389883	6	31947460	C	A	STK19
exm-rs7774197	6	32046275	C	A	TNXB
exm-rs3134954	6	32071893	G	A	TNXB
exm-rs411337	6	32077380	A	G	TNXB
exm-rs3131283	6	32119898	A	G	PRRT1
exm534604	6	32122386	C	G	PPT2
exm-rs3134943	6	32147761	A	G	RNF5P1
exm-rs1800684	6	32151994	T	A	AGER
exm-rs3132946	6	32190028	A	G	NOTCH4
exm-rs9268148	6	32259527	G	A	C6orf10
exm-rs6935269	6	32260350	C	T	C6orf10
exm535239	6	32261507	C	T	C6orf10
exm535243	6	32261630	A	G	C6orf10
exm-rs6909427	6	32268701	G	T	C6orf10
exm-rs9268168	6	32272510	T	C	C6orf10
exm-rs6457536	6	32273765	G	A	C6orf10
exm-rs7341328	6	32275194	A	G	C6orf10
exm-rs3132958	6	32297901	A	G	C6orf10
exm-rs3132959	6	32298942	G	A	C6orf10
exm-rs3129900	6	32305979	G	T	C6orf10
exm-rs3129904	6	32310396	A	G	C6orf10
exm-rs6904320	6	32312082	A	G	C6orf10
exm-rs3132963	6	32320153	C	T	C6orf10
exm-rs1265759	6	32322393	G	A	C6orf10
exm-rs3129907	6	32323745	G	A	C6orf10

exm-rs2395150	6	32326045	G	A	C6orf10
exm-rs3129923	6	32333195	A	G	C6orf10
exm-rs2143462	6	32335204	T	C	C6orf10
exm-rs3129931	6	32335516	C	T	C6orf10
exm-rs3129934	6	32336187	T	C	C6orf10
exm-rs3129938	6	32336495	T	A	C6orf10
exm-rs2273017	6	32337630	C	T	C6orf10
exm535341	6	32337686	A	G	C6orf10
exm-rs2073044	6	32338986	A	G	C6orf10
exm-rs2239802	6	32411846	G	C	HLA-DRA
exm535799	6	32497962	T	C	HLA-DRB5
exm536297	6	32629755	A	G	HLA-DQB1
exm-rs2213568	6	32711576	T	G	HLA-DQA2
exm-rs9276431	6	32712247	T	C	HLA-DQA2
exm536573	6	32714143	A	G	HLA-DQA2
exm-rs3213485	6	32724719	A	G	HLA-DQB2
exm-rs2301271	6	32725193	T	C	HLA-DQB2
exm-rs7453920	6	32730012	A	G	HLA-DQB2
exm-rs2051549	6	32730086	C	T	HLA-DQB2
exm-rs1573649	6	32731258	C	T	HLA-DQB2
exm-rs6902723	6	32731960	G	A	HLA-DQB2
exm-rs2857114	6	32779996	C	T	HLA-DOB
exm536749	6	32800412	T	C	TAP2
exm-rs241424	6	32804934	T	C	TAP2
exm-rs2239701	6	32805049	G	A	TAP2
exm-rs2071465	6	32805470	C	G	TAP2
exm-rs2071544_ver3	6	32806121	G	A	TAP2
exm-rs2071552	6	32806461	C	T	TAP2
exm-rs4713598	6	32806786	G	T	TAP2
exm-rs3763349	6	32808232	T	C	PSMB8
exm-rs9357155	6	32809848	A	G	PSMB8
exm-rs9276810	6	32810443	A	G	PSMB8
exm536844_ver3	6	32811629	T	G	PSMB8
exm-rs3198005	6	32813214	T	C	TAPSAR1
exm-rs2071538	6	32818678	T	C	TAP1
exm-rs2284190	6	32819517	C	T	TAP1
exm-rs4713600	6	32822858	T	G	PSMB9
exm537013	6	32825090	A	G	PSMB9
exm-rs241410	6	32861651	G	C	LOC100294145

exm-rs10751	6	32902583	T	C	HLA-DMB
exm-rs151719	6	32903900	G	A	HLA-DMB
exm-rs2071556	6	32904601	C	A	HLA-DMB
exm537079	6	32906586	T	C	HLA-DMB
exm537081	6	32906652	A	T	HLA-DMB
exm537082	6	32906716	C	T	HLA-DMB
exm537102	6	32917411	T	C	HLA-DMA
exm537105	6	32917498	G	C	HLA-DMA
exm-rs10484568	6	32935751	G	A	BRD2
exm537234	6	32945625	T	C	BRD2
exm-rs86567	6	32976759	C	A	HLA-DOA
exm-rs3117035	6	33086249	A	G	HLA-DPB2
exm538307	6	33256471	T	C	WDR46
exm-rs465223	6	33359716	C	G	KIFC1
exm538829	6	33382288	G	A	PHF1
exm538877	6	33384473	T	C	CUTA
exm-rs210138	6	33542538	G	A	BAK1
exm-rs375555_ver3	6	33557741	C	T	LINC00336
exm-rs471942_ver2	6	33696785	T	C	IP6K3
exm-rs9469578	6	33706479	T	C	IP6K3
exm540116	6	34498328	T	C	PACSIN1
exm-rs2814993	6	34618893	A	G	C6orf106
exm540479	6	34850826	C	G	TAF11
rs3800373	6	35542476	G	T	FKBP5
rs7757037	6	35548236	A	G	FKBP5
rs992105	6	35555183	C	A	FKBP5
rs9296158	6	35567082	A	G	FKBP5
rs2395634	6	35567760	A	G	FKBP5
rs3777747	6	35579002	G	A	FKBP5
rs6926133	6	35579375	A	C	FKBP5
rs9368878	6	35585614	C	T	FKBP5
rs3798347	6	35601776	A	T	FKBP5
rs1591365	6	35604107	G	A	FKBP5
rs1360780	6	35607571	T	C	FKBP5
rs6912833	6	35617585	A	T	FKBP5
rs9394309	6	35621781	G	A	FKBP5
rs17614642	6	35621921	C	T	FKBP5
rs4713904	6	35625147	C	T	FKBP5
rs9380525	6	35633038	G	C	FKBP5

rs9470080	6	35646435	T	C	FKBP5
rs6457839	6	35648830	C	T	FKBP5
rs10947563	6	35653437	G	A	FKBP5
rs3800372	6	35655245	C	T	FKBP5
rs9380526	6	35658327	C	T	FKBP5
rs7763535	6	35662106	C	A	FKBP5
rs943297	6	35667860	A	G	FKBP5
rs4713916	6	35669983	A	G	FKBP5
rs9462104	6	35674845	C	T	FKBP5
rs12200498	6	35685955	A	G	FKBP5
rs7751693	6	35695274	A	G	RP3-510O8.4
rs2817040	6	35705606	A	G	ARMC12
rs2766543	6	35708634	G	T	ARMC12
rs2766545	6	35713031	A	G	ARMC12
exm541783	6	35923246	C	T	SLC26A8
exm541824	6	35960390	T	C	SLC26A8
rs851006	6	36065185	A	G	MAPK14
exm2266303	6	38250780	G	A	BTBD9
exm543785	6	38747770	A	G	DNAH8
exm543843	6	38773293	A	G	DNAH8
exm2257793	6	39020542	C	T	GLP1R
exm544393	6	39041502	A	C	GLP1R
exm-rs10947789	6	39174922	C	T	KCNK5
exm544720	6	39398931	T	C	KIF6
exm2266183	6	40546850	G	A	LRFN2
exm545598	6	41166155	C	T	TREML2
exm545628	6	41196605	T	C	TREML4
exm545654	6	41197857	C	T	TREML4
exm546833	6	42224546	T	A	TRERF1
exm2270317	6	42658752	C	T	UBR2
exm547484	6	42891022	A	G	PTCRA
exm547804	6	42980218	T	G	MEA1
exm548266	6	43029288	A	G	KLC4
exm548312	6	43034855	T	C	KLC4
rs2010963	6	43738350	C	G	VEGFA
rs833068	6	43742527	A	G	VEGFA
rs833069	6	43742579	G	A	VEGFA
rs3025039	6	43752536	T	C	VEGFA
rs693955	6	44191920	T	G	SLC29A1

exm551164	6	44198362	C	T	SLC29A1
exm551752	6	44310854	A	G	SPATS1
exm2270469	6	46232325	T	C	RCAN2
exm553199	6	46977783	T	C	GPR110
exm553335	6	47251758	T	C	TNFRSF21
exm-rs2206277_ver2	6	50798526	A	G	TFAP2B
exm555045	6	51910905	C	T	PKHD1
exm2270324	6	52319251	T	C	EFHC1
exm555647	6	52617731	C	G	GSTA2
exm556371	6	53989526	G	A	MLIP
exm556701	6	55142337	A	G	HCRTR2
exm556928	6	55924962	G	A	COL21A1
exm556966	6	55988870	C	G	COL21A1
exm2257876	6	56274973	C	T	RNU6-71P
exm2262157	6	65455894	G	A	EYS
exm559126	6	69666684	A	G	BAI3
exm560013_ver2	6	71546702	T	C	SMAP1
exm2273422	6	76425594	A	C	SENP6
exm563170	6	83838973	G	A	DOPEY1
exm564903	6	88240570	T	C	RARS2
exm565486	6	89967498	A	G	GABRR2
exm566408	6	90482397	C	T	MDN1
exm566440	6	90497589	C	A	MDN1
exm567347	6	97414949	T	C	KLHL32
exm568283	6	100964147	G	C	ASCC3
exm568499	6	101296389	A	G	ASCC3
exm570673	6	109764535	T	G	SMPD2
exm-rs1046943	6	109783941	C	T	ZBTB24
exm571592	6	110714439	C	G	DDO
exm572270	6	111696091	C	T	REV3L
exm572464	6	111913070	A	G	TRAF3IP2
exm572471	6	111913262	T	C	TRAF3IP2
exm574096	6	117114290	G	A	GPRC6A
exm574145	6	117130544	C	A	GPRC6A
exm575021_ver2	6	119137423	A	G	MCM9
exm2270517	6	124841336	T	C	NKAIN2
exm577826	6	130031215	T	C	ARHGAP18
exm579636	6	133119564	A	C	SLC18B1
exm2266257	6	133655858	G	A	EYA4

exm-rs9402515	6	133860345	G	A	RP3-323P13.2
exm2273428	6	135286230	C	T	HBS1L
exm2270409	6	135525396	T	G	MYB
exm580391	6	135751024	A	G	AHI1
exm580413	6	135768282	T	C	AHI1
exm-rs610604	6	138199417	C	A	TNFAIP3
exm2270414	6	139134754	C	T	ECT2L
exm582578_ver3	6	139610006	T	C	TXLNB
exm582685	6	142400020	T	G	NMBR
exm582791	6	142691549	C	A	GPR126
exm-rs3817928	6	142750516	G	A	GPR126
exm583060	6	143092754	A	G	HIVEP2
exm583174	6	143095633	G	C	HIVEP2
exm-rs198683_ver2	6	143200547	C	T	HIVEP2
exm584288	6	146126580	A	G	FBXO30
exm584901	6	147680359	A	G	STXBP5
exm585390	6	149783095	C	T	ZC3H12D
exm585825	6	150209717	G	C	RAET1E
exm585840	6	150210681	G	A	RAET1E
exm585841	6	150210685	T	C	RAET1E
exm585843	6	150210723	T	C	RAET1E
exm2257591	6	150211100	C	T	RAET1E
exm2270429	6	150525260	C	T	PPP1R14C
exm586862	6	151673070	A	G	AKAP12
exm-rs1999805	6	152068364	C	T	ESR1
rs9478329	6	152707063	T	C	SYNE1
exm588475	6	152771967	G	A	SYNE1
rs9397510	6	152797065	A	G	SYNE1
rs7751588	6	152797353	T	C	SYNE1
exm-rs9478751	6	157441050	G	A	ARID1B
exm2270537	6	159096121	C	T	SYTL3
exm591242	6	159407447	A	G	RSPH3
exm591368	6	159462424	T	C	TAGAP
exm591411	6	159621087	T	C	FNDC1
exm591460	6	159650978	G	A	FNDC1
exm591464	6	159652931	G	C	FNDC1
exm591565	6	159655084	C	G	FNDC1
exm591583	6	159655383	A	G	FNDC1
exm591620	6	159660879	C	A	FNDC1

exm2266281	6	163697439	G	A	PACRG
exm595364	6	168463624	A	G	FRMD1
exm595921	6	170033086	A	G	WDR27
exm595950	6	170038696	A	G	WDR27
exm596063	6	170070723	G	A	WDR27
exm596680	6	170862300	C	G	PSMB1
exm596875	7	299863	T	C	FAM20C
exm-rs10256972	7	1039003	A	C	C7orf50
exm601847	7	4830463	A	G	AP5Z1
exm605073	7	6731672	C	G	ZNF12
exm-rs10259085	7	7268431	C	T	C1GALT1
exm605455	7	7561580	C	T	COL28A1
exm606184_ver3	7	12269417	G	C	TMEM106B
exm606380	7	12610594	G	A	SCIN
exm2266378	7	18504411	G	A	HDAC9
exm2270730	7	19980396	C	T	AC005062.2
exm607622	7	20193979	G	T	MACC1
exm607734	7	20201458	A	G	MACC1
exm607833	7	20666235	C	G	ABCB5
exm607874	7	20685484	A	C	ABCB5
exm607906	7	20691047	C	T	ABCB5
exm608825	7	21901540	C	T	DNAH11
exm608902	7	21939032	A	C	DNAH11
rs2069849	7	22771156	T	C	IL6
exm609728	7	23728916	A	G	FAM221A
exm609730	7	23728931	C	T	FAM221A
exm609764	7	23737891	G	A	FAM221A
exm609801	7	23757162	C	G	STK31
exm610374	7	25207974	C	T	C7orf31
exm610416	7	25267934	C	T	NPVF
exm610667	7	26678881	A	G	C7orf71
exm-rs11765845	7	28391142	A	G	CREB5
exm-rs2252521	7	29041190	T	C	LOC100506497
exm611916	7	29980471	T	C	SCRN1
rs975537	7	30697357	T	A	CRHR2
rs1076292	7	30712701	C	G	CRHR2
rs2014663	7	30715573	G	A	CRHR2
rs1003929	7	30719049	T	C	CRHR2
exm612529	7	30795331	G	A	INMT

exm612598	7	30831082	C	T	FAM188B
exm613315	7	32598630	A	T	AVL9
exm613894	7	34818113	T	A	NPSR1
rs324981	7	34818113	T	A	NPSR1
exm2270600	7	34821428	C	T	NPSR1-AS1
exm613954	7	34889182	G	A	NPSR1
exm614365	7	36445856	G	A	ANLN
exm614829	7	37923923	C	T	NME8
exm2273432	7	40134678	G	A	CDK13
exm2273433	7	42003792	T	A	GLI3
exm616052	7	42004062	A	G	GLI3
exm616071	7	42004664	T	C	GLI3
exm616266	7	42088222	T	C	GLI3
exm618737	7	45016621	T	C	MYO1G
exm2266521	7	45703971	G	A	ADCY1
exm619369	7	47333332	A	G	TNS3
exm619703	7	47851623	T	C	PKD1L1
exm619709	7	47852837	T	C	PKD1L1
exm619818	7	47879049	A	G	PKD1L1
exm620572	7	48313155	G	A	ABCA13
exm620784	7	48349647	T	C	ABCA13
exm621095	7	49842377	C	A	VWC2
exm2258377	7	50097697	G	A	ZPBP
rs11575543	7	50530869	T	C	DDC
exm621342	7	50530987	T	C	DDC
rs11575522	7	50535395	A	G	DDC
rs732215	7	50544063	G	T	DDC
rs11575343	7	50596347	A	G	DDC
rs3779074	7	50612202	T	C	DDC
exm-rs10248619	7	50751090	T	C	GRB10
exm-rs2108349	7	50786663	C	T	GRB10
exm-rs6943153	7	50791579	T	C	GRB10
exm621677	7	51097063	T	G	COBL
rs759167	7	55127784	T	G	EGFR
rs1024750	7	55128731	A	G	EGFR
exm621975	7	55229255	A	G	EGFR
exm623389	7	64389089	C	A	ZNF273
exm623536	7	64452738	A	G	ERV3-1
exm626111	7	73279482	T	C	WBSCR28

exm628168	7	75687396	A	G	MDH2
exm628398	7	76028057	A	G	SRCRB4D
exm2154878	7	76069926	G	A	ZP3
exm629157	7	77247821	G	A	PTPN12
exm629355	7	77552127	G	A	PHTF2
rs802441	7	86287103	T	C	GRM3
exm2264265	7	86372884	C	A	GRM3
rs7806785	7	86446316	G	C	GRM3
exm632310	7	88423881	C	T	C7orf62
exm632473	7	88965141	G	A	ZNF804B
exm632825	7	89938588	C	G	C7orf63
exm632829	7	89938680	T	C	C7orf63
exm634869	7	93090133	T	C	CALCR
exm635552	7	94903477	A	G	PPP1R9A
rs2049649	7	94949329	C	T	PON1
exm638128	7	99081730	G	A	ZNF789
exm2266552	7	99266318	A	G	CYP3A5
exm639333	7	99669802	G	A	ZNF3
exm640367	7	99817859	A	G	PVRIG
exm640640	7	100014711	C	T	ZCWPW1
exm640997	7	100151719	A	G	AGFG2
exm642168_ver4	7	100361675	A	G	ZAN
exm642213	7	100365613	T	G	ZAN
exm642222	7	100367573	C	T	ZAN
exm643928	7	100675128	A	G	MUC17
exm646305	7	101928495	G	C	SH2B2
exm647101	7	102760511	C	A	NAPEPLD
exm-rs4298437	7	103625877	T	C	RELN
exm2270777	7	107815158	T	C	NRCAM
exm2258065	7	111732556	G	A	DOCK4
exm-rs17159640_ver2	7	112086333	T	A	IFRD1
exm653414	7	121653250	A	G	PTPRZ1
exm654578	7	124386655	A	G	GPR37
exm-rs17864092	7	126639604	C	T	GRM8
exm2273439	7	128371298	T	C	FAM71F1
exm657611	7	129915016	G	A	CPA2
exm2244997	7	129984630	A	G	CPA5
exm-rs10488172	7	133335176	G	T	EXOC4
exm659790	7	135048804	T	C	CNOT4

exm-rs2551762	7	135162927	A	G	CNOT4
exm2266481	7	135354582	A	G	C7orf73
rs322239	7	136956607	C	A	PTN
exm660443	7	137374693	A	G	DGKI
exm660528	7	137600690	C	T	CREB3L2
exm660736	7	138312122	A	C	SVOPL
exm662588	7	140227293	G	A	DENND2A
exm-rs10237118	7	140231130	A	C	DENND2A
exm662682	7	140301731	G	T	DENND2A
exm665771	7	142919731	A	C	TAS2R40
rs10216140	7	143016491	A	G	CLCN1
exm666547	7	143175345	T	C	TAS2R41
exm667031	7	143792991	A	G	OR2A12
exm667040	7	143806688	A	C	OR2A2
exm667074	7	143807304	C	T	OR2A2
exm2270709	7	144277770	T	G	TPK1
exm2270710	7	146226582	T	G	CNTNAP2
exm2266489	7	147242624	A	G	MIR548I4
exm2270711	7	147908658	C	T	MIR548T
exm668704	7	149129829	T	C	ZNF777
exm669043	7	149427409	T	G	KRBA1
exm669422	7	149482764	T	C	SSPO
exm670797	7	150217309	T	C	GIMAP7
exm2266492	7	151102867	A	G	WDR86
exm2266496	7	155151194	A	G	BLACE
exm675572	7	156468559	C	T	RNF32
exm676227	7	157929370	T	C	PTPRN2
exm676304	7	157985057	T	C	PTPRN2
exm677906	8	2000428	T	G	MYOM2
exm2273442	8	2795835	T	C	CSMD1
exm-rs1529316	8	3828138	T	C	CSMD1
exm679394	8	6500544	T	C	MCPH1
exm680070	8	8176221	C	T	SGK223
exm680383	8	8560602	T	C	CLDN23
exm-rs9987289	8	9183358	A	G	RP11-115J16.1
exm-rs4841132	8	9183596	A	G	RP11-115J16.1
exm-rs6601299	8	9184691	T	C	RP11-115J16.1
exm-rs2126259	8	9185146	A	G	RP11-115J16.1
exm-rs4335130_ver3	8	10186553	T	C	MSRA

exm2258572	8	10469817	G	A	RP1L1
exm681520	8	10469817	G	A	RP1L1
exm682875	8	11710174	C	T	CTSB
exm683395	8	12878843	A	C	KIAA1456
exm-rs7834383	8	13273477	T	G	DLC1
exm-rs1155204_ver2	8	13334842	G	A	DLC1
exm2258753	8	15599587	G	A	TUSC3
exm684430	8	17396380	A	G	SLC7A2
exm684583	8	17434813	A	G	PDGFRL
exm2258762	8	17548706	G	A	MTUS1
exm685482	8	17918934	A	G	ASAH1
exm686881	8	21847855	C	G	XPO7
exm687345	8	21981218	T	C	HR
exm687855	8	22059363	A	G	BMP1
exm689331	8	23021346	A	G	TNFRSF10D
exm2258787	8	23185977	A	G	LOXL2
exm690789	8	25280800	G	C	GNRH1
exm690955	8	25364331	A	G	CDCA2
rs17426222	8	26717817	T	C	ADRA1A
exm691872	8	27358505	G	A	EPHX2
exm691949	8	27396208	A	G	EPHX2
exm692416	8	27779093	A	G	SCARA5
exm2246842	8	28048668	C	A	ELP3
rs6989655	8	28186972	T	C	PNOC
rs351776	8	28191306	C	A	PNOC
rs2645715	8	28194488	C	T	PNOC
exm692812	8	28304769	T	C	FBXO16
rs7841220	8	31943664	T	C	NRG1-IT1
rs16879599	8	32448869	A	G	NRG1
exm2264286	8	35424695	C	A	UNC5D
exm2262480	8	38369831	A	C	C8orf86
exm696976	8	38854041	T	C	TM2D2
exm2270847	8	38915604	T	G	ADAM9
exm697239	8	39468128	T	A	ADAM18
rs1429948	8	40401035	G	A	ZMAT4
exm699362	8	43152478	A	G	POTEA
exm699964	8	48852225	T	C	PRKDC
exm699990	8	48872671	A	C	PRKDC
exm2266618	8	51287482	G	A	SNTG1

exm701678	8	56686224	T	C	TGS1
exm701736	8	56699352	T	C	TGS1
exm701774	8	56708701	C	T	TGS1
exm701785	8	56711656	A	G	TGS1
exm701819	8	56723557	G	T	TGS1
exm701936_ver2	8	57026229	A	C	MOS
exm701953	8	57078933	T	G	PLAG1
rs2576573	8	57357975	A	G	PENK
rs2609998	8	57360034	T	C	PENK
exm702142	8	57876576	C	T	IMPAD1
rs953177	8	66933620	T	C	DNAJC5B
exm704173	8	67786817	T	C	MCMDC2
exm704421	8	68074137	A	G	CSPP1
exm704684	8	68421768	C	G	CPA6
exm704874	8	69020558	T	C	PREX2
exm705881	8	72975801	T	G	TRPA1
exm-rs7824377	8	72978593	T	C	TRPA1
exm706069	8	73982161	A	G	SBSPON
exm707132	8	79629586	T	C	ZC2HC1A
exm707441	8	81733727	A	C	ZNF704
exm708239	8	87076520	A	C	PSKH2
exm710006	8	95143172	T	G	CDH17
exm2259023	8	95419698	G	A	RAD54B
exm-rs2575735	8	97534651	G	A	SDC2
exm2266659	8	97871757	A	G	CPQ
exm711913	8	99205612	T	C	NIPAL2
exm2259037	8	99208190	C	A	NIPAL2
exm711927	8	99208190	C	A	NIPAL2
exm2271006	8	105084341	C	T	RIMS2
exm714557	8	105361354	C	G	DCSTAMP
exm714594	8	105367121	G	A	DCSTAMP
exm715065	8	107773675	C	T	ABRA
exm715533	8	110413762	C	T	PKHD1L1
exm716189	8	113241088	T	G	CSMD3
exm2258600	8	113470948	G	A	CSMD3
exm2264323	8	119691074	C	A	SAMD12-AS1
exm717279_ver4	8	119964052	G	C	TNFRSF11B
exm2262416	8	120745371	A	G	TAF2
exm717787	8	121061879	A	G	DEPTOR

exm2270914	8	121244089	G	T	COL14A1
exm717924	8	121267490	A	G	COL14A1
exm720581	8	128750540	G	A	MYC
exm722122	8	133984058	T	C	TG
exm722268	8	134108546	T	C	TG
exm722380	8	134237635	T	G	WISP1
exm724323	8	142228909	A	G	SLC45A4
exm2266705	8	143310815	A	G	TSNARE1
exm724845	8	143413136	T	C	TSNARE1
exm724898	8	143427178	G	C	TSNARE1
exm724907	8	143436034	G	A	TSNARE1
exm725145	8	143746069	C	T	JRK
exm2258705	8	143746416	G	A	JRK
exm725485_ver5	8	143867905	T	C	LY6D
exm726599	8	144620584	A	G	ZC3H3
exm727810	8	144808926	T	C	FAM83H
exm728636	8	144942305	T	C	EPPK1
exm730306	8	145059425	C	T	PARP10
exm2266708	8	145581557	A	G	FBXL6
exm731612	8	145603114	A	C	ADCK5
exm732859	8	145729727	A	C	GPT
exm733774	8	145948440	C	T	ZNF251
exm734621	9	215296	G	A	C9orf66
exm735134	9	712156	G	T	KANK1
exm736341	9	3271018	A	G	RFX3
exm-rs301430	9	4576680	C	T	SLC1A1
exm738277	9	6328947	T	C	TPD52L3
exm739092_ver3	9	8518052	C	G	PTPRD
exm2264335	9	9000709	A	C	PTPRD
rs2225979	9	9730381	G	A	PTPRD
exm2259501	9	9770741	C	T	PTPRD
exm-rs10816196	9	9829254	T	G	PTPRD
exm739825	9	14737506	T	G	FREM1
exm739943_ver2	9	14776140	T	C	FREM1
exm-rs10961780	9	14898161	G	T	FREM1
exm2271171	9	16795286	C	T	BNC2
exm740922	9	17273731	G	A	CNTLN
exm742041	9	19346729	T	C	DENND4C
exm742653	9	20953049	G	A	FOCAD

exm742700	9	20988426	A	G	FOCAD
exm2271059	9	21019817	C	T	PTPLAD2
exm742749	9	21029330	C	T	PTPLAD2
exm-rs7023329	9	21816528	A	G	MTAP
exm743465	9	21816758	A	G	MTAP
exm743529	9	21970916	T	C	CDKN2A
exm-rs1011970	9	22062134	T	G	CDKN2B-AS1
exm-rs7045881	9	26935996	T	A	PLAA
exm744113	9	27062721	T	C	IFT74
exm2271064	9	27212966	C	T	TEK
exm2259320	9	32784838	C	T	TMEM215
exm747610	9	34724241	A	C	FAM205A
exm2273455	9	35056961	A	C	VCP
exm750130	9	35826038	C	T	FAM221B
exm750132	9	35826058	C	T	FAM221B
exm750260	9	35870001	T	C	OR13J1
exm750620	9	36170290	A	G	CCIN
exm752006	9	38396502	G	A	ALDH1B1
exm754332	9	73150984	C	T	TRPM3
exm754728	9	74360096	T	C	TMEM2
exm-rs965897	9	77175017	A	C	MIR6130
rs17691363	9	77232494	G	A	RORB
exm-rs11144688	9	78542286	A	G	PCSK5
exm755885	9	78943138	G	T	PCSK5
exm755967	9	79117751	G	A	GCNT1
exm756214	9	79322674	A	G	PRUNE2
exm-rs10781380	9	79408144	C	T	PRUNE2
exm2266915	9	80076247	A	G	GNA14
exm2271101	9	85941646	C	T	FRMD3
rs4486281	9	87455347	G	A	NTRK2
rs10868232	9	87479105	G	A	NTRK2
rs12342902	9	87554440	T	C	NTRK2
rs2586566	9	87570912	C	T	NTRK2
exm758853	9	88651370	C	T	GOLM1
exm759473	9	90500405	A	C	SPATA31E1
exm759536	9	90501448	C	A	SPATA31E1
exm759625	9	90503007	G	A	SPATA31E1
exm760409	9	92003679	T	C	SEMA4D
exm2266922	9	93080194	G	A	RP11-389K14.3

exm761733	9	95396712	A	G	IPPK
exm762182	9	95840256	G	A	SUSD3
exm762395	9	96021312	A	G	WNK2
exm762709_ver3	9	96209943	C	T	FAM120AOS
exm763240	9	97055327	A	G	ZNF169
exm2266852	9	97221434	G	A	HIATL1
exm764250	9	98691137	C	T	ERCC6L2
exm2266856	9	100307493	A	G	TMOD1
exm765583	9	100388119	T	G	TSTD2
exm765802	9	100684757	G	A	C9orf156
exm2259043	9	100689693	G	A	HEMGN
exm-rs755109	9	100696203	C	T	HEMGN
exm765914	9	100823135	C	G	NANS
exm2259049	9	101350077	C	A	GABBR2
exm766657	9	101748234	A	G	COL15A1
exm2247322	9	105779244	G	A	CYLC2
exm769038	9	107562804	T	C	ABCA1
exm2262569	9	107571241	C	T	ABCA1
exm769591	9	109687403	G	A	ZNF462
exm769678	9	109689752	T	C	ZNF462
exm770341	9	111685129	T	A	IKBKAP
exm771456	9	113169631	A	G	SVEP1
exm2271132_ver2	9	115155264	C	T	HSDL2
exm773531	9	116060221	T	C	RNF183
exm773653	9	116132334	T	C	BSPRY
exm774531	9	116930100	A	G	COL27A1
exm774538	9	116930194	G	A	COL27A1
exm774606_ver2	9	116931099	A	G	COL27A1
exm774631	9	116931445	C	T	COL27A1
exm775355	9	117166206	T	G	DFNB31
exm775453	9	117186712	T	C	DFNB31
exm775635	9	117554791	T	C	TNFSF15
exm776143	9	118163563	C	T	42705
exm779273	9	125377734	G	A	OR1Q1
exm779371	9	125424277	G	A	OR1L1
exm2266873	9	125585909	G	A	PDCL
exm-rs2479106	9	126525212	G	A	DENND1A
exm-rs1549314	9	127910307	G	T	PPP6C
exm781484	9	127975654	T	C	RABEPK

exm2271152	9	130576075	C	T	FPGS
exm784337	9	130884753	T	C	PTGES2
exm787627	9	132084621	A	G	C9orf106
exm787639	9	132374678	C	T	C9orf50
exm787682	9	132377900	T	C	C9orf50
exm2259186	9	133324400	G	A	ASS1
exm789485	9	133954548	T	C	LAMC3
exm790420	9	134385436	A	G	POMT1
exm790437	9	134386781	A	G	POMT1
exm790561	9	134401335	C	T	UCK1
exm791448	9	135413085	C	A	C9orf171
exm-rs1076160	9	135776034	A	G	TSC1
exm792745	9	136135238	T	C	ABO
exm792747	9	136136770	A	C	ABO
exm2271237	9	136279935	T	C	REXO4
rs1108581	9	136505241	G	A	DBH
rs1611124	9	136509275	T	G	DBH
exm793979	9	136509370	T	G	DBH
rs4531	9	136509370	T	G	DBH
rs2797853	9	136512515	A	G	DBH
rs77905	9	136518097	C	T	DBH
rs129883	9	136524811	G	C	DBH
rs590614	9	136635409	T	C	VAV2
exm795343	9	137779026	T	C	FCN2
exm795986	9	138439806	T	C	OBP2A
exm795988	9	138439809	C	T	OBP2A
exm795994	9	138440554	C	G	OBP2A
exm795995	9	138440562	T	C	OBP2A
exm797041	9	139110654	T	C	QSOX2
exm798308	9	139368953	A	G	SEC16A
exm802443	9	140110555	A	G	NDOR1
exm803264	9	140250818	C	T	EXD3
exm803286	9	140267490	A	G	EXD3
exm803861	9	140409877	A	C	PNPLA7
exm803863	9	140409891	C	G	PNPLA7
exm805536	10	1060218	A	G	GTPBP4
exm2249023	10	1066710	T	C	IDI2
exm806531	10	4889403	T	C	AKR1E2
exm806694	10	5136651	G	C	AKR1C3

exm807131	10	5541230	C	T	CALML5
exm2266954	10	5705900	G	A	ASB13
exm807672	10	5929962	C	G	ANKRD16
exm808180	10	6527143	A	G	PRKCQ
exm808535	10	7622009	A	G	ITIH5
exm808756	10	7780646	G	C	ITIH2
exm-rs501764	10	8093034	G	T	GATA3-AS1
exm809345	10	11797500	A	G	ECHDC3
exm810992	10	15008493	A	C	MEIG1
rs4748184	10	15643998	G	A	ITGA8
exm811504	10	15649698	G	T	ITGA8
exm812223	10	17024503	G	A	CUBN
exm812431	10	17156151	A	G	CUBN
exm2271259	10	18511082	C	T	CACNB2
exm813332	10	18964097	A	G	ARL5B
exm2259564	10	19705383	C	A	MALRD1
exm813479	10	20506418	G	A	PLXDC2
exm-rs7083165	10	20516587	C	T	PLXDC2
exm813491	10	20534333	A	G	PLXDC2
exm-rs1571942	10	20542634	C	T	PLXDC2
exm2273462	10	21074455	C	T	NEBL
exm814083	10	22605398	G	C	COMMD3,COMMD3-BMI1
exm814183	10	22675857	G	A	SPAG6
exm814301	10	23250826	A	G	ARMC3
exm814365	10	23297252	A	G	ARMC3
exm2249163	10	24489659	G	A	KIAA1217
exm-rs11013962	10	24495586	G	A	KIAA1217
exm2249177	10	28228865	A	G	ARMC4
exm817456	10	29784026	G	A	SVIL
exm-rs2505083	10	30335122	C	T	KIAA1462
exm819812	10	37488689	G	C	ANKRD30A
exm-rs1148259	10	37508450	G	T	ANKRD30A
exm2259592	10	43014780	T	C	ZNF37BP
exm820373	10	43089965	G	A	ZNF33B
exm-rs2742234	10	43612609	C	T	RET
exm821333	10	45478092	G	A	RASSF4
exm2271286	10	49810977	T	G	ARHGAP22
exm824042_ver2	10	50678317	G	C	ERCC6
exm824258	10	50732280	T	C	ERCC6,ERCC6-

					PGBD3
exm-rs10993994	10	51549496	T	C	MSMB
exm2266994	10	52103707	A	G	SGMS1
rs10490964	10	52256902	T	C	SGMS1
exm2266995	10	53657085	A	G	PRKG1
exm2249294	10	60118681	C	T	UBE2D1
exm2266998	10	60548276	A	G	BICC1
exm826442	10	61083752	A	G	FAM13C
exm826529	10	61552692	T	G	CCDC6
rs10761454	10	61870135	G	A	ANK3
rs12779035	10	61876720	T	C	ANK3
rs10994210	10	61878214	T	C	ANK3
rs10509125	10	61926866	C	A	ANK3
rs11814752	10	61954250	A	G	ANK3
rs11815504	10	62028144	C	A	ANK3
exm2267000	10	63962026	G	A	RTKN2
exm2267005	10	67894481	A	G	CTNNA3
exm829637	10	70716061	T	C	DDX21
exm-rs1227756_ver2	10	71588504	A	G	COL13A1
exm831569	10	72500763	T	C	ADAMTS14
exm831669	10	72517830	A	G	ADAMTS14
exm832239	10	73199595	T	C	CDH23
exm832552	10	73498355	A	G	CDH23
rs4148926	10	73737124	C	G	CHST3
rs4747229	10	73747591	T	C	CHST3
exm833761	10	74886544	G	A	NUDT13
exm2271314_ver4	10	75409967	T	C	SYNPO2L
exm2273464	10	75562108	A	G	NDST2
exm-rs2116830	10	78646536	A	C	KCNMA1
exm2259638	10	79026227	T	C	KCNMA1
exm837508_ver4	10	81926702	A	G	ANXA11
exm838161	10	85972932	G	A	CDHR1
exm838949	10	88422116	C	T	OPN4
exm-rs10887741	10	89443310	C	T	PAPSS2
exm840196	10	90580238	T	C	LIPM
exm840200	10	90582744	A	C	ANKRD22
rs4934429	10	90679053	A	G	STAMBPL1
exm840462	10	91007339	T	C	LIPA
exm840906	10	91405044	T	C	PANK1

exm841053	10	91497631	A	T	KIF20B
exm841092	10	91498254	G	A	KIF20B
exm-rs1592051	10	93542186	T	C	TNKS2-AS1
exm842230	10	94695617	C	T	EXOC6
exm842801	10	95259983	T	C	CEP55
exm842845	10	95279506	A	T	CEP55
exm843013	10	95381773	A	T	PDE6C
exm-rs2785137	10	95386207	A	G	PDE6C
exm843481	10	96058298	T	C	PLCE1
exm843494	10	96066341	G	A	PLCE1
exm-rs3781264	10	96070375	C	T	PLCE1
exm843546	10	96098373	T	C	NOC3L
exm-rs1799853	10	96702047	T	C	CYP2C9
exm-rs4086116	10	96707202	T	C	CYP2C9
exm844097	10	96798749	C	T	CYP2C8
exm844571	10	97386497	T	G	ALDH18A1
exm844653	10	97445357	A	C	TCTN3
exm844771	10	97687049	C	G	C10orf131
exm845579	10	98469693	T	C	PIK3AP1
exm846214_ver4	10	99125924	T	C	RRP12
exm846642	10	99225846	T	C	MMS19
exm847208	10	99625319	T	C	CRTAC1
exm-rs11599750	10	101805442	T	C	CPN1
exm2271342	10	103662789	C	T	C10orf76
exm854148	10	105810400	T	C	COL17A1
exm854519	10	105957714	G	A	WDR96
exm854831	10	106152111	A	T	CCDC147
exm855038	10	107022160	A	G	SORCS3
exm856238	10	114427978	A	G	VTI1A
exm856586	10	115370274	C	T	NRAP
exm856724	10	115393929	A	G	NRAP
exm856771	10	115410234	C	T	NRAP
exm857795	10	116100461	T	C	AFAP1L2
exm858786	10	118439206	C	T	HSPA12A
rs363231	10	119032488	A	G	SLC18A2
exm859114	10	119043554	T	C	PDZD8
rs2912791	10	123318961	T	C	FGFR2
rs1219639	10	123358312	A	G	FGFR2
exm-rs10510102	10	123625190	G	A	ATE1

exm863434	10	126715140	C	G	CTBP2
exm-rs9422917_ver4	10	127352628	T	C	TEX36
exm2271441	10	128944080	C	T	DOCK1,FAM196A
exm2267066	10	132992902	A	G	TCERG1L
exm866646	10	134459388	G	A	INPP5A
exm867489	10	135000159	G	A	KNDC1
exm868455	10	135184126	G	A	ECHS1
exm2249125	10	135215667	T	C	MTG1
rs915906	10	135343738	C	T	CYP2E1
rs743535	10	135349367	T	C	CYP2E1
rs2515642	10	135352013	C	T	CYP2E1
exm868815	10	135368590	C	T	SYCE1
exm868850	10	135369532	C	T	SYCE1
exm868865	10	135370639	A	C	SYCE1
exm870211	11	376331	A	T	B4GALNT4
exm870534	11	408174	T	G	SIGIRR
exm870688	11	430339	G	C	ANO9
exm870729	11	433867	A	G	ANO9
exm871093	11	554047	A	G	LRRC56
exm873086	11	830044	A	G	EFCAB4A
exm874845_ver2	11	1094761	A	C	MUC2
exm876605	11	1276327	A	G	MUC5B
exm876673	11	1280238	A	G	MUC5B
exm2271450	11	1301688	C	T	TOLLIP
exm877588	11	1857751	G	C	SYT8
exm877634	11	1858632	T	C	SYT8
exm878483	11	2424647	A	G	TSSC4
exm2267247	11	2473131	G	A	KCNQ1
exm-rs2237892	11	2839751	T	C	KCNQ1
exm879019	11	2924591	A	G	SLC22A18
exm-rs16928809	11	2936952	A	G	SLC22A18
exm879746_ver3	11	3659993	T	G	ART5
exm881219	11	4673788	A	G	OR51E1
exm881914	11	5010905	G	A	MMP26
exm882638	11	5372751	C	A	OR51B6
exm882659	11	5373104	G	A	OR51B6
exm882660	11	5373111	A	G	OR51B6
exm882661_ver4	11	5373114	G	A	OR51B6
exm882663	11	5373129	T	C	OR51B6

exm882666	11	5373170	G	C	OR51B6
exm882672	11	5373242	G	T	OR51B6
exm882673	11	5373251	T	C	OR51B6
exm882675_ver3	11	5373311	C	T	OR51B6
exm882689	11	5373562	A	C	OR51B6
exm882947	11	5475118	G	A	OR51I2
exm883218	11	5537245	T	G	UBQLNL
exm883318_ver2	11	5602438	T	A	OR52B6
exm883327_ver3	11	5602615	A	G	OR52B6
exm883771	11	5776287	C	T	OR52N4
exm883784	11	5776470	T	G	OR52N4
exm883931	11	5841701	A	G	OR52N2
exm883966	11	5842310	T	G	OR52N2
exm883969	11	5842329	G	T	OR52N2
exm883975	11	5842384	G	C	OR52N2
exm884460	11	6129837	T	C	OR56B4
rs906895	11	6280248	G	A	CCKBR
exm2250126	11	6412931	C	T	SMPD1
exm2250179_ver3	11	6643976	T	C	DCHS1
exm886450	11	6647875	G	C	DCHS1
exm2271581	11	7657821	G	T	PPFIBP2
exm888277	11	7846599	C	T	OR5P3
exm889161	11	8751640	C	G	ST5
exm889271	11	8947021	A	G	C11orf16
exm890147	11	9861208	C	G	SBF2
exm890344	11	10503736	T	C	AMPD3
exm2273473	11	10536298	C	T	RNF141
exm890990	11	10875055	C	T	ZBED5
exm891143_ver2	11	11906050	C	G	USP47
exm2267151	11	14233522	A	G	SPON1
exm892077	11	14264916	A	G	SPON1
exm2267154	11	16274295	A	G	SOX6
exm895787_ver2	11	19955194	C	G	NAV2
exm-rs7111546	11	22829757	T	C	GAS2
exm-rs4561213_ver2	11	24678819	T	G	LUZP2
exm-rs10500991	11	24849566	T	C	LUZP2
exm897426	11	27389739	C	T	LGR4
rs972096	11	27552382	T	C	BDNF-AS
exm-rs988712	11	27563382	T	G	BDNF-AS

rs11030101	11	27680744	T	A	BDNF-AS
rs10835210	11	27695910	A	C	BDNF-AS
exm898035	11	30974115	A	C	DCDC5
exm898121	11	31329373	T	C	DCDC1
exm898618	11	32874926	A	G	PRRG4
exm-rs178523	11	44097226	G	A	ACCS
exm-rs11605924	11	45873091	C	A	CRY2
exm-rs7945565	11	45878992	G	A	CRY2
rs2292910	11	45903613	A	C	CRY2
exm904813	11	47306630	T	C	MADD
exm905389	11	47469654	G	A	RAPSN
exm905915	11	47776156	C	T	FNBP4
exm906233	11	48158586	A	T	PTPRJ
exm907069	11	51411740	T	A	OR4A5
exm907418	11	55135435	T	C	OR4A15
exm907592	11	55339652	T	C	OR4C16
exm907665	11	55340379	T	C	OR4C16
exm907891	11	55433040	C	T	OR4C6
exm908210	11	55606693	A	G	OR5D16
exm908326	11	55681149	C	G	OR5W2
exm908339	11	55681416	G	A	OR5W2
exm908354	11	55681580	G	A	OR5W2
exm909231	11	56086147	T	G	OR8K3
exm909274	11	56113516	T	C	OR8K1
exm909353	11	56128081	G	A	OR8J1
exm909612_ver4	11	56230120	A	G	OR5M9
exm909627	11	56230328	T	G	OR5M9
exm909649	11	56230678	A	G	OR5M9
exm910187	11	56510623	G	A	OR9G4
exm910232	11	56511160	G	A	OR9G4
exm910257	11	56756664	G	A	OR5AK2
exm910425_ver2	11	57069375	G	A	TNKS1BP1
exm910431	11	57069568	C	T	TNKS1BP1
exm910599	11	57080352	A	G	TNKS1BP1
exm2250013	11	57080917	A	G	TNKS1BP1
exm2218834	11	57103350	G	A	SSRP1
exm913217_ver2	11	58978940	T	C	MPEG1
exm914021	11	59612859	C	T	GIF
exm914045	11	59623354	T	C	TCN1

exm914648	11	60291413	C	T	MS4A13
exm915553	11	60892606	A	G	CD5
exm915580	11	60899767	A	G	VPS37C
exm916288	11	61252200	G	A	PPP1R32
exm916315	11	61253841	A	C	PPP1R32
exm916677	11	61548796	A	G	MYRF
exm919931	11	62606707	C	T	WDR74
rs12282281	11	63168015	G	A	MIR3680-1,MIR3680-2
exm920637	11	63231042	A	G	HRASLS5
exm922500	11	64109118	G	T	CCDC88B
exm-rs17300741	11	64331462	G	A	SLC22A11
exm-rs2078267	11	64334114	A	G	SLC22A11
exm2219165_ver2	11	64902002	A	C	SYVN1
exm926851	11	65319751	A	G	LTBP3
exm-rs3782089	11	65336819	A	G	SSSCA1-AS1
exm-rs2242663	11	66335308	G	A	CTSF
exm2250186	11	66610645	C	G	C11orf80
exm2267187	11	66832528	G	A	RHOD
exm2264428	11	67867548	C	A	CHKA
exm934866	11	68030015	T	C	C11orf24
exm935380	11	68512543	G	A	MTL5
exm936004	11	68840160	G	A	TPCN2
exm937126	11	70666765	T	C	SHANK2
exm940765	11	74047743	A	T	PGM2L1
rs528833	11	74981039	A	G	ARRB1
exm941891	11	75298468	C	A	MAP6
exm942709	11	76637651	G	A	ACER3
exm2250298	11	76701606	G	A	ACER3
exm942770	11	76751166	A	G	B3GNT6
exm2267195	11	77746118	G	A	KCTD14,NDUFC2-KCTD14
exm2271625	11	77882393	C	T	KCTD21
exm944242	11	77921527	G	C	USP35
exm945129	11	82877492	T	C	PCF11
exm-rs3885683	11	83455707	G	A	DLG2
exm946243	11	86158235	T	C	ME3
exm946261	11	86161388	G	C	ME3
exm2267208	11	90646272	A	G	DISC1FP1
exm-rs2248020	11	93259965	A	C	SMCO4
exm948143	11	93408743	A	G	KIAA1731

exm948748	11	93913036	G	A	PANX1
exm949768	11	95712897	T	G	MAML2
exm950156	11	100792335	A	G	ARHGAP42
exm951049	11	102562700	T	C	MMP27
exm951505	11	102713620	C	T	MMP3
exm-rs10895547	11	103808152	T	C	PDGFD
exm952321	11	104825597	T	C	CASP4
exm955176	11	111608258	G	C	PPP2R1B
exm955488_ver2	11	111795085	G	C	C11orf52
rs10891503	11	112964139	C	T	NCAM1
exm956325	11	113269792	A	C	ANKK1
rs2075652	11	113294898	T	C	DRD2
rs17601612	11	113317745	C	G	DRD2
rs11214763	11	113774895	A	G	HTR3B
rs17116121	11	113801668	A	G	HTR3B
rs17116124	11	113802435	C	T	HTR3B
exm956846	11	113803666	A	G	HTR3B
rs17116138	11	113803666	A	G	HTR3B
rs11214775	11	113807181	A	G	HTR3B
rs3782025	11	113807607	C	T	HTR3B
rs1176761	11	113813613	T	A	HTR3B
rs7945926	11	113817468	T	C	HTR3B
rs11604247	11	113846904	T	C	HTR3A
rs10891611	11	113851413	C	T	HTR3A
rs11607240	11	113851853	T	C	HTR3A
rs11214796	11	113854679	C	T	HTR3A
rs10160548	11	113856681	G	T	HTR3A
rs1176713	11	113860425	C	T	HTR3A
exm-rs28927680	11	116619073	C	G	BUD13
exm957556	11	116633862	A	G	BUD13
exm-rs2075290	11	116653296	C	T	ZNF259
exm957617	11	116655600	A	G	ZNF259
exm957713	11	116662407	C	G	APOA5
exm2271563	11	117436190	T	C	DSCAML1
exm959438	11	117965530	G	A	TMPRSS4
exm2249596	11	118070264	G	A	AMICA1
exm959627	11	118074337	A	G	AMICA1
exm959643	11	118081345	T	A	AMICA1
exm963001	11	119243670	T	C	USP2

exm963087	11	119509485	A	G	PVRL1
exm963327	11	120099679	G	A	OAF
exm2271650	11	120142118	C	T	POU2F3
exm-rs11217785	11	120152591	T	C	POU2F3
exm965064	11	122955402	T	C	CLMP
exm965389	11	123753958	T	C	TMEM225
exm965539	11	123813795	C	T	OR6T1
exm965581	11	123814356	A	G	OR6T1
exm965591	11	123814478	G	A	OR6T1
exm966148_ver3	11	124096292	C	A	OR8G2
exm966366	11	124252963	T	A	OR8B2
exm966884	11	124618346	G	A	VSIG2
exm967435	11	124789828	T	A	HEPN1
exm967557	11	124857708	A	G	CCDC15
exm967612	11	124908352	C	T	CCDC15
exm968179	11	125708293	G	T	PATE4
exm968506	11	125889526	T	C	CDON
exm-rs4935969	11	126296589	T	C	KIRREL3
rs7303797	12	342911	A	G	SLC6A13
rs10848623	12	359463	G	A	SLC6A13
exm973530	12	999638	T	C	WNK1
exm973603	12	1023218	T	G	RAD52
exm974189	12	1995403	T	C	CACNA2D4
rs4765670	12	2313126	G	A	CACNA1C
rs10848635	12	2316195	A	T	CACNA1C
rs10848637	12	2316554	A	C	CACNA1C
rs10848645	12	2420244	A	G	CACNA1C
rs10774037	12	2420526	G	A	CACNA1C
rs1015287	12	2605386	T	C	CACNA1C
rs11062261	12	2647941	A	C	CACNA1C
exm975144	12	3147203	T	C	TEAD4
exm976878	12	6173433	T	C	VWF
exm978072	12	6675322	A	G	NOP2
exm2251158	12	6939859	T	C	LEPREL2
exm978975	12	6940492	A	G	LEPREL2
exm979090	12	6954864	A	G	GNB3
exm980178	12	7281317	T	C	RBP5
exm2267317	12	7309199	G	A	CLSTN3
exm980946	12	7842587	G	C	GDF3

exm981752	12	8667897	G	A	CLEC4D
exm982156	12	9020489	A	G	A2ML1
exm-rs4763879	12	9910164	A	G	CD69
exm984033	12	10978402	G	A	TAS2R10
exm984299	12	11150054	T	C	TAS2R20
rs890	12	13715308	G	T	GRIN2B
rs1805199	12	13769664	A	G	GRIN2B
rs220590	12	13964967	A	G	GRIN2B
rs220597	12	13968186	G	A	GRIN2B
rs219904	12	14092888	T	C	GRIN2B
exm986697	12	14587301	G	A	ATF7IP
exm-rs2900333	12	14653867	T	C	ATF7IP
exm986797	12	14656768	G	C	PLBD1
exm987643	12	15800074	A	G	EPS8
exm987828	12	16397734	A	C	SLC15A5
exm2271691	12	16400613	T	C	SLC15A5
exm987962	12	18435452	T	C	PIK3C2G
exm-rs1348582	12	20531756	C	T	PDE3A
exm2250767	12	20605116	A	G	PDE3A
exm-rs10771012	12	23788075	G	A	SOX5
exm2250795	12	23923719	T	C	SOX5
exm990271	12	25242595	C	G	LRMP
exm990409	12	25311489	T	G	CASC1
exm991126	12	27234999	C	T	C12orf71
exm991356	12	27825403	C	T	PPFIBP1
exm991451	12	27850113	T	A	REP15
exm991473	12	27867727	A	G	MRPS35
exm991519_ver3	12	27916206	A	G	MANSC4
exm991520_ver3	12	27916224	G	A	MANSC4
exm2220403	12	28122893	G	A	PTHLH
exm991802	12	29598292	A	G	OVCH1
exm991855_ver2	12	29630081	G	A	OVCH1
exm992509	12	31249861	G	C	DDX11
exm992858	12	31648826	T	C	DENND5B
exm993000	12	32134815	A	G	KIAA1551
exm-rs10844154	12	32380501	A	C	BICD1
exm-rs708224_ver2	12	32436409	A	G	BICD1
exm993673	12	33021934	G	A	PKP2
exm994080	12	39713776	A	C	KIF21A

exm-rs1491942	12	40620808	C	G	LRRK2
exm995828	12	44148259	C	T	PUS7L
exm995907	12	44177511	A	G	IRAK4
exm2271721	12	44932106	T	C	NELL2
exm997329	12	48142636	T	C	RAPGEF3
rs10875745	12	48515405	A	T	PFKM
exm998105	12	48526712	A	G	PFKM
exm998517	12	48920006	A	G	OR8S1
exm998566	12	49047912	T	C	KANSL2
exm1002971	12	51457854	A	G	CSRNP2
exm1003151	12	51685707	C	T	BIN2
exm1004274	12	52636874	T	C	KRT7
exm1004876	12	52788928	A	G	KRT82
exm1005818	12	52966249	A	T	KRT74
exm1009106	12	54109659	T	C	CALCOCO1
exm-rs7962801	12	55346235	T	C	TESPA1
exm1010414	12	55615229	C	T	OR10A7
exm1013486	12	56814653	A	G	TIMELESS
rs2291738	12	56815281	G	A	TIMELESS
exm1013519_ver2	12	56815922	T	C	TIMELESS
rs774026	12	56821998	G	A	TIMELESS
exm1013587	12	56822378	A	T	TIMELESS
rs7302060	12	56829092	C	T	TIMELESS
rs10876890	12	56833751	A	T	TIMELESS
exm-rs2958154	12	57065713	G	A	PTGES3
exm1014122	12	57109931	A	T	NACA
exm1014340	12	57114307	A	T	NACA
exm1014414	12	57146069	G	T	PRIM1
exm1014783	12	57422934	A	G	MYO1A
exm-rs11172113	12	57527283	C	T	LRP1
exm1015309	12	57567762	T	C	LRP1
exm2267354	12	57616013	G	A	NXPH4
exm1016018	12	57648644	T	C	R3HDM2
exm1016127	12	57843711	A	G	INHBC
exm2264475	12	70728679	A	C	CNOT2
exm1021538	12	71078528	T	C	PTPRR
rs11179003	12	72343287	T	C	TPH2
exm-rs10506701	12	74586210	C	A	RP11-81H3.2
exm1022530	12	75693691	T	C	CAPS2

exm1023213	12	78225374	A	G	NAV3
exm1023955	12	80761430	G	A	OTOGL
exm1023970	12	80765800	A	G	OTOGL
exm1024008	12	80878317	A	G	PTPRQ
exm2267370	12	83413964	G	A	TMTC2
exm1024715	12	85277561	A	T	SLC6A15
exm1025441	12	88500847	C	T	CEP290
exm1025495	12	88523494	G	C	CEP290
exm2260140	12	91380633	A	G	EPYC
exm-rs1836127	12	93550301	T	G	RP11-486A14.2
exm2271774	12	93751933	C	T	RP11-486A14.2
exm1026625	12	94603406	C	G	PLXNC1
exm1026843	12	94772742	T	C	CCDC41
exm2271860	12	95002363	T	C	TMCC3
exm1027623	12	96292170	G	C	CCDC38
exm1027775	12	96374614	C	T	HAL
exm2267464	12	99586180	A	G	ANKS1B
exm-rs7968606	12	99816842	T	C	ANKS1B
exm1029708	12	101587518	T	C	SLC5A8
exm2267388	12	105606172	G	A	APPL2
exm1033000	12	106848414	G	A	POLR3B
exm-rs10861661	12	107174646	C	A	RIC8B
exm1033906	12	108912178	A	G	FICD
exm1034488	12	109221123	A	G	SSH1
exm2271791_ver3	12	109278747	G	T	DAO
rs11114084	12	109281695	A	G	DAO
rs11114085	12	109282508	C	T	DAO
rs10861974	12	109282685	T	C	DAO
exm-rs11114086	12	109286399	T	C	DAO
exm1034656	12	109520769	G	A	USP30
exm1034882	12	109617728	G	A	ACACB
exm2250573	12	109617865	T	C	ACACB
exm2259957	12	110196599	T	C	FAM222A-AS1
exm1036408	12	110566907	G	A	IFT81
exm-rs10849915	12	111333622	C	T	CCDC63
exm-rs10774610	12	111340243	C	T	CCDC63
exm2271798	12	113325629	C	T	RPH3A
exm1040225	12	113874590	A	G	SDSL
rs3782221	12	117795881	A	G	NOS1

rs435136	12	120251380	T	C	CIT
exm1042560	12	120313953	T	C	CIT
exm1043475_ver2	12	120954490	T	C	COQ5
exm1043912	12	121416650	C	A	HNF1A
rs500930	12	121573997	T	C	P2RX7
rs17434731	12	121583993	T	C	P2RX7
rs6489794	12	121597413	A	G	P2RX7
exm1044252	12	121615103	A	G	P2RX7
rs11065468	12	121615369	C	T	P2RX7
exm2267400	12	121715395	A	G	CAMKK2
exm1048587	12	124242557	G	A	ATP6V0A2
exm1048846	12	124325977	T	G	DNAH10
exm1049411	12	124798811	A	G	FAM101A,ZNF664-FAM101A
exm1049596	12	124826462	T	C	NCOR2
exm1049762	12	124856830	T	C	NCOR2
exm2250670	12	125318772	T	C	SCARB1
exm-rs4765623	12	125320850	T	C	SCARB1
exm1050298	12	125453115	T	C	DHX37
exm1050990	12	129559421	T	C	TMEM132D
exm-rs885389	12	131621762	A	G	GPR133
exm1052171	12	132335592	A	G	MMP17
exm1053169	12	132626425	A	G	DDX51
exm1054122	12	133295353	T	C	PGAM5
exm2271816	12	133498358	T	G	ZNF605
exm1054917	12	133778796	T	C	ZNF268
exm1055155	13	20000630	T	C	TPTE2
exm1055304	13	20224202	T	G	MPHOSPH8
exm1055846	13	21189941	A	G	IFT88
exm2273503	13	22275946	T	A	FGF9
exm-rs9318086	13	24432467	A	G	MIPEP
exm2277048	13	24895617	C	T	C1QTNF9
exm1058110	13	25267017	C	A	ATP12A
exm1058936	13	25887811	C	T	NUPL1
exm-rs4771122_ver2	13	28020180	G	A	MTIF3
exm-rs7097	13	28197436	A	G	POLR1D
exm2267589	13	28426279	A	G	PDX1-AS1
exm2267504	13	28953808	A	G	FLT1
exm2267593	13	34491838	A	G	RFC3
exm1063885	13	38138689	T	C	POSTN

exm1065144	13	41323397	T	C	MRPS31
exm1065270	13	41515118	T	C	ELF1
rs943390	13	42636943	A	G	DGKH
rs1033950	13	42651603	G	A	DGKH
exm-rs1012053	13	42653437	C	A	DGKH
rs2593116	13	42664452	A	C	DGKH
rs1170195	13	42668685	C	T	DGKH
rs1170191	13	42675493	T	C	DGKH
exm2273507	13	42803341	T	C	DGKH
exm1067872	13	46946157	T	C	KIAA0226L
exm1068096_ver3	13	47469968	T	G	HTR2A
rs1805055	13	47469968	A	C	HTR2A
exm1069048	13	50141345	G	A	RCBTB1
exm1069146	13	50306528	C	T	KPNA3
exm1069269	13	50587140	C	G	TRIM13
exm1069741_ver3	13	52365354	T	G	DHRS12
exm1070404	13	52952024	A	C	THSD1
exm1070439	13	52952634	G	C	THSD1
exm1070468	13	52971718	C	G	THSD1
exm-rs11838918	13	79410574	C	T	LINC00331
exm2271978	13	96190232	G	T	CLDN10
exm1074942	13	96239805	A	G	DZIP1
exm1075110	13	96506596	C	T	UGGT2
exm1075180	13	96540204	G	T	UGGT2
exm2251288	13	100623882	A	G	ZIC5
exm1077499	13	103343230	G	C	METTL21C
exm1078162	13	103513944	G	A	BIVM-ERCC5,ERCC5
exm2267625	13	108136716	A	G	FAM155A
exm1080133	13	111532381	A	G	ANKRD10
rs17120218	13	113164017	C	T	TUBGCP3
exm1080722	13	113479813	A	G	ATP11A
exm2251325	13	113479813	A	G	ATP11A
exm1080723	13	113479820	A	G	ATP11A
exm2271997	13	114830132	T	C	RASA3
exm1084123	14	20692188	C	T	OR11H6
exm2251692	14	20692291	T	C	OR11H6
exm1084138_ver2	14	20692453	T	G	OR11H6
exm1084150	14	20692643	C	T	OR11H6
exm1084549	14	20846950	T	C	TEP1

exm1084657	14	20852267	T	C	TEP1
exm1085301	14	21109745	G	C	OR6S1
exm1085495_ver3	14	21360216	C	G	RNASE3
exm1087120	14	21993498	G	A	SALL2
exm1087185	14	22038644	C	T	OR10G3
exm1089467	14	23512041	A	G	PSMB11
exm1090204	14	23829164	A	G	EFS
exm1090216	14	23829490	T	C	EFS
exm1093406	14	24760764	A	G	DHRS1
exm1094584	14	25043671	C	T	CTSG
exm1095391	14	31583512	T	C	HECTD1
exm2267702	14	37603194	A	G	SLC25A21
exm1097659	14	38061547	C	G	FOXA1
exm2251779_ver2	14	39241025	C	T	LINC00639
exm2272166	14	41522215	G	T	LOC644919
exm1098541	14	44974966	A	G	FSCB
exm1098549_ver4	14	44975052	G	A	FSCB
exm1098595	14	44975606	T	G	FSCB
exm1099056	14	45636328	G	A	FANCM
exm1099301	14	45700409	T	C	MIS18BP1
rs1954232	14	47417588	T	C	MDGA2
rs10134041	14	47437326	T	C	MDGA2
exm1100606	14	50901768	A	G	MAP4K5
exm1101329	14	51446216	T	G	TRIM9
exm1101699	14	52496387	C	A	NID2
exm1101752	14	52509501	C	T	NID2
exm-rs730532	14	52518811	C	T	NID2
exm1101767	14	52520368	C	T	NID2
exm1102004	14	52937255	T	C	TXNDC16
exm2251834	14	53023607	A	G	GPR137C
exm1102109	14	53098902	G	A	GPR137C
exm1105033	14	59939804	A	G	L3HYPDH
exm1105139	14	59988324	T	C	CCDC175
exm1105159	14	60027907	T	C	CCDC175
exm1105610	14	60932752	A	G	C14orf39
exm1105655	14	60976537	C	A	SIX6
exm2272092	14	61443539	C	T	TRMT5
exm1106720	14	64447776	C	T	SYNE2
exm1106776	14	64467358	C	A	SYNE2

exm1106901	14	64497894	A	C	SYNE2
exm1106902	14	64497929	G	A	SYNE2
exm1107251	14	64596823	A	C	SYNE2
rs3784075	14	67291186	C	T	GPHN
rs10143670	14	67319524	G	A	GPHN
rs10147356	14	67431696	G	A	GPHN
rs9323489	14	67484346	G	A	GPHN
rs10144371	14	67604637	G	T	GPHN
exm-rs8017304	14	68785077	G	A	RAD51B
exm2272180	14	68851441	C	T	RAD51B
exm1111399	14	70924517	A	G	ADAM21
exm2267720	14	72022891	G	A	SIPA1L1
exm2272109	14	72467631	G	T	RGS6
exm-rs4140998_ver2	14	72895359	A	G	RGS6
exm2251917	14	73440864	T	C	ZFYVE1
exm1113028	14	73727509	T	G	PAPLN
exm1113298	14	73958812	C	T	C14orf169
exm1114477	14	74759006	T	C	ABCD4
exm1115257_ver4	14	75230953	T	C	YLPM1
exm1116310	14	76045362	C	T	FLVCR2
exm2273521	14	77978621	C	A	SPTLC2
exm2272130	14	89311519	G	T	TTC8
exm1121075	14	91739081	A	G	CCDC88C
exm2272191	14	92987358	T	C	RIN3
exm1122363	14	93118038	A	G	RIN3
exm1122576	14	93199080	T	C	LGMN
exm1122589	14	93263982	G	C	GOLGA5
exm1123584	14	94417531	C	T	ASB2
exm1125198	14	95884323	G	A	SYNE3
exm1126214	14	99182559	A	G	C14orf177
exm1128732	14	102729881	G	A	MOK
exm1128758_ver4	14	102792386	C	T	ZNF839
exm1128771	14	102792631	A	G	ZNF839
exm1128849_ver4	14	102808157	A	G	ZNF839
exm1128880	14	102815042	T	C	CINP
exm1128949	14	102894593	G	A	TECPR2
exm1128959	14	102898204	A	G	TECPR2
exm1129005	14	102901201	G	A	TECPR2
exm1130291	14	104166994	T	C	KLC1

exm1132199	14	105406372	T	C	AHNAK2
exm1133074	14	105414790	A	G	AHNAK2
exm1133981	14	105420134	T	C	AHNAK2
exm1134389	14	105615648	T	C	JAG2
exm1134396	14	105617042	C	T	JAG2
exm1134928	14	105930406	A	G	MTA1
exm-rs3867498	15	25078787	T	C	SNRPN
exm1142590	15	25926179	T	C	ATP10A
exm1144533	15	31221493	T	C	FAN1
exm1144579	15	31234064	T	C	MTMR10
exm2260416	15	32347464	T	C	CHRNA7
exm2272222	15	32396457	T	C	CHRNA7
exm1145363	15	33261185	A	G	FMN1
exm1145712	15	33954764	G	A	RYR3
exm1146153_ver4	15	34159941	G	T	AVEN
rs12591967	15	34574330	C	T	SLC12A6
exm1146681	15	34648935	T	G	NUTM1
exm1147313	15	38233381	A	C	TMCO5A
exm1147314	15	38233383	C	T	TMCO5A
exm1147331	15	38235552	A	G	TMCO5A
exm1147334	15	38239858	A	G	TMCO5A
exm1147607	15	39874396	G	T	THBS1
exm1148104	15	40308859	T	G	EIF2AK4
exm1148272	15	40498503	C	T	BUB1B
exm1149918	15	41105926	T	G	ZFYVE19
exm1149977	15	41137177	G	C	SPINT1
exm1150700	15	41634587	G	A	NUSAP1
exm1150817_ver3	15	41689232	T	C	NDUFAF1
exm1151205	15	41819367	C	T	RPAP1
exm1151527	15	41991315	A	T	MGA
exm1152764	15	42171483	A	G	SPTBN5
exm1152819	15	42178122	C	T	SPTBN5
exm1153416	15	42439444	T	C	PLA2G4F
exm1153816	15	42630673	G	A	GANC
exm1155607	15	43569097	T	C	TGM7
exm1156438	15	43818115	A	G	MAP1A
exm1157633	15	44907562	C	T	SPG11
exm1158182	15	45398369	T	C	DUOX2
exm1158840	15	45561551	T	C	SLC28A2

exm2252167	15	45981317	A	G	SQRDL
exm-rs4775785	15	49192326	C	T	SHC4
exm-rs8023445_ver2	15	49192791	C	T	SHC4
exm2272241_ver4	15	50232552	T	C	ATP8B4
exm1160717_ver3	15	50279662	C	T	ATP8B4
exm1161148	15	50878630	A	G	TRPM7
exm1162087	15	51975608	A	G	SCG3
exm1163278	15	54003091	A	G	WDR72
exm2264558	15	54018760	C	A	WDR72
exm1163422	15	54307925	T	C	UNC13C
exm2267836	15	54842584	A	G	UNC13C
exm1163709	15	55652719	G	A	CCPG1
exm1163860	15	55838585	T	G	PYGO1
exm1164163	15	56207811	G	T	NEDD4
exm1164197	15	56208463	C	A	NEDD4
exm-rs3743266	15	60781513	G	A	RORA
exm1166832	15	62207911	G	A	VPS13C
exm2252241	15	63623463	A	G	CA12
exm1168218	15	63937209	C	G	HERC1
exm1168269	15	63950887	A	G	HERC1
exm-rs4411464	15	63995423	C	T	HERC1
rs10152453	15	64098613	C	A	HERC1
exm1170209	15	65621441	C	A	IGDCC3
exm1170688	15	65863917	C	A	PTPLAD1
exm1170775	15	65917355	G	T	SLC24A1
exm1171367	15	66641732	C	G	TIPIN
exm-rs17293632	15	67442596	T	C	SMAD3
exm-rs7359257_ver3	15	67702907	A	C	IQCH-AS1
exm1172626	15	68628049	G	T	ITGA11
exm2267782	15	68640452	A	G	ITGA11
exm-rs7164335	15	68715032	A	G	ITGA11
exm2252270	15	69381742	A	G	LINC00277,MIR548H4
exm2273527	15	71403406	C	T	CT62
exm-rs12899618	15	71645120	A	G	THSD4
exm2267843	15	73554544	G	A	NEO1
exm1175523_ver2	15	74174064	A	G	TBC1D21
exm1176367	15	74622549	T	G	CCDC33
exm-rs7497036	15	74873679	G	A	ARID3B
exm-rs7181548	15	76409861	C	A	C15orf27

exm2264566	15	76700801	A	C	SCAPER
exm1179893	15	78337303	T	C	TBC1D2B
exm1181858	15	80263217	T	C	BCL2A1
rs1431716	15	83344932	A	G	RP11-752G15.3
exm1184129	15	84611367	C	G	ADAMTSL3
exm1184504	15	85198606	T	C	NMB
exm1186825	15	89401109	A	G	ACAN
exm1187119	15	89450587	G	T	MFGE8
exm1187791	15	90023558	A	G	RHCG
exm1187859	15	90126121	C	T	TICRR
exm1188200	15	90174824	C	T	KIF7
exm1188217	15	90176073	C	A	KIF7
exm1188832	15	90347814	A	G	ANPEP
exm1188875	15	90349558	C	T	ANPEP
exm1189336	15	90784165	A	G	GDPGP1
exm1189350	15	90784376	A	G	GDPGP1
exm1189565	15	90903311	A	G	ZNF774
exm2252379	15	91449702	T	A	MAN2A2
exm-rs3924426	15	92445873	C	T	SLCO3A1
exm1191463	15	93015721	G	A	C15orf32
exm-rs4984390	15	94939508	G	A	MCTP2
exm2252400	15	96856201	T	G	NR2F2-AS1
exm2260501	15	96873212	A	G	NR2F2
exm1192081	15	98513845	C	T	ARRDC4
exm1192393	15	99511873	A	G	PGPEP1L
exm1192937	15	100269749	A	G	LYSMD4
exm-rs4246302	15	100687967	G	A	ADAMTS17
exm2272289	15	101546195	C	T	LRRK1
exm1194456	15	101924546	T	C	PCSK6
exm1195887	16	336396	A	G	PDIA2
exm1195895	16	336438	T	C	PDIA2
exm2273533	16	337691	T	C	AXIN1
exm1199466	16	840569	T	C	CHTF18
exm1200019	16	1129010	A	C	SSTR5
exm1200627_ver4	16	1268376	A	G	CACNA1H
exm1200669	16	1269095	T	C	CACNA1H
exm1200771	16	1272275	T	C	TPSG1
exm1200796	16	1272684	G	C	TPSG1
exm1200805	16	1272750	A	G	TPSG1

exm1200831	16	1273490	T	C	TPSG1
exm1208672	16	2814950	T	C	SRRM2
exm1209328_ver2	16	2980828	A	G	FLYWCH1
exm1209828	16	3073240	T	G	HCFC1R1
exm1210665	16	3293888	T	C	MEFV
exm1211522	16	3606947	T	G	NLRC3
exm2272335	16	3747204	T	C	TRAP1
exm-rs2601828	16	4103871	A	G	ADCY9
exm1214279	16	4790204	G	C	C16orf71
exm1215019	16	4933939	C	G	PPL
exm1215265	16	4942099	T	C	PPL
rs1049205	16	4942099	A	G	PPL
exm1216129	16	8722629	C	G	METTL22
rs11074504	16	10034276	G	T	GRIN2A
rs12924396	16	10070285	G	C	GRIN2A
rs11641062	16	10082751	G	C	GRIN2A
rs11866385	16	10108658	G	C	GRIN2A
rs837692	16	10108788	A	G	GRIN2A
rs1969060	16	10117137	C	T	GRIN2A
rs1366076	16	10263950	A	T	GRIN2A
rs1650420	16	10268330	A	G	GRIN2A
exm1217028_ver3	16	10769958	C	T	TEKT5
exm1217246	16	10989219	G	C	CIITA
exm1217663_ver3	16	11362729	A	G	TNP2
exm-rs416603	16	11364079	T	A	TNP2
exm-rs7203193	16	11641180	G	A	LITAF
exm2267930	16	11887629	A	G	ZC3H7A
exm1220365	16	16173232	T	G	ABCC1
exm1220571	16	16251599	T	C	ABCC6
exm2272348	16	17549067	T	C	XYLT1
exm1221377	16	18866208	G	C	SMG1
exm1223535	16	20810266	C	T	ERI2
exm1224455	16	21185384	A	G	TMEM159
exm1224465	16	21190853	A	C	TMEM159
exm1224585	16	21222672	A	C	ZP2
exm1225055	16	21976762	A	G	UQCRC2
exm1225067	16	21982936	A	G	UQCRC2
exm1225124	16	22092067	C	A	C16orf52
exm1227414	16	24788645	A	T	TNRC6A

exm-rs151181	16	28490517	G	A	CLN3
exm1229715	16	28515228	C	A	IL27
exm2272358	16	28618708	T	C	SULT1A1
exm2273534	16	28995757	C	A	SPNS1
exm1231055	16	29791721	G	C	ZG16
exm1231094	16	29811027	A	T	KIF22
exm1231568_ver2	16	29891206	T	C	SEZ6L2
exm1232774	16	30380872	G	C	TBC1D10B
exm-rs10871454	16	31048079	T	C	STX4
exm1235282	16	31088625	G	A	ZNF646
rs889548	16	31137712	A	G	KAT8
exm1235743	16	31142271	G	A	KAT8
exm1237275	16	31926619	G	A	ZNF267
exm1238246	16	48122582	G	A	ABCC12
rs3760014	16	50319602	C	G	ADCY7
rs7191958	16	50332173	A	G	ADCY7
exm1240779	16	53639438	T	C	RPGRIP1L
exm1240800	16	53671754	T	C	RPGRIP1L
exm1241040	16	54318172	A	C	IRX3
rs3785152	16	55716550	T	C	SLC6A2
exm1243143	16	57068106	G	A	NLRC5
exm2272383	16	57689028	C	T	GPR56
exm1244622	16	57789288	C	T	KATNB1
exm1245121	16	58019396	G	A	TEPP
exm2272384_ver2	16	58529615	T	C	NDRG4
exm1245682	16	58540875	A	G	NDRG4
exm1247940	16	67219107	C	G	EXOC3L1
exm-rs3729639	16	67225501	T	C	E2F4
exm1248129	16	67234134	G	A	ELMO3
exm1248179	16	67235672	T	C	ELMO3
exm1248271	16	67241930	T	C	LRRC29
exm1249084	16	67434917	T	C	ZDHHC1
exm-rs2271293	16	67902070	A	G	NUTF2
exm-rs16942887	16	67928042	A	G	PSKH1
exm1250689	16	67976320	T	A	LCAT
exm1250890	16	67997920	C	T	SLC12A4
exm-rs255049	16	68013471	C	T	DPEP3
exm-rs255052	16	68024995	A	G	DPEP2
exm2267902	16	68068627	G	A	DUS2

exm-rs1728785	16	68591230	A	C	ZFP90
exm1252857	16	69385641	G	A	TMED6
exm1255175	16	70972595	T	C	HYDIN
exm1255571	16	71318577	C	T	CMTR2
exm1255738	16	71483497	G	C	ZNF23
exm-rs16973500	16	71965196	T	C	PKD1L3
exm1257086	16	72156842	G	T	PMFBP1
exm1257935	16	72993831	C	A	ZFHX3
exm-rs7193343	16	73029160	T	C	ZFHX3
exm-rs2106261	16	73051620	A	G	ZFHX3
exm1258929_ver5	16	75146529	G	C	LDHD
exm1259760	16	75646508	C	T	ADAT1
exm1260517	16	77401545	G	A	ADAMTS18
exm2267911	16	77940068	A	G	VAT1L
exm1260788	16	78458807	G	C	WWOX
exm1261391	16	81142257	C	T	PKD1L2
exm-rs4889240	16	81156522	C	T	PKD1L2
exm1261455_ver2	16	81157385	T	G	PKD1L2
exm1261567	16	81181097	T	G	PKD1L2
exm1261661_ver2	16	81193321	T	C	PKD1L2
exm1262022	16	81253759	A	G	PKD1L2
exm1262760	16	82185112	C	T	MPHOSPH6
exm1263816_ver2	16	84199444	G	C	DNAAF1
exm1264651	16	84476200	T	A	ATP2C2
exm1264699	16	84485677	A	T	ATP2C2
exm1264949	16	84522897	C	G	TLDC1
exm-rs2696835	16	86365571	C	G	LINC00917
exm1266315	16	86566039	T	C	MTHFSD
exm1266316	16	86566042	A	G	MTHFSD
exm2252794	16	86575737	T	C	MTHFSD
exm1266461	16	86613143	A	G	FOXL1
exm1266761	16	87493737	C	T	ZCCHC14
exm1268532	16	88779739	G	A	CTU2
exm1268756	16	88787610	C	T	PIEZO1
exm1269333	16	88909159	T	G	GALNS
exm1270038	16	89294044	G	A	ZNF778
exm1270431	16	89350038	G	A	ANKRD11
exm2264606	16	89587871	C	A	SPG7
exm1270713	16	89613123	G	A	SPG7

exm-rs12931267	16	89818732	G	C	FANCA
exm1271818	16	89833576	C	G	FANCA
exm1272378	16	89986117	T	C	MC1R
exm1273007	17	63683	A	G	RPH3AL
exm1273677	17	685640	T	G	RNMTL1
exm1274010	17	1183354	T	C	TUSC5
exm1274648	17	1540106	A	G	SCARF1
exm1275626	17	1703982	T	C	SMYD4
exm1276042	17	1961863	T	C	HIC1
exm-rs1231206	17	2125605	T	C	SMG6
exm-rs216172	17	2126504	G	C	SMG6
exm1276175	17	2202323	C	T	SMG6
exm-rs4790333	17	2262703	T	C	SGSM2
exm1276501	17	2266799	A	G	SGSM2
exm2252901	17	2266812	C	T	SGSM2
exm1276525	17	2268311	A	G	SGSM2
exm-rs12603284	17	2771512	T	C	RAP1GAP2
exm1280781	17	4463713	G	A	GGT6
exm1280869	17	4535035	A	G	ALOX15
exm1281471_ver3	17	4689313	C	G	VMO1
exm1282028	17	4806052	A	C	CHRNE
exm1283844	17	5347788	A	G	DHX33
exm1283875	17	5354204	C	G	DHX33
exm1287615	17	7366348	C	A	ZBTB4
exm1288071	17	7462555	A	G	TNFSF13
exm1288274	17	7484101	A	C	CD68
exm1288331	17	7490810	A	G	MPDU1
exm1288441	17	7529902	A	G	SAT2
exm1290319	17	7846836	T	A	CNTROB
exm1290628	17	7950265	G	A	ALOX15B
exm1291196_ver2	17	8053916	A	G	PER1
exm1291977	17	8216468	T	C	ARHGEF15
exm1293419	17	9792768	A	G	GLP2R
exm1294988	17	10534960	T	C	MYH3
exm1295110	17	10542709	A	G	MYH3
exm2252857	17	13949828	A	G	COX10-AS1
exm1304458	17	21319792	A	G	KCNJ12,KCNJ18
exm1304524	17	25628820	T	C	WSB1
exm1304672	17	25931718	C	G	KSR1

exm1304879	17	26096597	A	G	NOS2
exm1305032	17	26519204	T	C	NLK
exm2252913	17	26519204	T	C	NLK
exm2225997	17	27224799	C	T	DHRS13
exm1307334	17	27284443	G	A	SEZ6
exm1308622	17	28405431	A	G	EFCAB5
rs8081028	17	28523314	A	C	SLC6A4
rs2020935	17	28561455	T	A	SLC6A4
rs2020933	17	28561755	A	T	SLC6A4
exm1308979	17	28749880	G	A	CPD
exm1309133	17	29111368	G	A	CRLF3
exm1309662	17	29623288	T	C	OMG
exm1310094	17	30222002	C	T	UTP6
exm1311123	17	32957114	A	G	TMEM132E
exm1311298	17	33289046	C	G	ZNF830
exm1312511	17	33806546	A	G	SLFN12L
exm1312755	17	34072031	A	G	GAS2L2
exm1312943_ver4	17	34093663	A	G	MMP28
exm1312945	17	34093681	A	G	MMP28
exm1313248	17	34195739	C	T	C17orf66
exm1313353	17	34304605	A	C	CCL16
exm1314986	17	36478450	T	G	MRPL45
exm2252969	17	37074932	T	C	LASP1
exm1316757_ver2	17	37686920	T	C	CDK12
rs907094	17	37790371	C	T	PPP1R1B
exm1316877	17	37815304	C	G	STARD3
exm-rs907092	17	37922259	A	G	IKZF3
exm-rs9303277	17	37976469	T	C	IKZF3
exm1317501	17	38028634	T	G	ZPBP2
exm1317549	17	38062196	A	G	GSDMB
exm1317550	17	38062217	T	C	GSDMB
exm1317567	17	38064469	C	T	GSDMB
exm-rs2290400_ver2	17	38066240	G	A	GSDMB
exm-rs7216389	17	38069949	C	T	GSDMB
rs2071570	17	38257090	T	G	NR1D1
exm1319461	17	38956007	G	A	KRT28
exm1319935	17	39135207	G	A	KRT40
exm1321384	17	39593722	T	C	KRT38
exm1321838	17	39659194	T	C	KRT13

exm1322775	17	39925713	T	C	JUP
exm1322928	17	39974642	G	A	FKBP10
exm1323322	17	40086986	T	C	TTC25
exm-rs744166	17	40514201	C	T	STAT3
exm2268006	17	40529835	A	G	STAT3
exm1326256	17	41131645	A	C	PTGES3L,PTGES3L-AARSD1
exm1326638_ver3	17	41245471	T	C	BRCA1
exm1327511	17	42030531	G	C	PYY
exm2253021	17	42031331	C	T	PYY
rs9900679	17	43868158	C	G	CRHR1
exm1332974	17	46022065	A	G	PNPO
exm-rs2084881	17	46357120	A	G	SKAP1
exm-rs9303542	17	46411500	G	A	SKAP1
exm1333519	17	46629593	G	T	HOXB3
exm1333657	17	46688256	T	C	HOXB7
exm1333942	17	46929908	C	T	CALCOCO2
exm-rs2072153	17	47390014	G	C	ZNF652
exm-rs16948048	17	47440466	G	A	ZNF652
exm1335893	17	48452714	G	A	EME1
exm1336233	17	48561074	C	G	RSAD1
exm1337278	17	48761053	A	G	ABCC3
exm1337433	17	48913390	A	G	WFIKKN2
exm1338023	17	51901503	C	G	KIF2B
exm1338026	17	51901553	A	G	KIF2B
exm1338031_ver3	17	51901643	C	T	KIF2B
exm1338036	17	51901703	C	G	KIF2B
exm1338461	17	54534634	G	A	ANKFN1
exm1338859	17	55182878	T	C	AKAP1
exm-rs792376	17	55438556	T	C	MSI2
exm1340102	17	56395757	T	C	BZRAP1
exm1341762	17	57963537	G	A	TUBD1
exm1342412_ver2	17	58824617	A	G	BCAS3
exm1342832	17	59763347	A	G	BRIP1
rs4291	17	61554194	T	A	ACE
rs4293	17	61555666	A	G	ACE
rs4295	17	61556298	G	C	ACE
rs4311	17	61560763	T	C	ACE
exm1344131	17	61562373	G	A	ACE
rs4329	17	61563458	G	A	ACE

rs4333	17	61564522	C	T	ACE
exm-rs4343	17	61566031	A	G	ACE
rs4362	17	61573761	C	T	ACE
rs4363	17	61574492	A	G	ACE
exm1346358_ver3	17	62854937	G	C	LRRC37A3
exm-rs9912468	17	64318357	G	C	PRKCA
exm1348190	17	66246416	A	G	AMZ2
exm1348524	17	66533655	A	G	FAM20A
exm2260701	17	70712513	A	G	SLC39A11
exm1350694	17	71346513	T	C	SDK2
exm1350779	17	71380087	C	G	SDK2
exm1352013_ver3	17	72588778	T	C	C17orf77
exm1352035	17	72610089	T	C	CD300E
exm1353904	17	73263012	T	C	MIF4GD
exm1356757	17	74003846	A	G	EVPL
exm1357044	17	74014634	T	G	EVPL
exm1357153	17	74042169	A	G	SRP68
exm1360306	17	76219591	G	A	BIRC5
exm1361017	17	76491127	T	C	DNAH17
exm1361078	17	76497920	C	A	DNAH17
exm2276547	17	76503397	A	G	DNAH17
exm1361118	17	76503560	A	C	DNAH17
exm1361186	17	76522785	T	C	DNAH17
exm2253267	17	77318992	A	G	RBFOX3
exm1362909	17	77984254	T	C	TBC1D16
exm1363660	17	78178893	T	C	CARD14
exm2253280	17	78178916	T	C	CARD14
exm1364056	17	78262161	C	T	RNF213
exm1364064	17	78263486	C	T	RNF213
exm1364433	17	78321157	C	T	RNF213
exm1367363	17	79634774	T	A	CCDC137
exm1367384	17	79638805	T	C	CCDC137
exm-rs1046896	17	80685533	T	C	FN3KRP
exm1370860	17	80789468	A	G	ZNF750
exm1370866	17	80789628	C	T	ZNF750
exm1371776	18	907710	G	A	ADCYAP1
rs2856966	18	907710	G	A	ADCYAP1
exm1372334	18	2929103	A	G	LPIN2
exm1372588	18	3168816	A	G	MYOM1

exm1372759	18	3457606	T	C	TGIF1
exm1372990	18	5396324	T	G	EPB41L3
exm2268071	18	5441359	A	G	EPB41L3
exm2264663	18	6107360	A	C	L3MBTL4
exm-rs2302768	18	12718593	G	A	PSMG2
exm2260731	18	13261896	A	G	LDLRAD4
exm1379822_ver3	18	23866185	C	G	TAF4B
exm1380150	18	25593694	T	C	CDH2
exm1381286	18	29172865	A	G	TTR
exm1381723	18	29797921	T	C	MEP1B
exm-rs507163	18	33907628	C	T	FHOD3
exm1383486	18	34324091	A	G	FHOD3
exm2253426	18	40655271	A	G	RIT2
exm-rs4130047	18	40678235	C	T	RIT2
exm1384191_ver2	18	43206985	A	G	SLC14A2
exm-rs4890568	18	43231622	G	A	SLC14A2
exm1384338	18	43307246	T	C	SLC14A1
exm-rs17674580	18	43309911	T	C	SLC14A1
exm1384354	18	43310415	A	G	SLC14A1
exm1384398_ver3	18	43319274	A	G	SLC14A1
exm1385257_ver2	18	44143153	T	C	LOXHD1
exm-rs11661691	18	46770186	G	T	DYM
exm-rs9967417	18	46959500	G	C	DYM
exm1386298	18	47093864	T	C	LIPG
exm1386761_ver5	18	47566678	G	C	MYO5B
exm-rs7506909	18	50620087	A	G	DCC
exm2268106	18	50768168	A	G	DCC
exm1387798_ver4	18	51820805	G	A	POLI
exm1388325	18	55021725	C	A	ST8SIA3
exm-rs10503019	18	55454377	A	G	ATP8B1
exm2272739	18	55768995	C	T	NEDD4L
exm1388740	18	55816791	A	G	NEDD4L
exm1389580	18	57026436	T	C	LMAN1
exm1389981	18	59780393	A	T	PIGN
exm1391518	18	64211251	T	C	CDH19
exm2268158	18	67695505	G	A	RTTN
exm1392042	18	67718688	A	G	RTTN
exm1392471	18	71740743	G	A	FBXO15
exm1392960	18	72344509	A	G	ZNF407

exm1393394	18	72999359	C	T	TSHZ1
rs2717162	18	74968327	C	T	GALR1
exm2253750	19	288062	A	G	PPAP2C
exm1395878	19	467664	A	G	ODF3L2
exm1397117	19	757241	A	G	MISP
exm1397560	19	830820	A	G	AZU1
exm1398804	19	1043748	G	A	ABCA7
exm1398819	19	1044712	G	A	ABCA7
exm1398887	19	1048898	C	G	ABCA7
exm1399512	19	1105723	G	A	GPX4
exm1401036	19	1487195	A	G	PCSK4
exm1401678	19	1784944	G	A	ATP8B3
exm1401976	19	1819125	C	T	REXO1
exm1401998	19	1821637	A	G	REXO1
exm1402351	19	1924189	T	C	SCAMP4
exm1403865	19	2344034	A	C	SPPL2B
exm1403922	19	2389872	G	C	TMPRSS9
exm1403936_ver2	19	2396611	T	C	TMPRSS9
exm1404129	19	2422177	A	G	TMPRSS9
exm2272753	19	2565877	T	C	GNG7
exm1405021	19	2917287	A	C	ZNF57
exm-rs7257762	19	3281586	G	A	CELF5
exm1406311	19	3548168	C	T	MFSD12
exm2268173	19	3739673	A	G	TJP3
exm1406966	19	3750615	T	C	TJP3
exm1407028	19	3752874	A	G	APBA3
exm1407418	19	3831525	A	C	ZFR2
exm1407433_ver2	19	3831765	A	G	ZFR2
exm1408655	19	4294623	A	C	TMIGD2
exm1408915	19	4359191	T	C	MPND
exm2268214	19	4539086	G	A	LRG1
exm1410803	19	4910889	T	G	UHRF1
exm1411567	19	5455735	A	G	ZNRF4
exm1411569	19	5455800	A	G	ZNRF4
exm1412204_ver3	19	5711930	T	C	LONP1
exm1415005	19	6826630	A	G	VAV1
exm1415059_ver2	19	6850767	T	C	VAV1
exm1416871	19	7712277	A	G	STXBP2
exm1417660	19	7986638	G	C	SNAPC2

exm1417873	19	8121096	A	G	CCL25
exm1417882	19	8121360	G	A	CCL25
exm1417980	19	8145928	T	C	FBN3
exm1418115	19	8161777	T	C	FBN3
exm1418413	19	8197958	T	C	FBN3
exm1419020	19	8486884	A	G	MARCH2
exm1419224	19	8556035	A	G	PRAM1
exm-rs7249094	19	8672000	A	G	ADAMTS10
exm1420074	19	8987218	T	G	MUC16
exm1420192	19	9001833	A	G	MUC16
exm1422391_ver4	19	9213651	T	C	OR7G2
exm1422435	19	9225940	A	G	OR7G1
exm1422436	19	9225973	A	G	OR7G1
exm1422477	19	9236917	A	G	OR7G3
exm1423418	19	9868278	C	T	ZNF846
exm1423426	19	9868404	T	C	ZNF846
exm1423817	19	10085062	T	C	COL5A3
exm1424152	19	10132000	A	G	RDH8
exm1425126	19	10395208	T	C	ICAM1
exm1425579	19	10463118	C	G	TYK2
exm1425969	19	10577843	A	C	PDE4A
exm1426190	19	10664632	G	A	KRI1
exm1427684	19	11303554	G	A	KANK2
exm1428210	19	11408905	G	T	TSPAN16
exm-rs17638629	19	12225372	G	T	ZNF788
exm1430448	19	12460783	C	G	ZNF442
exm1431178	19	12780204	C	T	WDR83OS
exm1433071	19	13411451	G	T	CACNA1A
exm1433638	19	14003969	C	G	C19orf57
exm1434386	19	14165204	C	G	PALM3
exm1434873	19	14512256	T	C	CD97
exm1436897	19	15289850	A	T	NOTCH3
exm1437882	19	15586672	T	A	PGLYRP2
exm1437965	19	15648456	T	A	CYP4F22
exm1438510	19	15839496	G	A	OR10H2
exm1440202	19	16899865	G	A	NWD1
exm1440914	19	17108127	T	C	CPAMD8
exm1441444	19	17337882	G	C	OCEL1
exm1441731	19	17392894	A	G	ANKLE1

exm1441736	19	17393015	C	T	ANKLE1
exm1441757_ver2	19	17394124	G	T	ANKLE1
exm1441778_ver4	19	17394504	A	C	ANKLE1
exm1441796	19	17395003	A	G	ANKLE1
exm1441910	19	17412218	T	C	ABHD8
exm1442152	19	17450016	T	C	GTPBP3
rs2287855	19	17885880	A	G	FCHO1
exm1443974	19	18112361	T	C	ARRDC2
exm1444340_ver3	19	18255359	A	G	MAST3
exm2273571_ver4	19	18288898	C	T	IFI30
exm1444680	19	18329784	T	C	PDE4C
exm1444835	19	18375531	A	G	KIAA1683
exm1448237	19	19757073	C	A	ATP13A1
exm-rs10411195	19	20036176	C	T	ZNF93
exm1448846	19	20228737	G	T	ZNF90
exm1448860	19	20229486	A	G	ZNF90
exm1449013_ver3	19	20728777	C	A	ZNF737
exm1449170_ver3	19	21131664	T	G	ZNF85
exm1449181	19	21131850	G	C	ZNF85
exm1452477	19	33110204	T	C	ANKRD27
exm-rs3848596	19	33635761	T	C	WDR88
exm2264680	19	33652247	A	C	WDR88
exm1453731	19	33878837	A	G	PEPD
exm1454713	19	35422808	T	C	ZNF30
exm1454723	19	35434238	A	G	ZNF30
exm1455425	19	35719106	A	G	FAM187B
exm1455449	19	35719577	A	G	FAM187B
exm1456275	19	36003962	C	T	DMKN
exm1458081	19	36339295	G	A	NPHS1
exm1458153	19	36348078	C	A	KIRREL2
exm1458175	19	36349752	A	G	KIRREL2
exm1459024	19	36595436	A	C	WDR62
exm2272786	19	36904463	T	C	ZFP82
exm2253803	19	37835658	T	C	HKR1
exm1460406	19	37854040	A	G	HKR1
exm1461033	19	38160565	T	C	ZNF781
exm-rs16975963	19	38325536	G	C	AC016582.2
exm1462950	19	38983180	T	G	RYR1
exm1464063	19	39307103	T	C	ECH1

exm1467266	19	40519935	T	C	ZNF546
exm1470180_ver2	19	41512841	T	G	CYP2B6
exm1471675	19	42225060	A	C	CEACAM5
exm1475553	19	43858044	T	A	CD177
exm1476325	19	44153100	G	A	PLAUR
exm1476933	19	44470331	G	A	ZNF221
exm1478756	19	45179567	T	C	CEACAM19
exm1479141	19	45322962	T	A	BCAM
exm-rs4420638	19	45422946	G	A	APOC1
exm1480027	19	45682876	G	C	BLOC1S3
exm-rs4884	19	45810035	T	C	CKM
exm-rs8111071	19	46307406	G	A	RSPH6A
exm1483044	19	46915114	C	A	CCDC8
exm1483240_ver2	19	46998236	T	C	PNMAL2
exm1484639	19	47856336	T	C	DHX34
exm1486031	19	48543862	G	A	CABP5
exm1486674	19	48821757	A	G	CCDC114
exm1486793	19	48869178	T	C	SYNGR4
exm1488339	19	49318380	A	G	HSD17B14
exm1488583	19	49377319	G	A	PPP1R15A
exm1488921	19	49447713	A	G	DHDH
exm1489460	19	49573438	G	A	KCNA7
exm1489492_ver3	19	49574125	C	G	KCNA7
exm1489602_ver4	19	49621964	T	C	C19orf73
exm-rs11667393	19	49662027	G	A	TRPM4
exm1491231	19	50093248	T	A	PRRG2
exm1493682	19	50758572	C	G	MYH14
exm1495095	19	51170706	A	G	SHANK1
exm1495520	19	51323232	C	T	KLK1
exm1495845	19	51462454	A	G	KLK6
exm2273575	19	51517731	A	G	KLK10
exm1496089	19	51520487	A	C	KLK10
exm1496168	19	51530741	G	C	KLK11
exm1499276	19	52887131	A	G	ZNF880
exm1499605	19	53014346	G	A	ZNF578
exm1499653	19	53015384	G	A	ZNF578
exm1499790	19	53085659	C	T	ZNF701
exm1501082	19	53668697	C	T	ZNF665
exm1501105	19	53669273	T	C	ZNF665

exm1501182	19	53741219	T	C	ZNF677
exm1501354	19	53792954	A	G	BIRC8
exm1501355	19	53792955	T	C	BIRC8
exm1505733	19	55174498	C	T	LILRB4
exm1506656	19	55396913	A	G	FCAR
exm1508578	19	55824332	T	G	TMEM150B
exm1508758	19	55870260	A	G	FAM71E2
exm1510549	19	56419263	C	T	NLRP13
exm1511104	19	56569629	G	C	NLRP5
exm1511321	19	56703248	A	G	ZSCAN5B
exm1513768	19	58017753	A	C	ZNF773
exm1513770	19	58017758	C	G	ZNF773
exm2254050	19	58058739	C	T	ZNF550
exm1514142	19	58117223	T	A	ZNF530
exm1514214	19	58131576	C	T	ZNF134
exm1514251	19	58132106	C	A	ZNF134
exm1514343	19	58152795	T	C	ZNF211
exm1514664	19	58233008	A	G	ZNF671
exm1515721	19	58596449	T	C	ZSCAN18
exm2254185	20	210061	A	G	DEFB129
exm1519013	20	1459246	T	C	SIRPB2
exm2254161	20	1538249	T	C	SIRPD
rs6035222	20	1963413	A	G	PDYN
rs1997794	20	1974858	C	T	PDYN
exm1519538	20	2290333	C	A	TGM3
exm1519809_ver3	20	2397883	T	C	TGM6
exm1522296	20	3675333	G	A	SIGLEC1
exm1522975_ver3	20	3843027	A	C	MAVS
exm1523019	20	3846397	T	C	MAVS
exm1523270	20	4202636	G	C	ADRA1D
exm1523417	20	4705718	C	T	PRND
exm2272873	20	5762821	T	C	C20orf196
exm-rs4815868	20	5840539	G	A	C20orf196
exm1523947	20	5903141	C	G	CHGB
exm1524552	20	7864284	C	T	HAO1
exm1525185	20	10030188	A	T	ANKEF1
exm1525201	20	10030452	A	G	ANKEF1
exm-rs17190927	20	13123733	G	T	SPTLC3
exm1526136	20	13912309	A	G	SEL1L2

exm2268275	20	14031392	A	G	MACROD2
exm1526162	20	14066276	T	C	MACROD2
exm-rs6110577	20	15335754	C	T	MACROD2
exm1526549	20	16729138	C	T	OTOR
exm1528175	20	20032998	C	T	CRNKL1
exm1529890	20	23860178	G	A	CST5
exm1531308	20	29896333	A	T	DEFB116
exm1533375	20	31590686	A	C	SUN5
exm1533395	20	31596472	T	A	BPIFB2
exm1533488	20	31619500	T	C	BPIFB6
exm1533654	20	31652596	T	C	BPIFB3
exm1533708	20	31660543	T	C	BPIFB3
exm1533775	20	31671619	T	G	BPIFB4
exm1533958_ver2	20	31767470	C	G	BPIFA2
exm1536059	20	33574773	G	A	MYH7B
exm-rs619865	20	33867697	A	G	EIF6
exm-rs6060369	20	33907161	C	T	UQCC1
exm-rs6088792	20	33909784	T	C	UQCC1
exm-rs6060373	20	33914208	G	A	UQCC1
exm1536954	20	33971914	C	T	UQCC1
exm-rs6088813	20	33975181	C	A	UQCC1
exm1537001	20	34022387	C	A	GDF5
exm-rs143384	20	34025756	T	C	GDF5
exm2268339	20	34025983	G	A	GDF5
exm-rs2236164	20	34097353	G	A	CEP250
exm1537365	20	34116282	C	T	C20orf173
exm1537606	20	34219496	C	T	CPNE1
exm2264712	20	34373979	A	C	PHF20
exm2268291	20	34432670	G	A	PHF20
exm1538095	20	34596371	T	C	CNBD2
exm2260932	20	35990792	C	T	SRC
exm1540026	20	36841914	A	G	KIAA1755
exm1540133	20	36869005	A	G	KIAA1755
exm1540445	20	37001761	C	T	LBP
exm1540977	20	37580670	G	C	FAM83D
exm1541023	20	37621052	C	T	DHX35
exm1541124_ver3	20	37667182	T	C	DHX35
exm2268297	20	40783392	G	A	PTPRT
exm1542398	20	41420095	C	T	PTPRT

exm-rs6031252	20	42573822	A	C	TOX2
exm-rs6017291	20	42854134	G	A	OSER1-AS1
exm1543200	20	42965841	G	T	R3HDML
exm1543930_ver3	20	43629135	A	G	STK4
exm1544384	20	43933021	T	G	MATN4
exm1544474	20	43942676	C	T	RBPJL
exm2254295	20	44664493	T	G	SLC12A5
exm2272935	20	45269556	T	C	SLC13A3
exm-rs4810685	20	46400713	T	C	SULF2
exm1548904	20	47989624	A	G	KCNB1
exm2268305	20	48011008	A	G	KCNB1
exm2254318	20	49196284	T	C	PTPN1
exm1549641	20	49214156	A	G	FAM65C
exm-rs6013382	20	50702633	T	C	ZFP64
exm1552320	20	56087800	C	T	CTCFL
exm1553857	20	57829301	C	T	ZNF831
exm1554183	20	58476811	T	C	SYCP2
exm1555877	20	60899206	C	T	LAMA5
exm-rs4925386	20	60921044	T	C	LAMA5
exm1556537	20	60989365	T	C	RBBP8NL
exm1557313	20	61459315	T	C	COL9A3
rs3818204	20	61991141	A	G	CHRNA4
rs12624510	20	61992894	A	G	CHRNA4
rs6122429	20	61993206	T	C	CHRNA4
exm1558832_ver3	20	62038277	T	G	KCNQ2
exm1562523	21	15596772	T	G	RBM11
exm1563101	21	19635116	G	C	CHODL
exm-rs2830051	21	27465355	C	T	APP
exm1563904	21	28302355	A	G	ADAMTS5
exm-rs239713	21	28696476	T	C	MIR5009
exm1564189	21	30339234	C	T	LTN1
exm1564210	21	30341891	A	G	LTN1
exm1564465	21	30439335	T	C	CCT8
exm1564625	21	30925928	G	A	GRIK1
rs363504	21	30925928	C	T	GRIK1
exm2273006	21	31110503	T	C	GRIK1
rs2178865	21	31128196	G	A	GRIK1-AS1
rs466013	21	31198707	T	C	GRIK1
exm1565212	21	31874240	C	T	KRTAP19-5

exm1566100	21	33684022	G	C	MRAP
exm1566529	21	33951068	C	T	TCP10L
exm1567138	21	34640788	G	A	IL10RB
exm1567989_ver4	21	34960634	C	G	DONSON
exm1568042	21	34997018	T	C	CRYZL1
exm1569577	21	38117308	A	C	SIM2
exm2268391	21	38268859	A	G	HLCS
exm-rs12483205	21	38740824	G	A	DYRK1A
exm2268371	21	39788804	G	A	ERG
exm1571773	21	42717662	T	C	FAM3B
exm2254636	21	43161877	T	C	RIPK4
exm2254637	21	43162150	A	G	RIPK4
exm1572424	21	43221483	A	G	PRDM15
exm-rs2187239	21	43420555	C	A	ZBTB21
exm1573712	21	43808627	T	C	TMPRSS3
exm1573724	21	43809092	T	C	TMPRSS3
exm2273011	21	44088631	C	T	PDE9A
exm1574550	21	44324329	A	G	NDUFV3
exm1574554	21	44324365	G	A	NDUFV3
exm2254658	21	45209559	T	C	RRP1
exm1575593	21	45391272	T	C	AGPAT3
exm1576212	21	45656774	T	C	ICOSLG
exm1578297	21	46057666	T	A	KRTAP10-10
exm2272989	21	46334472	T	C	ITGB2
exm1579158	21	46703410	T	C	POFUT2
exm1579364	21	46888290	T	C	COL18A1
exm1579947	21	47404302	A	G	COL6A1
exm1580209	21	47423389	A	G	COL6A1
exm1582151	21	47852085	A	G	PCNT
exm1582490	21	48063476	C	G	PRMT2
exm1582750	22	17264565	G	T	XKR3
exm1582909	22	17469049	A	C	GAB4
exm-rs5748919	22	17644131	T	C	CECR5-AS1
exm1583277	22	17669306	C	T	CECR1
exm1583918	22	18310439	A	G	MICAL3
exm1584098_ver3	22	18389548	C	G	MICAL3
exm1584182	22	18609128	T	C	TUBA8
exm1584516	22	19026613	G	A	DGCR2
exm1584666	22	19119751	T	C	TSSK2

exm1584715	22	19124865	T	C	DGCR14
exm1584927	22	19198017	A	T	CLTCL1
rs4646312	22	19948337	C	T	COMT
rs3810595	22	19949644	G	C	COMT
rs6269	22	19949952	G	A	COMT
rs2239393	22	19950428	G	A	COMT
rs740601	22	19950763	C	A	COMT
rs174696	22	19953176	C	T	COMT
rs9332377	22	19955692	T	C	COMT
rs12485043	22	19977840	T	C	ARVCF
exm1586435	22	20103263	T	G	TRMT2A
exm2273588	22	21306743	A	G	CRKL
exm1587989	22	21333605	C	G	AIFM3
exm1588854	22	21988833	T	C	CCDC116
exm1588883	22	21989325	T	C	CCDC116
rs12172554	22	22195075	T	C	MAPK1
exm2268404	22	23412017	A	G	RTDR1
exm-rs5751614	22	23593051	A	G	BCR
exm1592905_ver3	22	23974200	G	A	C22orf43
exm1593372	22	24179922	C	G	DERL3
exm1594144	22	24582041	G	A	SUSD2
exm1594287_ver3	22	24622648	C	T	GGT5
exm1594978	22	25145471	A	G	PIWIL3
rs6004726	22	26033507	T	C	ADRBK2
exm1595697	22	26157068	G	C	MYO18B
exm1595885	22	26231312	C	G	MYO18B
exm1596077	22	26422980	G	A	MYO18B
exm1596088	22	26423124	C	G	MYO18B
exm-rs4823006	22	29451671	G	A	ZNRF3
exm-rs36600_ver3	22	30337586	T	C	MTMR3
exm-rs5763662	22	30378703	T	C	MTMR3
exm1599163	22	30688659	T	C	TBC1D10A
exm1600144	22	31013419	T	C	TCN2
exm1601486	22	31971351	T	C	SFI1
exm1601962	22	32200849	T	C	DEPDC5
exm-rs132628	22	36541825	T	C	APOL3
exm1603962	22	36556823	G	T	APOL3
exm1604159	22	36661330	G	A	APOL1
exm1604172	22	36661566	G	A	APOL1

exm1604176	22	36661646	G	A	APOL1
exm1605014	22	37326443	C	G	CSF2RB
exm1605219	22	37407109	C	G	TST
exm1605685	22	37578652	A	G	C1QTNF6
rs738499	22	41777100	G	T	TEF
exm1612930	22	42095658	G	T	MEI1
exm1615520	22	43950949	A	G	EFCAB6
exm1615698	22	44131803	T	C	EFCAB6
exm1615803	22	44282276	G	A	PNPLA5
exm1615889	22	44322970	T	G	PNPLA3
exm1615904	22	44324727	G	C	PNPLA3
exm-rs2281135	22	44332570	T	C	PNPLA3
exm1615990	22	44368122	G	A	SAMM50
exm2273041	22	44390568	T	C	SAMM50
exm1616070	22	44395451	C	T	PARVB
exm1616086	22	44489868	T	C	PARVB
exm1616279	22	44681317	A	G	KIAA1644
exm1616670	22	45258373	C	G	ARHGAP8,PRR5-ARHGAP8
exm1617149	22	45749966	C	G	SMC1B
exm1617180	22	45767391	T	C	SMC1B
exm1618477	22	46760086	T	C	CELSR1
exm1618494	22	46760481	G	C	CELSR1
exm1623138	22	50943232	A	G	LMF2
exm1624169	22	51153371	A	G	SHANK3
