# Supplementary material for: Multivariate Imaging Genetics Study of MRI Gray Matter Volume and SNPs Reveals Biological Pathways Correlated with Brain Structural Differences in Attention Deficit Hyperactivity Disorder
Source: Front Psychiatry. 2016 Jul 25;7:128. doi: 10.3389/fpsyt.2016.00128 (PMC4959119; doi:10.3389/fpsyt.2016.00128)
Supplement: Supplementary file 3 [file Table_3.PDF]

Supplementary Table 3.

Significant genes in component G2 (after gene-size correction)
AIF1, PRRC2A, BAG6, GPANK1, LY6G5B, ACE, C6orf47, CELF5, ZFHX3, LDLRAD4, NTRK1, SH2D2A, NTRK1, ARRDC4, VARS, MSH5-SAPCD1, DYM, RHD, ADRB2, UGGT2, TG, NPAS2, SPON1, TBC1D8, IKZF3, ZPBP2, SLC44A4, CARD14, DZIP1, HSPA1L, CLDN10, SCARB1, RAET1E, GRIN2B, OPN4, CFB, ADCYAP1, C2, STK19, SKIV2L, RIN3, SLC35F3, STON1, GAP43, PLAC8L1, FNDC1, TNFAIP3, GRHL3, LARS, PODN, KCTD16, SLC9A9, WDR88, C6orf10, RNF144A, CAMTA1, TFRC, FAM131C, CTD-2127H9.1, DAO, TREML4, RBFOX3, GRM3, ATP8B3, PKD1L2, ZAN, VPS37C, GABRB1, CEP104, VSNL1, ACER3, TTC8, TNP2, TMPRSS9, C11orf80, AGAP1, SPAG17, COL6A1, C7orf50, ASS1, COL17A1, GRIA1, MICA, CRNKL1, ADAMTS3, PTPRD, CYP2E1, SIPA1L1, SMPD2, GIMAP7, NTSR2, CACNA1C, DOCK2, ZBTB24, CCDC110, CHRNA7, HIVEP2, GNG4, PASK, NCKAP5, PPP1R7, TAS2R41, DOCK4, KIAA0226L, TIMELESS, APOL1, ZSCAN5B, ZNF831, PDGFD, RPH3A, NAALADL2, ZNF652, CREB3L2, RAD54B, THSD4, FRMD3, CPQ, ME3, ZBTB21, PABPC4L, CTSF, BDNF-AS, NPSR1, TEK, ADCY9, PTGES2, CACNB4, ITGB6, CASC1, KIAA1462, BICD1, PTGES3, RASGRF2, KCNA7, CRLF3, GDF5, CD200R1, SHROOM3, FAM198A, IGDCC3, OR5AC2, TFAP2A, NDRG4, WDR46, LYSMD4, CDH18, FAM3B, OR52B6, HCRTR2, KCNIP1, OR9G4, AGBL4, FAM120AOS, C14orf169, SLC5A8, ESPNL, PTTG2, SOX5, ACACB
