# Supplementary material for: Multivariate Imaging Genetics Study of MRI Gray Matter Volume and SNPs Reveals Biological Pathways Correlated with Brain Structural Differences in Attention Deficit Hyperactivity Disorder
Source: Front Psychiatry. 2016 Jul 25;7:128. doi: 10.3389/fpsyt.2016.00128 (PMC4959119; doi:10.3389/fpsyt.2016.00128)
Supplement: Supplementary file 4 [file Table_4.PDF]

Supplementary Table 4. Significant regions of brain phenotype component S1

Region	Hemisphere	Peak coordinate	Cluster Size	Z-value
Anterior and mid-cingulate	L	-7.5, 45, 9	5461	5.2
Insula	L	-34.5, 21, 4.5	532	2.9
Insula	R	34.5, 19.5, 6	601	2.8
Cerebellum	L	-18, -82.5, -45	110	2.4
Thalamus	R	6, -19.5, 7.5	137	2.4
Posterior cingulate	L	-16.5, -57, 4.5	161	2.3
Posterior cingulate	R	16.5, -57, 4.5	274	2.1
Thalamus	L	-4.5, -21, 6	61	2
Cerebellum	R	18, -78, -34.5	54	1.9
Inferior temporal/ fusiform gyri	L	28.7, 0, -46.5	549	-2.6
Middle temporal/ fusiform/ parahippocampal gyri	L	-51, -45, -18	677	-2.3
Cerebellum	L	-4.5, -55.5, -24	185	-2.3
Brainstem	L	-6, -25.5, -6	97	-2.1
Inferior temporal/ fusiform gyri	R	54, -9, -37.5	243	-2.1
Inferior temporal gyrus	R	52.5, -36, -24	69	-2.1
Superior temporal gyrus	R	60, -55.5, 18	66	-2
Parahippocampal gyrus	L	-22.5, 4.5, -24	73	-1.9
Fusiform gyrus	R	48, -61.5, -22.5	78	-1.8
Parahippocampal gyrus	R	19.5, 3, -24	108	-1.8
Middle temporal gyrus	R	52.5, -54, -13.5	53	-1.8

L/R: Left/Right
