# Supplementary material for: Multivariate Imaging Genetics Study of MRI Gray Matter Volume and SNPs Reveals Biological Pathways Correlated with Brain Structural Differences in Attention Deficit Hyperactivity Disorder
Source: Front Psychiatry. 2016 Jul 25;7:128. doi: 10.3389/fpsyt.2016.00128 (PMC4959119; doi:10.3389/fpsyt.2016.00128)
Supplement: Supplementary file 5 [file Table_5.PDF]

Supplementary Table 5. Significant regions of brain phenotype component S2

Region	Hemisphere	Peak coordinate	Cluster Size	Z-value
Putamen/caudate	R	24, 12, -4.5	1095	9.4
Putamen/caudate	L	-22.5, 9, -3	1435	8.3
Mid-cingulate/ posterior cingulate/ precuneus	L	-9, -18, 42	2604	2.9
Thalamus	R	12, -30, 3	162	2.7
Rostral anterior cingulate/ orbitofrontal gyri	R	6, 36, -9	64	2.1
Cerebellum	R	21, -76.5, -36	98	2.1
Posterior cingulate	R	7.5, 12, 37.5	112	1.8
Inferior temporal/ Fusiform gyri	L	-58.5, -45, -21	2299	-2.5
Inferior/middle temporal gyri	R	51, -36, -22.5	417	-2.2
Inferior temporal/ Fusiform gyri	R	46.5, -9, -40.5	272	-2.1
Cerebellum	L	-37.5, -46.5, -49.5	179	-1.8

L/R: Left/Right
