# Supplementary material for: Multivariate Imaging Genetics Study of MRI Gray Matter Volume and SNPs Reveals Biological Pathways Correlated with Brain Structural Differences in Attention Deficit Hyperactivity Disorder
Source: Front Psychiatry. 2016 Jul 25;7:128. doi: 10.3389/fpsyt.2016.00128 (PMC4959119; doi:10.3389/fpsyt.2016.00128)
Supplement: Supplementary file 6 [file Table_6.PDF]

Supplementary Table 6. Significant regions of brain phenotype component S3.

Region	Hemishphere	Peak coordinate	Cluster Size	Z-value
Mid-cingulate	L	-7.5, -43.5, 36	2986	4.9
Cerebellum	R	18, -78, -34.5	1712	4.3
Caudate	R	15, 18, 3	135	3.2
Cerebellum	L	-18, -78, -36	1073	3.1
Thalamus	R	7.5, -21, 7.5	230	2.9
Thalamus	L	-7.5, -25.5, 6	154	2.8
PHG	R	30, -39, -13.5	257	2.8
Lingual gyrus	L	-16.5, -52.5, 1.5	213	2.7
Caudate	L	-12, 15, 3	136	2.6
Lingual gyrus	R	19.5, -52.5, 3	331	2.6
Lingual gyrus	L	-12, -64.5, -4.5	73	2.4
Anterior cingulate	L	-6, 13.5, 36	127	2.3
Fusiform	L	-28.5, 0, -46.5	1768	-3.1
STG	R	45, 12, -22.5	638	-2.7
Fusiform	R	31.5, -3, -46.5	1368	-2.3
STG	R	31.5, 6, -18	156	-2.3
Insula	R	42, 15, -1.5	240	-2.2
Insula	L	-42, 15, -3	72	-2.1
Amygdala	L	-27, 4.5, -18	114	-2.1
Cerebellum	L	-27, -51, -49.5	114	-2.1
MTG	L	-60, -31.5, -15	53	-1.8

PHG: Parahippocampus gyrus; MTG: Middle temporal gyrus; STG: Superior temporal gyrus; R/L: Right/Left.
