# Supplementary material for: Multivariate Imaging Genetics Study of MRI Gray Matter Volume and SNPs Reveals Biological Pathways Correlated with Brain Structural Differences in Attention Deficit Hyperactivity Disorder
Source: Front Psychiatry. 2016 Jul 25;7:128. doi: 10.3389/fpsyt.2016.00128 (PMC4959119; doi:10.3389/fpsyt.2016.00128)
Supplement: Supplementary file 7 [file Image_1.PDF]

Supplementary Material

Multivariate imaging-genetics study of MRI gray matter volume and SNPs reveals biological pathways correlated with brain structural differences in Attention Deficit Hyperactivity Disorder

Sabin Khadka M.S., Godfrey D. Pearlson M.D., Vince D. Calhoun Ph.D, Jingyu Liu Ph.D, Joel Gelernter M.D, Katie L. Bessette B.A, Michael C. Stevens Ph.D*

*Correspondence: Michael C. Stevens: Michael.Stevens@hhchealth.org

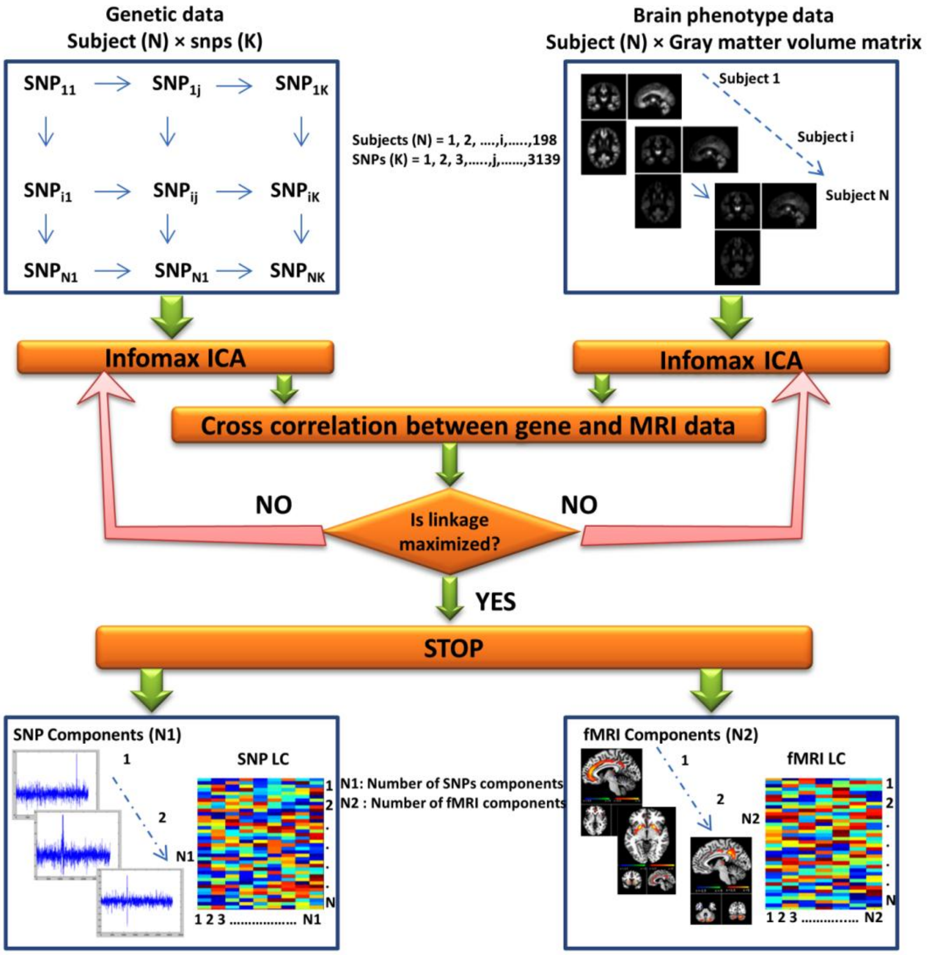

Supplementary Figure 1. Overview of Parallel ICA method to derive genotype-phenotype relationship.
