# Supplementary material for: Multivariate Imaging Genetics Study of MRI Gray Matter Volume and SNPs Reveals Biological Pathways Correlated with Brain Structural Differences in Attention Deficit Hyperactivity Disorder
Source: Front Psychiatry. 2016 Jul 25;7:128. doi: 10.3389/fpsyt.2016.00128 (PMC4959119; doi:10.3389/fpsyt.2016.00128)
Supplement: Supplementary file 8 [file Image_2.PDF]

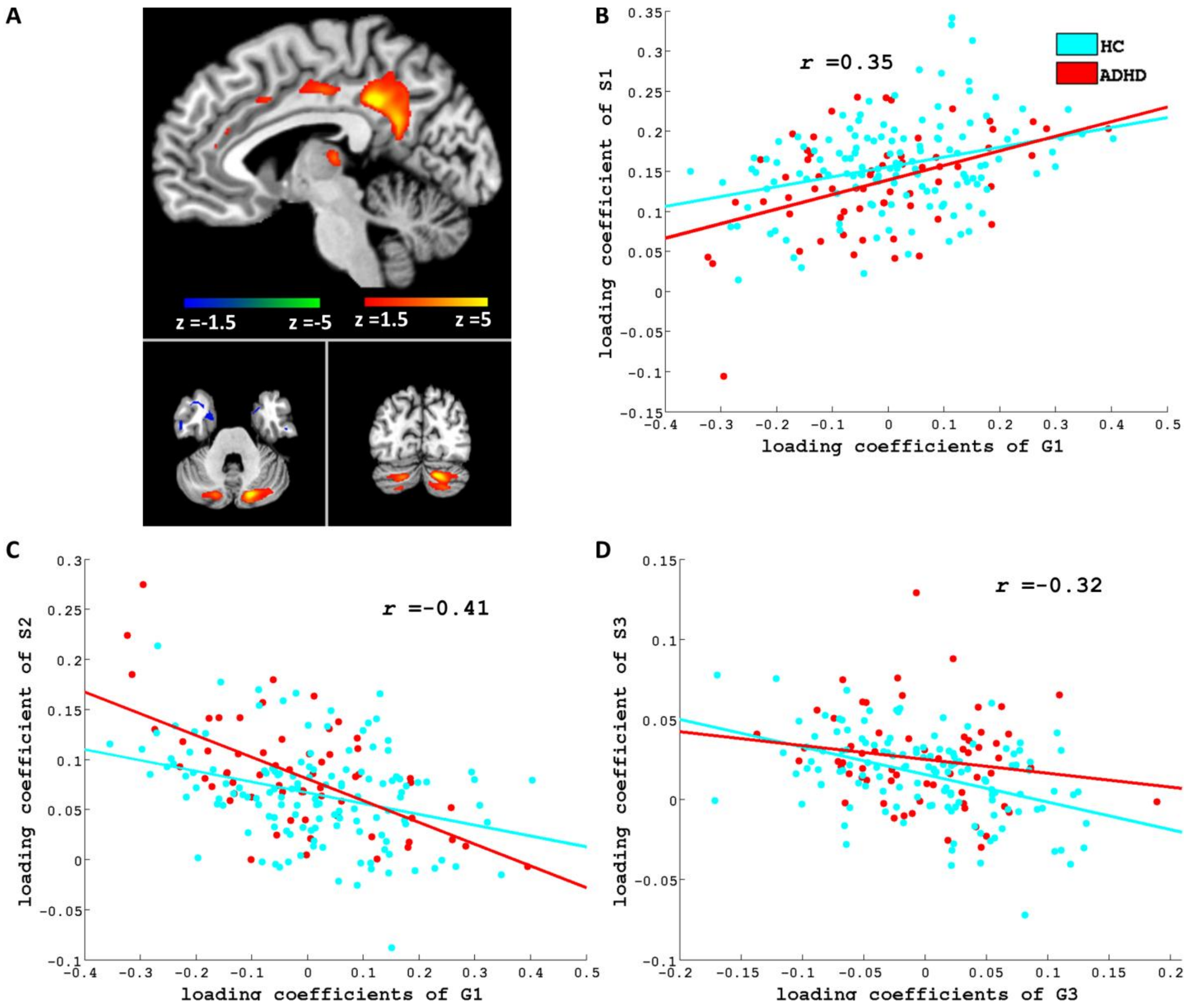

Supplementary Figure 2. (A) Significant regions in brain phenotype component S3. Brain slices shown in the above figure are x =-5, y =-79 and z =-34 in Montreal Neurological Institute (MNI) space. Scatter plots of loading coefficient of (B) brain phenotype component S1 and genetic component G1 (C) brain phenotype component S2 and genetic component G1 (D) brain phenotype component S3 and genetic component G3. Scatter plot and line in red and black indicates ADHD and HC group respectively.
